# Product Distributions of Cytochrome P450 OleT_JE_ with Phenyl-Substituted Fatty Acids: A Computational Study

**DOI:** 10.3390/ijms22137172

**Published:** 2021-07-02

**Authors:** Yen-Ting Lin, Sam P. de Visser

**Affiliations:** 1 Manchester Institute of Biotechnology, The University of Manchester, Manchester M1 7DN, UK; yen-ting.lin@manchester.ac.uk; 2 Department of Chemical Engineering and Analytical Science, The University of Manchester, Manchester M13 9PL, UK

**Keywords:** biocatalysis, enzyme mechanism, cytochrome P450, hydroxylation, desaturation, biofuel

## Abstract

There are two types of cytochrome P450 enzymes in nature, namely, the monooxygenases and the peroxygenases. Both enzyme classes participate in substrate biodegradation or biosynthesis reactions in nature, but the P450 monooxygenases use dioxygen, while the peroxygenases take H_2_O_2_ in their catalytic cycle instead. By contrast to the P450 monooxygenases, the P450 peroxygenases do not require an external redox partner to deliver electrons during the catalytic cycle, and also no external proton source is needed. Therefore, they are fully self-sufficient, which affords them opportunities in biotechnological applications. One specific P450 peroxygenase, namely, P450 OleT_JE_, reacts with long-chain linear fatty acids through oxidative decarboxylation to form hydrocarbons and, as such, has been implicated as a suitable source for the biosynthesis of biofuels. Unfortunately, the reactions were shown to produce a considerable amount of side products originating from C^α^ and C^β^ hydroxylation and desaturation. These product distributions were found to be strongly dependent on whether the substrate had substituents on the C^α^ and/or C^β^ atoms. To understand the bifurcation pathways of substrate activation by P450 OleT_JE_ leading to decarboxylation, C^α^ hydroxylation, C^β^ hydroxylation and C^α^–C^β^ desaturation, we performed a computational study using 3-phenylpropionate and 2-phenylbutyrate as substrates. We set up large cluster models containing the heme, the substrate and the key features of the substrate binding pocket and calculated (using density functional theory) the pathways leading to the four possible products. This work predicts that the two substrates will react with different reaction rates due to accessibility differences of the substrates to the active oxidant, and, as a consequence, these two substrates will also generate different products. This work explains how the substrate binding pocket of P450 OleT_JE_ guides a reaction to a chemoselectivity.

## 1. Introduction

With the advent of knowledge on biochemical reaction processes and mechanisms, the use of biosystems in engineering processes has increased rapidly. In particular, enzymes are now commonly applied in industrial processes related to food production as well as the synthesis of fine-chemicals. One area of great potential for enzymes is the field of biofuels, where they can be utilized for the synthesis of hydrocarbons. Interestingly, in nature, there are several enzymes involved in the conversion of alcohols or fatty acids into hydrocarbons [1,2,3,4,5,6], and, as such, there is potential to use these for biotechnical applications. Most of these enzymes are metalloenzymes, where a reaction on either an iron or copper center takes place. Metalloenzymes are highly versatile in nature and catalyze substrate oxidation and reduction reactions efficiently [7,8,9]. In particular, the oxygen-activating mononuclear iron-containing enzymes are useful in this respect. They have been assigned to two different categories, namely, the heme monooxygenases [10,11,12,13,14,15,16,17,18] and the nonheme iron dioxygenases [19,20,21,22,23,24,25,26], where the former group includes the cytochromes P450 involved in biosynthesis and biodegradation processes.

**Scheme 1:**
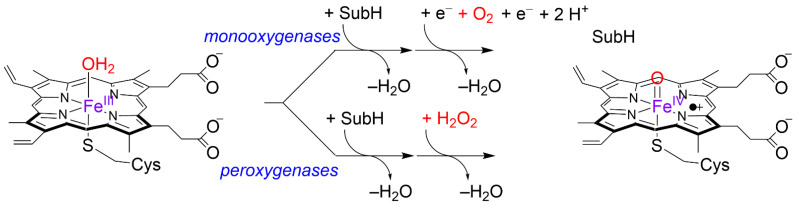
Reaction mechanism for the formation of the active species in cytochrome P450 enzymes (Compound I) in monooxygenases using dioxygen (top reaction) or in peroxygenases using H_2_O_2_ (lower reaction); SubH is the substrate.

The cytochromes P450 are heme enzymes that either take O_2_ and react as monooxygenases or use H_2_O_2_ and operate as peroxygenases. P450 monooxygenases react dioxygen on an iron (III)-heme center to form an iron (IV)-oxo heme cation radical species called Compound I (CpdI) with the help of two external electrons (from redox partners) and two protons from the solvent according to the mechanism shown in Scheme 1. CpdI is a versatile catalyst that reacts with aliphatic chains through substrate hydroxylation or desaturation, or with olefins and arenes to form epoxides and phenols. In addition to the P450 monooxygenases, there are several P450s that use H_2_O_2_ instead of dioxygen and therefore act as peroxygenases. These also form CpdI in their catalytic cycle but do not need external electrons and protons as they are provided by H_2_O_2_ (bottom reaction in Scheme 1). As such, these P450 peroxygenases are self-sufficient and more suitable for biotechnological applications than the P450 monooxygenases. The P450 peroxygenases were reported to take long-chain fatty acids as substrates and react them through an oxidative decarboxylation to form terminal olefins [27,28,29,30,31,32,33,34,35,36,37].

**Scheme 2:**
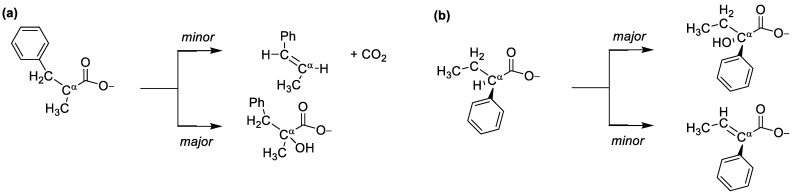
Substrate activation of phenyl-substituted fatty acids by P450 OleTJE: (**a**) Activation of 2-methyl-3-phenylpropionate. (**b**) Activation of 2-phenylbutyrate.

P450 peroxygenases were also shown to react with a variety of small carboxylic acids; however, the product distributions were markedly different between the various substrates [36]. Thus, Pickl et al. [36] showed that P450 OleT_JE_ reacts with linear fatty acids, and, e.g., a substrate with the chain length of 13 carbon atoms produced dominant decarboxylation products to form the C_12_ olefin with a minor amount of C^β^ hydroxylation. By contrast, using *R*- or *S*-2-methylbutyrate instead, P450 OleT_JE_ converted the substrate to the C^α^-hydroxylated product or caused desaturation of the C^α^–C^β^ bond. Computational modeling on the mechanism of the latter reaction showed that the substrate was able to bind in various conformations that led to different reaction channels and products that are close in energy [36,38]. Yet, when P450 OleT_JE_ was fed with 2-methyl-3-phenylpropionate, the reaction products corresponded to decarboxylation and C^α^ hydroxylation instead [36], as shown in Scheme 2. By contrast, the reaction of P450 OleT_JE_ with 2-phenylbutyrate gave a major reaction channel for C^α^ hydroxylation and a minor amount of C^α^–C^β^ desaturation. Clearly, the reaction products of substrate activation by P450 OleT_JE_ are highly dependent on the size and shape of the substrate, and the origins of these product distributions remain unclear. Therefore, we decided to conduct another computational study into substrate activation by P450 OleT_JE_, investigate substrates with phenyl substituents and find out how P450 OleT_JE_ would activate these. In particular, no computational studies have been reported on fatty acids with phenyl side chains that create bulkier substrates than linear long-chain fatty acids and hence may not fit the substrate binding pocket well.

## 2. Results

**Figure 1:**
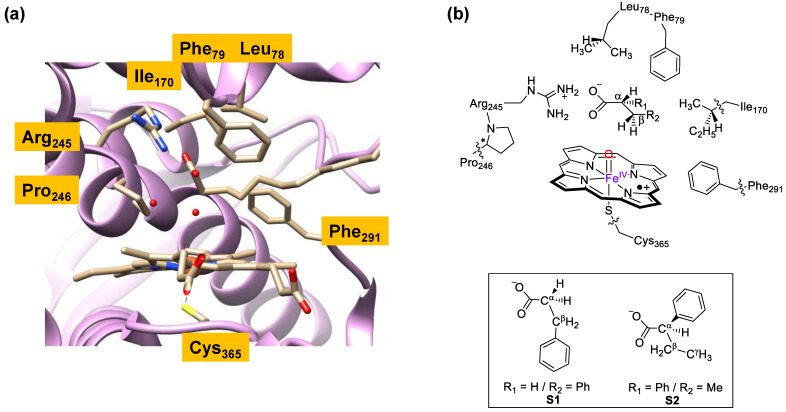
(**a**) Active site of the crystal structure coordinates of the substrate-bound form of P450 OleT_JE_ from the 4L40 protein databank file with key residues highlighted; (**b**) cluster models of the active site of P450 OleT_JE_ as investigated in this work with the substrates S1 and S2 shown in the inset.

To understand the mechanism of substrate activation by P450 OleT_JE_ enzymes and, in particular, how the enzymes react with fatty acid substrates with phenyl substituents, we decided to create a series of cluster models and study the possible reaction pathways for fatty acid activation by CpdI models. Several previous studies covered the H_2_O_2_ activation on a heme center to form CpdI [39,40,41,42], which is the active oxidant that performs the catalysis. Hence, we will focus on the mechanism starting from CpdI and a substrate only. Previous studies of ours showed that large QM cluster models of well over 150 atoms represent the oxidant and substrate binding pockets of enzymes highly accurately and give results at par with QM/MM methods [43,44,45,46]. Therefore, a large QM cluster model of the substrate-bound P450 OleT models was set up. The substrate-bound structure of P450 OleT_JE_ was characterized crystallographically by Belcher et al. [47], and an extract of the 4L40 protein databank file [48] (pdb) is shown in Figure 1. As it can be seen, the heme is linked to the protein through a thiolate linkage of Cys_365_. The substrate binding pocket is mostly lined with aromatic and aliphatic amino acids including the side chains of Leu_78_, Phe_79_, Ile_170_, Pro_246_ and Phe_291_. In the substrate binding pocket, the carboxylate of the icosanoic acid substrate forms a salt bridge with the side chain of Arg_245_.

To understand the substrate activation mechanism, we set up cluster models using previously reported methods that take key hydrogen bonding and polar residues into consideration [49,50]. In order to do so, we removed the icosanoic acid from the pdb and docked an alternative substrate manually into the pocket. These substrates lack the long aliphatic tail of icosanoic acid, and hence the models we created only included protein residues within the vicinity of the new substrates. In particular, we created two reactant models, namely, **Re**_S1_ and **Re**_S2_, which have the same protein structure but contain a different substrate, i.e., either **S1** or **S2**, Figure 1b. Thus, substrate **S1** is 3-phenylpropionate and substrate **S2** is 2-phenylbutyrate. Model **Re**_S1_ has 162 atoms in total, whereas model **Re**_S2_ contains 165 atoms. Both systems have a neutral charge and were calculated in the lowest energy doublet and quartet spin states, as identified with the superscript of 2 or 4 next to the labels. Following previous experience with substrate binding into the active site of P450 OleT_JE_ [36], we made sure the substrate forms a double hydrogen bonding interaction and salt bridge of its carboxylate group with the methylguanidinium group of Arg_245_. In addition, the model contains the heme with all ring substituents truncated to hydrogen atoms and the axial Cys_365_ residue as thiolate. The substrate binding pocket contains the peptide dimers Leu_78_-Phe_79_ and Arg_245_-Pro_246_ as well as the side chains of residues Ile_170_ and Phe_291_. A previous study used the same peptide and active site model but with hexanoate as substrate [38], whereby the model was validated against high-level quantum mechanics/molecular mechanics (QM/MM) calculations [51,52] and large cluster models [53]. These studies found very good agreement of the calculations of energy landscapes for a reaction mechanism, as obtained with either cluster models or QM/MM. In addition, a range of computational methods were tested, namely, several density functional theory methods and basis sets, and all approaches came to the same conclusions and predicted similar rate constants. As such, we will use large cluster models here with the B3LYP method in combination with a double-ζ quality basis set on all atoms, i.e., LACVP basis set with core potential on iron and 6-31G on the rest of the atoms, while a solvent model with a dielectric constant of ε = 5.7 was included in the geometry optimizations, as well, as that enables a comparison with previous work in the field.

**Figure 2:**
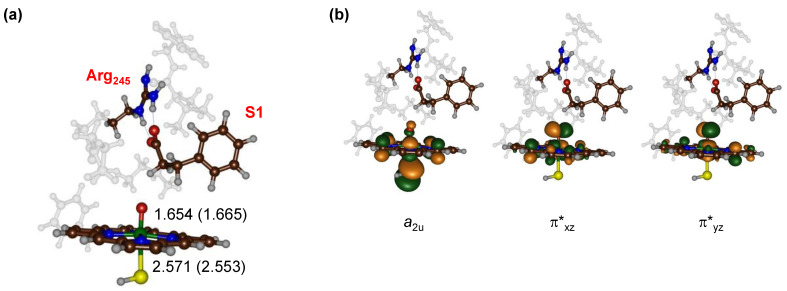
(**a**) Optimized geometries of the **S1**-bound reactant complexes ^4^**Re**_S1_ (^2^**Re**_S1_) with bond lengths in angstroms; (**b**) singly occupied molecular orbitals of ^4,2^**Re**_S1_.

The optimized geometry of our CpdI model with substrate **S1** bound (**Re**_S1_) is shown in Figure 2a for the doublet and quartet spin states. Geometrically, the doublet and quartet spin state structures are very similar with almost equal Fe–O distances of 1.665 and 1.654 Å and Fe–S interactions of 2.553 and 2.571 Å, respectively. This is not surprising as the two spin states are known to be close in energy [54,55,56,57,58,59,60] in CpdI and have the same orbital occupation with three unpaired electrons in orbitals labeled *a*_2u_, π*_xz_ and π*_yz_ (Figure 2b). The *a*_2u_ orbital is a heme-based orbital that in D_4h_ symmetry has contributions on the pyrrole nitrogen atoms and the *meso*-carbon atoms. In addition, it mixes strongly with a lone pair orbital on the sulfur atom of the axial ligand, which gives it its oxidative power. The π*_xz_ and π*_yz_ orbitals are the antibonding orbitals along the Fe–O bond. Similar structures and orbital occupations were obtained for the reactant complexes with substrate **S2** bound (**Re**_S2_). Overall, the reactant structure and electronic configuration match previous computational studies on P450 CpdI excellently and predict similar bond lengths to those seen previously for analogous systems [36,38,51,52,53,54,55,56,57,58,59,60,61,62,63,64,65,66,67,68,69,70,71,72,73,74,75,76,77,78].

**Scheme 3:**
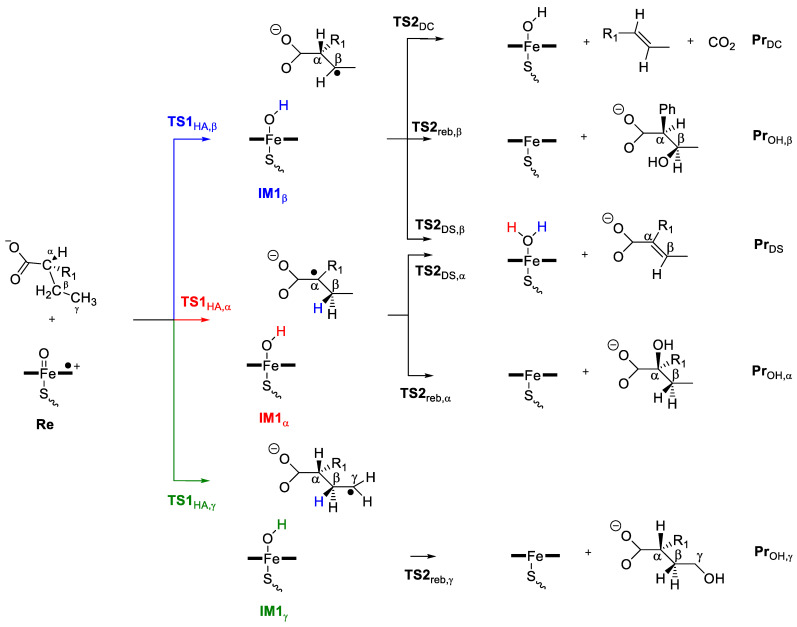
Reaction mechanisms explored for substrate activation by P450 OleT_JE_ with definition of the labels for the intermediates, products and transition states.

Next, we explored the substrate activation channels of **S1** and **S2** by CpdI, and the details of the explored reaction mechanisms are shown in Scheme 3 with the designated labeling of the individual structures. Thus, structures related to substrate **S1** have S1 in subscript after the label, while those associated with substrate **S2** have S2 in subscript after the label. In particular, we tested the activation of the C^α^–H and C^β^–H bonds of both substrates, while for **S2**, we also investigated the products originating from C^γ^–H activation. These reaction pathways start with an initial hydrogen atom abstraction from either the C^α^–H, C^β^–H or C^γ^–H (if applicable) positions via transition states **TS1**_HA,__α_, **TS1**_HA,__β_ and **TS1**_HA,__γ_ to form the radical intermediates designated **IM1**_α_, **IM1**_β_ and **IM1**_γ_. As it is usually seen in substrate hydroxylation pathways [55,79,80], an OH rebound barrier via **TS2**_reb,__α_, **TS2**_reb,__β_ or **TS2**_reb,__γ_ then gives C^α^ hydroxylation, C^β^ hydroxylation and C^γ^ hydroxylation products (**Pr**_OH,__α_, **Pr**_OH,__β_ and **Pr**_OH,__γ_), respectively. In addition, from either **IM1**_α_ or **IM1**_β_ a second hydrogen atom abstraction can take place via transition states **TS2**_DS,__α_ and **TS2**_DS,__β_ to form a double bond along the C^α^–C^β^ bond and give a desaturated product (**Pr**_DS_). A final mechanism through substrate decarboxylation is only possible from **IM1**_β_ via transition state **TS2**_DC_ to form CO_2_, olefin and an iron(III)–hydroxo(heme) complex, **Pr**_DC_. We investigated all these individual reaction pathways for the cluster model **Re** with substrates **S1** and **S2** bound in the quartet spin state as previous work showed the quartet spin state to be the most sensitive to bifurcation pathways [52,81]. 

### 2.1. 3-Phenylpropionate Activation by CpdI of P450 OleT_JE_

**Figure 3:**
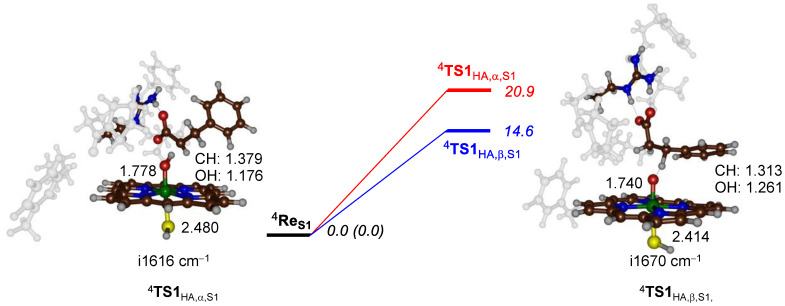
Hydrogen atom abstraction steps from substrate **S1** by CpdI of P450 OleT_JE_. The middle panel shows relative energies in kcal mol^−1^ as obtained at the ΔE_BS2_+ZPE_BS1_ level of theory. Hydrogen atom abstraction transition state structures are shown on the side with bond lengths in angstroms and the imaginary frequency in the transition state in cm^−1^.

Next, a mechanistic study into 3-phenylpropionate (**S1**) activation by the CpdI model of P450 OleT_JE_ was performed using model **Re**_S1_ leading to decarboxylation, desaturation, α-hydroxylation and β-hydroxylation products, as described above in Scheme 3. These four reaction products all originate from either C^α^–H or C^β^–H hydrogen atom abstraction; hence, we will start with the hydrogen atom abstraction steps first. Figure 3 shows the hydrogen atom abstraction transition state structures **TS1**_HA,__α,S1_ and **TS1**_HA,__β,S1_ as geometries optimized with density functional theory methods. In addition, the middle panel of Figure 3 shows the energy profile for relative enthalpies (ΔE+ZPE; ZPE = zero-point energy). The C^β^–H hydrogen atom abstraction barrier is considerably lower in energy than the C^α^–H hydrogen atom abstraction barrier: ΔE+ZPE = 14.6 kcal mol^−1^ for **TS1**_HA,__β,S1_ and ΔE+ZPE = 20.9 kcal mol^−1^ for **TS1**_HA,__α,S1_. Therefore, **S1** activation by CpdI of P450 OleT_JE_ will give dominant products from C^β^–H activation. The barrier for **TS1**_HA,__β,S1_ is close in energy to the one calculated by a minimal cluster model without protein residues for C^β^–H activation of propane or the activation of the methyl C–H bond of *trans*-methyl-phenylcyclopropane [82,83,84]. The fact that the barrier **TS1**_HA,__α,S1_ is much higher in energy than those barriers obtained with the minimal model and *trans*-methyl-phenylcyclopropane means that the second coordination sphere of the enzyme, i.e., the substrate binding pocket, restricts the substrate positioning and hampers C^α^–H hydrogen atom abstraction dramatically, raising it in energy.

In general, both transition state structures are late, with long C–H bonds of 1.379 Å (**TS1**_HA,__α,S1_) and 1.313 Å (**TS1**_HA,__β,S1_), whereas the accepting O–H bond is much shorter: 1.176 Å for **TS1**_HA,__α,S1_ and 1.261 Å for **TS1**_HA,__β,S1_. In both transition states, the Fe–O distance has elongated from 1.654 in the reactant complex to 1.778 Å in **TS1**_HA,__α,S1_ and 1.740 Å in **TS1**_HA,__β,S1_. This is due to the formation of the O–H bond which polarizes the π*_yz_ orbital more to the iron and hence makes the Fe–O bond weaker. Both transition states are characterized by a large imaginary frequency for the O–H–C stretch vibration, with a magnitude of i1616 cm^−1^ (**TS1**_HA,__α,S1_) and i1670 cm^−1^ (**TS1**_HA,__β,S1_). These large imaginary frequencies implicate that the potential energy surface is steep and gives a narrow-width peak that could result in a large kinetic isotope effect due to quantum mechanical tunneling and that will be sensitive to the substitution of hydrogen by deuterium atoms.

**Figure 4:**
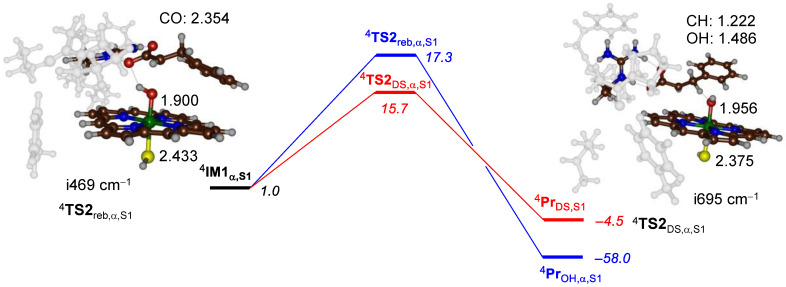
Substrate desaturation and hydroxylation bifurcation pathways for substrate **S1** from the radical intermediate **IM1**_α,S1_ of P450 OleT_JE_. The middle panel gives energies relative to ^4^**R**e_S1_ in kcal mol^−1^ as obtained at the ΔE+ZPE level of theory. Transition state structures are shown on the side with bond lengths in angstroms and the imaginary frequency in the transition state in cm^−1^.

The transition states relax to radical intermediates **IM1**_α,S1_ and **IM1**_β,S1_ that react further past the various bifurcation channels. Let us start with the pathways from **IM1**_α,S1_ that can lead to desaturation of the C^α^–C^β^ bond via a second hydrogen atom abstraction or OH rebound to give the alcohol product complex; the optimized geometries and the energy landscape are shown in Figure 4. The **IM1**_α,S1_ intermediate is only 1 kcal mol^−1^ higher in energy than the reactant complex **Re**_S1_ and has the transition states leading to the two reaction pathways to products within 2 kcal mol^−1^. In particular, the lowest barrier leads to desaturation through a second hydrogen atom abstraction from C^β^–H via a transition state of ΔE+ZPE = 15.7 kcal mol^−1^. By contrast, the OH rebound barrier is ΔE+ZPE = 17.3 kcal mol^−1^. These close barriers implicate that a mixture of products may be expected for **S1** activation by P450 OleT_JE_ enzymes, which indeed is what has been observed experimentally [36]. Interestingly, the reaction leading to hydroxylation products is considerably more exothermic than the one leading to desaturation by more than 50 kcal mol^−1^. As such, the protein environment hampers OH rebound and affects the kinetics of the substrate activation process in favor of a much lesser exothermic pathway. This implies that P450 OleT_JE_ reacts through negative catalysis, where an otherwise highly exothermic pathway is blocked in favor of a lesser exothermic reaction channel [85,86,87]. 

The transition states have typical features of OH rebound and desaturation barriers and match previous work on substrate hydroxylation and desaturation by P450 enzymes [36,38,51,52,53,64,65,66,67,68,69,72,79,88,89]. The rebound transition state has a long C–O distance of 2.354 Å, although the imaginary frequency (i469 cm^−1^) is clearly a pure C–O stretch vibration. As a matter of fact, a series of rebound calculations by biomimetic iron(III)–hydroxo and manganese(III)–hydroxo complexes have similarly long C–O bonds in the transition states [90,91]. In the transition state, the Fe–O bond further elongates to 1.900 Å. In the desaturation transition state **TS2**_DS,__α,S1_, the same thing happens, and Fe–O elongates to 1.956 Å. Interestingly, the desaturation transition state is more reactant-like, with a shorter C–H (1.222 Å) than O–H (1.486 Å) distance.

**Figure 5:**
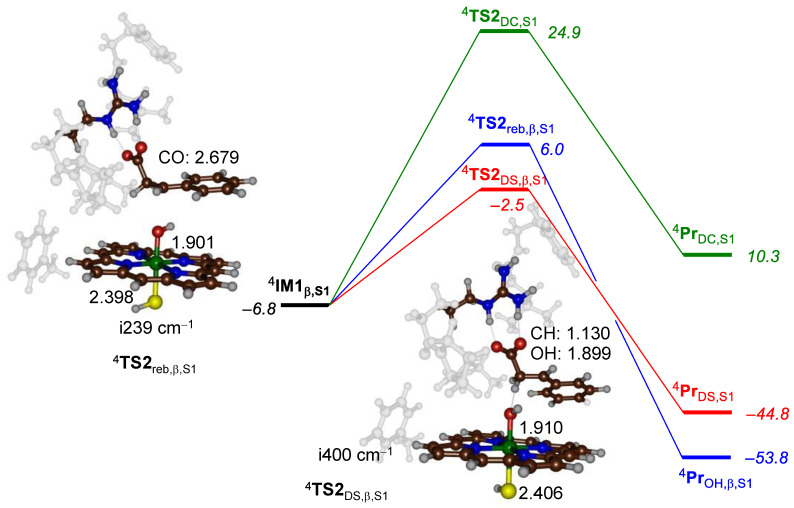
Substrate desaturation, decarboxylation and hydroxylation bifurcation pathways for substrate **S1** from the radical intermediate **IM1**_β,S1_ of P450 OleT_JE_. The energy landscape gives energies relative to ^4^**R**e_S1_ in kcal mol^−1^ as obtained at the ΔE+ZPE level of theory. Transition state structures are shown with bond lengths in angstroms and the imaginary frequency in the transition state in cm^−1^.

Subsequently, we investigated the pathways from the alternative radical intermediate **IM1**_β,S1_ leading to desaturation, decarboxylation and β-hydroxylation. Figure 5 shows the potential energy profile for the pathways from **IM1**_β,S1_ leading to products and the various transition states that were characterized. The lowest energy barrier (of ΔE+ZPE = 4.3 kcal mol^−1^ above **IM1**_β,S1_) involves a second hydrogen atom abstraction and the formation of the desaturated products. At least 8.5 kcal mol^−1^ above the desaturation barrier is the OH rebound barrier **TS2**_reb,__β,S1_, while the decarboxylation barrier is even higher (more than 20 kcal mol^−1^ above the desaturation barrier).

The rebound and desaturation barriers **TS2**_reb,__β,S1_ and **TS2**_DS,__β,S1_ are shown in Figure 5. In both structures, the Fe–O distance is long: 1.901 and 1.910 Å for **TS2**_reb,__β,S1_ and **TS2**_DS,__β,S1_, respectively, which matches the structures in Figure 4 well. The rebound C–O distance is even longer in **TS2**_reb,__β,S1_ than in **TS2**_reb,__α,S1_ and is 2.679 Å. Nevertheless, the imaginary mode in the transition state (of i239 cm^−1^) represents an O–C stretch vibration; hence, it is appropriate for a rebound barrier. Similar to **TS2**_DS,__α,S1_, **TS2**_DS,__β,S1_ is also reactant-like, with a short C–O distance of 1.130 Å and a much longer O–H distance of 1.899 Å. Overall, these structures match previous barriers and landscapes calculated on analogous models but with different substrates.

### 2.2. 2-Phenylbutyrate Activation by CpdI of P450 OleT_JE_

**Figure 6:**
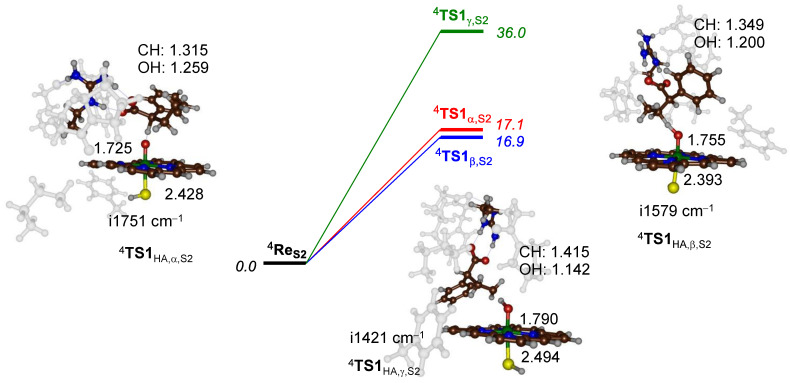
Hydrogen atom abstraction steps from substrate **S2** by CpdI of P450 OleT_JE_. The middle panel gives relative energies in kcal mol^−1^ as obtained at the ΔE+ZPE level of theory. Hydrogen atom abstraction transition state structures are shown on the side with bond lengths in angstroms and the imaginary frequency in the transition state in cm^−1^.

To understand the effect of phenyl substitution to fatty acid substrates of P450 OleT_JE_, we also investigated 2-phenylbutyrate activation by our CpdI model. For this particular system, we tested hydrogen atom abstraction pathways from the C^α^–H, C^β^–H and C^γ^–H positions; the obtained transition states and energy landscape for these three steps are shown in Figure 6. As it can be seen, the hydrogen atom abstraction barriers ^4^**TS1**_HA,__α,S2_ and ^4^**TS1**_HA,__β,S2_ are within 1 kcal mol^−1^ in energy, although inclusion of entropy and thermal corrections appears to favor the C^α^–H pathway slightly. The C^γ^–H hydrogen atom abstraction barrier, on the other hand, is well higher in energy (by ΔE+ZPE = 19.1 kcal mol^−1^) than ^4^**TS1**_HA,__β,S2_; consequently, this pathway is unlikely to happen, and we do not expect products originating from C^γ^–H hydrogen atom abstraction.

The optimized geometries of ^4^**TS1**_HA,__α,S2_, ^4^**TS1**_HA,__β,S2_ and ^4^**TS1**_HA,__γ,S2_ are presented alongside the landscape in Figure 6. All three structures have their Fe–S distances in the window from 2.393 to 2.494 Å, while the Fe–O distance ranges from 1.725 for ^4^**TS1**_HA,__α,S2_ to 1.790 Å for ^4^**TS1**_HA,__β,S2_. All three transition states are product-like, with shorter O–H than C–H distances. Moreover, they are all characterized with a large imaginary frequency that implies a significant amount of tunneling will take place and probably the kinetics will be affected by isotopic substitution of hydrogen atoms by deuterium in the substrate. 

**Figure 7:**
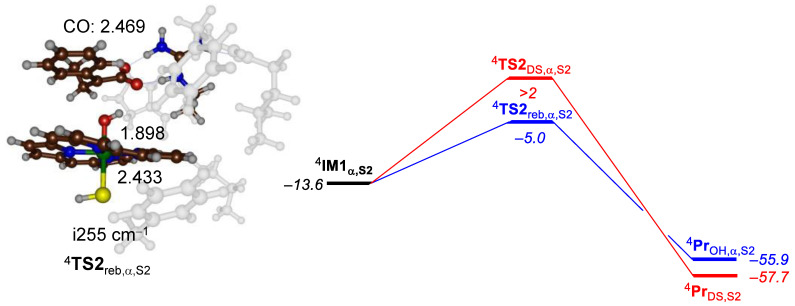
Substrate desaturation and hydroxylation bifurcation pathways for substrate **S2** from the radical intermediate **IM1**_α,S2_ of P450 OleT_JE_. The right-hand side shows energies relative to ^4^**R**e_S2_ in kcal mol^−1^ as obtained at the ΔE+ZPE level of theory. The rebound transition state structure is shown on the left with bond lengths in angstroms and the imaginary frequency in the transition state in cm^−1^. The desaturation transition state energy was estimated from constraint geometry scans.

Next, we investigated the bifurcation pathways from each of the radical intermediates ^4^**IM1**_α,S2_, ^4^**IM1**_β,S2_ and ^4^**IM1**_γ,S2_. The energy landscape for the product formation step from ^4^**IM1**_α,S2_ is shown in Figure 7, and two possibilities were considered, namely, OH rebound to form the C^α^ alcohol or desaturation of the C^α^–C^β^ bond. The lowest energy pathway is the rebound step with a barrier of about 8.6 kcal mol^−1^ above ^4^**IM1**_α,S2_. This is a significant barrier as usually rebound barriers are much smaller in energy and typically have a magnitude between 0 and 4 kcal mol^−1^ [79,91]. Clearly, the protein environment and hydrogen bonding interactions restrict the OH rebound and hamper and slow this process down. Nevertheless, our prediction that the products originating from ^4^**IM1**_α,S2_ should give α-hydroxylation matches experimental observations excellently as those were the dominant products obtained for this substrate in reaction with P450 OleT_JE_ [36].

The rebound transition state is similar in structure to the one shown above in Figure 4, with a long C^α^–O distance of 2.469 Å and a small imaginary frequency of i255 cm^−1^ representing the C^α^–O stretch vibration. In agreement with the rebound transition states mentioned above, the Fe–O distance has elongated and is 1.898 Å in ^4^**TS2**_reb,__α,S2_, whereas the Fe–S distance is 2.433 Å. We also attempted to locate the transition state for the second hydrogen atom abstraction from ^4^**IM1**_α,S2_; however, the barrier is relatively high in energy, i.e., well above that for ^4^**TS2**_reb,__α,S2_, and an estimate from the geometry scan located it 15.8 kcal mol^−1^ above the radical intermediate. Consequently, we do not expect desaturation products to appear after C^α^–H hydrogen atom abstraction, and this channel will give dominant C^α^ hydroxylation products only.

**Figure 8:**
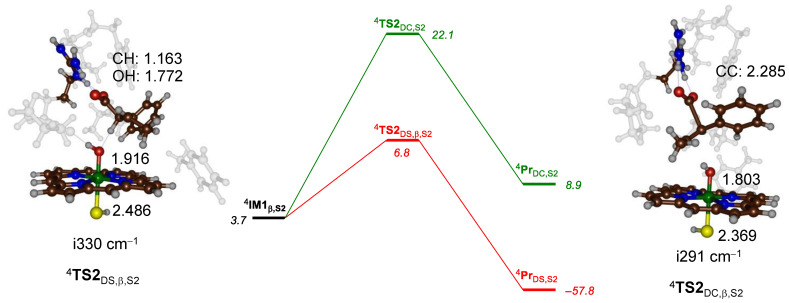
Substrate desaturation and decarboxylation bifurcation pathways for substrate **S2** from the radical intermediate **IM1**_β,S2_ of P450 OleT_JE_. The energy landscape gives energies relative to ^4^**R**e_S2_ in kcal mol^−1^ as obtained at the ΔE+ZPE level of theory. Transition state structures are shown with bond lengths in angstroms and the imaginary frequency in the transition state in cm^−1^.

The energy landscapes for the bifurcation pathways from ^4^**IM1**_β,S2_ are displayed in Figure 8. The lowest energy pathway is the second hydrogen atom abstraction leading to desaturation of the C^α^–C^β^ bond, with a barrier of ΔE+ZPE = 3.1 kcal mol^−1^ above the radical intermediate. The alternative decarboxylation mechanism, however, is high in energy and well higher by 15.3 kcal mol^−1^ and, consequently, will have a very minor role of importance for this substrate. Indeed, experimental studies on this particular system did not record any product originating from decarboxylation of the substrate, which is consistent with the results shown here. Optimized geometries of the two bifurcation transition states ^4^**TS2**_DS,__β,S2_ and ^4^**TS2**_DC,__β,S2_ are also shown in Figure 8. In both cases, the Fe–O distance has elongated with respect to the reactant complex, and it is 1.916 Å for ^4^**TS2**_DS,__β,S2_ and 1.803 Å for ^4^**TS2**_DC,__β,S2_. The carboxylation takes place at a long C–C^α^ distance of 2.285 Å but has a clear C–C stretch vibration as an imaginary frequency with a magnitude of i291 cm^−1^. A similar size imaginary frequency of i330 cm^−1^ for the desaturation barrier was recorded. This value is very small for a hydrogen atom abstraction transition state and well lower than the **TS1** transition states shown above in Figure 3 and Figure 6. Consequently, the second hydrogen atom abstraction will not be affected from the same major kinetic isotope effects as the **TS1** barriers reported above and limited quantum mechanical tunneling will incur during the **TS2**_DS_ transition. The desaturation transition state is highly reactant-like and early, with a short C–H distance of 1.163 Å and a very long O–H distance of 1.772 Å.

Overall, the calculations performed on S2 activation by our P450 OleT_JE_ models show competitive reaction channels originating from C^α^–H and C^β^–H hydrogen atom abstraction. However, the former channel leads to dominant C^α^ hydroxylation products, whereas the latter channel gives desaturation instead. As such, the calculations predict a mixture of products. This is in good agreement with the product distributions reported by Pickl et al. [36] that gave a ratio of 332:80 for C^α^ hydroxylation versus desaturation of 2-phenylbutyrate by P450 OleT_JE_. To understand the differences in product distributions obtained for S1 and S2 substrates, we analyzed the electronic properties of the various structures and investigated C–H bond strengths of the substrates. 

## 3. Discussion

This work studies substrate activation by P450 OleT_JE_ enzymes. In particular, the substrates 3-phenylpropionate (**S1**) and 2-phenylbutyrate (**S2**) and the pathways leading to α-hydroxylation, β-hydroxylation, desaturation and decarboxylation were investigated. DFT cluster models of the enzyme mechanism show that the reaction is stepwise via a radical intermediate and proceeds with a rate-determining hydrogen atom abstraction step. The absolute energies of the barriers are quite different for the two substrates, whereby for **S1**, the lowest barrier is for C^β^–H hydrogen atom abstraction (ΔE+ZPE = 14.6 kcal mol^−1^), while the C^α^–H barrier is well higher (ΔE+ZPE = 20.9 kcal mol^−1^). By contrast, for substrate **S2**, the two barriers are within 0.2 kcal mol^−1^ with a small preference of the C^β^–H channel. As such, a mixture of products is expected for **S2**. In particular, for substrate **S2**, the C^α^–H hydrogen atom abstraction is followed by a low-energy OH rebound barrier to give α-hydroxylation, whereas the C^β^–H hydrogen atom abstraction leads to desaturation with a subsequent second hydrogen atom abstraction. These predicted products, i.e., α-hydroxylation and desaturation, for substrate **S2** activation by P450 OleT_JE_ are in perfect agreement with the product distributions reported by Pickl et al. [36].

**Figure 9:**
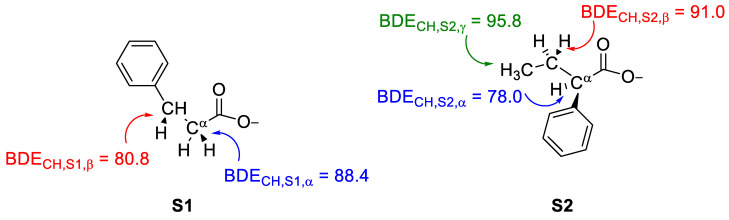
Calculated substrate BDE values for selected C–H bonds in **S1** and **S2**. Values are ΔE+ZPE in kcal mol^−1^.

To understand the difference in hydrogen atom abstraction barriers between the two substrates, we calculated C–H bond dissociation energies (BDEs) for isolated substrates in the gas phase at the B3LYP/6-311++G** level of theory (with a solvent model included). These BDEs were calculated from the adiabatic homolytic bond strengths as the difference in energy (ΔE+ZPE) of the substrate, a hydrogen atom and the substrate with a hydrogen atom removed. Previously, we showed that hydrogen atom abstraction barriers often correlate with the BDE of the substrate C–H bond that is broken [92,93,94], Figure 9. For substrate **S1**, the C^α^–H bond has a BDE_CH, S1,__α_ = 88.4 kcal mol^−1^, while the C^β^–H bond has a strength of 80.8 kcal mol^−1^. As such, the C^β^–H bond is the weakest bond in the substrate and the most likely bond to be activated by the enzyme. Indeed, our potential energy landscape (Figure 3) for **S1** activation by P450 OleT_JE_ gives a lower hydrogen atom abstraction barrier for C^β^–H than for C^α^–H, in line with the differences in the bond strengths of the two bonds in the substrate. Clearly, the protein pocket does not affect the selectivity of the reaction, and P450 OleT_JE_ appears to abstract the weakest C–H bond of the substrate.

We also calculated the C^α^–H, C^β^–H and C^γ^–H bond strengths of substrate **S2** (see Figure 9). Not surprisingly, the weakest bond is the tertiary C^α^–H bond with a BDE = 78.0 kcal mol^−1^. Much stronger are the C^β^–H bond (BDE = 91.0 kcal mol^−1^) and C^γ^–H (95.8 kcal mol^−1^). Indeed, our DFT-calculated energy landscape for hydrogen atom abstraction from these three positions returns a high energy barrier for C^γ^–H abstraction of ΔE+ZPE = 36.0 kcal mol^−1^, and hence we do not expect this reaction channel to occur. Interestingly, despite the fact that the BDEs for C^α^–H and C^β^–H are widely apart, P450 OleT_JE_ appears to be able to activate both positions, and we find almost equal hydrogen atom abstraction barriers for these pathways. Therefore, the second coordination sphere and substrate binding and positioning affect the chemoselectivity of the reaction and make the C^β^–H abstraction accessible. This is a form of negative catalysis [85,86,87] and means an otherwise high-energy reaction channel is stabilized by the protein environment and makes alternative products possible. In particular, the approach of the C^α^–H bond of **S2** on CpdI is sterically hindered through protein residues, meaning that this position is less accessible than the C^β^–H position of the substrate. The shape of the substrate binding pocket, therefore, guides the regioselectivity and the accessibility to the substrate and overrules thermochemical preferences such as the C–H bond strength.

## 4. Materials and Methods

We used density functional theory (DFT) methods as implemented in the *Gaussian*-09 software package [95]. The studies used the unrestricted hybrid density functional method B3LYP [96,97] for all calculations. The initial geometry optimizations, constraint geometry scans and analytical frequency calculations used a modest LACVP basis set on iron (with core potential) [98] coupled to 6-31G on the rest of the atoms: basis set BS1 [99]. All calculations were run with a solvent model included through the continuous polarized continuum model with a dielectric constant representing chlorobenzene (with dielectric constant ε = 5.7) [100]. The quality of the energies was improved through single point calculations with a 6-311+G* basis set on all atoms and LACV3P+ with core potential on iron: basis set BS2. Our methods have been extensively validated and benchmarked against experimental data, and these procedures were shown to reproduce experimental product distributions and rate constants well [101,102,103,104]. Full data of all results obtained is given in the Supporting Information accompanying this paper (Supplementary Materials).

## 5. Conclusions

In this work, a computational study into the activation of 3-phenylpropionate and 2-phenylbutyrate by Compound I of P450 OleT_JE_ was conducted. We set up large active site cluster models of well over 150 atoms that contain the heme and its direct ligands, the substrate and key residues in the substrate binding pocket. We then studied all mechanisms leading to C^α^ hydroxylation, C^β^ hydroxylation, C^γ^ hydroxylation, C^α^–C^β^ desaturation and decarboxylation. We showed that differences in the size and shape of the substrate give differences in orientation and, consequently, different product distributions. Thus, for 3-phenylpropionate, dominant desaturation products were produced through a rate-determining C^β^–H hydrogen atom abstraction channel. By contrast, 2-phenylbutyrate has competing hydrogen atom abstraction barriers for C^α^–H and C^β^–H abstractions and therefore gives a mixture of C^α^ hydroxylation and desaturation pathways. These predicted products are in excellent agreement with those reported previously from experimental studies on the same enzyme. The barrier heights were rationalized by comparing them to the C–H bond strengths in the substrate, and the effect of substrate binding and the second coordination sphere was discussed.

## Data Availability

Data available in a publicly accessible repository.

## Supplementary Materials

The following are available online at https://www.mdpi.com/article/10.3390/ijms22137172/s1.
