# Supplementary material for: Product Distributions of Cytochrome P450 OleTJE with Phenyl-Substituted Fatty Acids: A Computational Study
Source: Int J Mol Sci. 2021 Jul 2;22(13):7172. doi: 10.3390/ijms22137172 (PMC8269385; doi:10.3390/ijms22137172)
Supplement: Supplementary file 1 [file ijms-22-07172-s001.zip › ijms-1279252-supplementary.pdf]

Supporting Information

Product distributions of cytochrome P450 $OleT_{JE}$ with phenyl-substituted fatty acids: A computational study

Yen-Ting Lin [1,2], and Sam P. de Visser [1,2*]

[1] Manchester Institute of Biotechnology, The University of Manchester, 131 Princess Street, Manchester M1 7DN, United Kingdom; sam.devisser@manchester.ac.uk
[2] Department of Chemical Engineering and Analytical Science, The University of Manchester, Oxford Road, Manchester M13 9PL, United Kingdom; sam.devisser@manchester.ac.uk
* Correspondence: sam.devisser@manchester.ac.uk

Tables with raw data:

Model-S1 Data

Table S1: Absolute (free) energies (in au) of optimized geometries for the C^{alpha} pathway of substrate (3-phenyl propionic acid or 3-phenylpropionate; S1) activation by P450 OleT$_{JE}$ model complex as calculated at the UB3LYP level of theory.

System	BS1 (Gas)			BS2 + Solv		
	E_{BS1}	E_{BS1}+ Δ ZPE	E_{BS1}+ ΔG	$E_{BS2,solv}$ + ΔZPE	$E_{BS2,solv}$ + ΔG	$E_{BS2,Solv,D}$
**4Re S1**	-3877.974275	-3876.597295	-3876.741086	-3878.340896	-3878.484687	-3879.945016
**4TS1 HA,alpha,S1**	-3877.942710	-3876.572609	-3876.717415	-3878.307673	-3878.452478	-3879.908381
**4IM1 alpha,S1**	-3877.959274	-3876.585385	-3876.732438	-3878.339327	-3878.486379	-3879.937812
**4TS2 reb,alpha,S1**	-3877.953321	-3876.578688	-3876.722446	-3878.313365	-3878.457123	-3879.915478
**4Pr OH,alpha,S1**	-3878.049251	-3876.670304	-3876.815636	-3878.433358	-3878.578690	-3880.044469
**4TS2 DS,alpha,S1**	-3877.940821	-3876.570083	-3876.713843	-3878.315851	-3878.459612	-3879.915634
**4Pr DS,alpha,S1**	-3877.959489	-3876.586600	-3876.731703	-3878.348124	-3878.493227	-3879.946542

Table S2: Relative (free) energies (kcal mol^{-1}) of optimized geometries for the C^{alpha} pathway of substrate (3-phenyl propionic acid or 3-phenylpropionate; S1) activation by P450 OleT$_{JE}$ model complex as calculated at the UB3LYP level of theory.

System	BS1 (Gas)			BS2 + Solv		
	ΔE_{BS1}	ΔE_{BS1}+ΔZPE	ΔE_{BS1}+ΔG	$\Delta E_{BS2,Solv}$+ ΔZPE	$\Delta E_{BS2,Solv}$+ΔG	$\Delta E_{BS2,Solv,D}$+ΔZPE
**4Re S1**	0.00	0.00	0.00	0.00	0.00	0.00
**4TS1 HA,alpha,S1**	19.81	15.49	14.85	20.85	20.21	22.99
**4IM1 alpha,S1**	9.41	7.47	5.43	0.98	-1.06	4.52
**4TS2 reb,alpha,S1**	13.15	11.68	11.70	17.28	17.30	18.54
**4Pr OH,alpha,S1**	-47.05	-45.81	-46.78	-58.02	-58.99	-62.41
**4TS2 DS,alpha,S1**	20.99	17.08	17.10	15.72	15.73	18.44
**4Pr DS,alpha,S1**	9.28	6.71	5.89	-4.54	-5.36	-0.96

Table S3: Imaginary frequency (cm^{-1}) of the transition state structures along the C^{alpha} pathway of substrate (3-phenyl propionic acid or 3-phenylpropionate; S1) activation by P450 OleT$_{JE}$ model complex as calculated at the UB3LYP level of theory.

System	Imaginary Frequency (cm^{-1})
**4TS1 HA,alpha,S1**	i1616.2
**4TS2 reb,alpha,S1**	i468.9
**4TS2 DS,alpha,S1**	i536.4

Table S4: Absolute (free) energies (in au) of optimized geometries for the C^{beta} pathway of substrate (3-phenyl propionic acid or 3-phenylpropionate; S1) activation by P450 $OleT_{JE}$ model complex as calculated at the UB3LYP level of theory.

System	BS1 (Gas)			BS2 + Solv		
	E_{BS1}	E_{BS1}+ Δ ZPE	E_{BS1}+ ΔG	$E_{BS2,solv}$ + ΔZPE	$E_{BS2,solv}$ + ΔG	$E_{BS2,Solv,D}$
**4TS1 HA,beta,S1**	-3877.944838	-3876.573129	-3876.714153	-3878.317715	-3878.458740	-3879.921481
**4IM1 beta,S1**	-3877.969688	-3876.595489	-3876.741225	-3878.351745	-3878.497481	-3879.953849
**4TS2 reb,beta,S1**	-3877.944322	-3876.571254	-3876.717594	-3878.331400	-3878.477740	-3879.930088
**4 Pr OH,beta,S1**	-3878.038420	-3876.659600	-3876.805559	-3878.426672	-3878.572632	-3880.035190
**4TS2 DS,beta,S1**	-3877.947519	-3876.575138	-3876.717653	-3878.344918	-3878.487432	-3879.946595
**4 Pr DS,beta,S1**	-3878.036924	-3876.662647	-3876.808308	-3878.412305	-3878.557965	-3880.016536
**4TS2 DC,beta,S1**	-3877.920836	-3876.549187	-3876.696952	-3878.301141	-3878.448906	-3879.894080
**4 Pr DC,beta,S1**	-3877.929266	-3876.556712	-3876.704753	-3878.324456	-3878.472498	-3879.921720

Table S5: Relative (free) energies (kcal mol^{-1}) of optimized geometries for the C^{beta} pathway of substrate (3-phenyl propionic acid or 3-phenylpropionate; S1) activation by P450 $OleT_{JE}$ model complex as calculated at the UB3LYP level of theory.

System	BS1 (Gas)			BS2 + Solv		
	ΔE_{BS1}	ΔE_{BS1}+ΔZPE	ΔE_{BS1}+ΔG	$\Delta E_{BS2,Solv}$+ ΔZPE	$\Delta E_{BS2,Solv}$+ΔG	$\Delta E_{BS2,Solv,D}$+ΔZPE
**4TS1 HA,beta,S1**	18.47	15.16	16.90	14.55	16.28	11.46
**4IM1 beta,S1**	2.88	1.13	-0.09	-6.81	-8.03	-7.29
**4TS2 reb,beta,S1**	18.80	16.34	14.74	5.96	4.36	6.91
**4 Pr OH,beta,S1**	-40.25	-39.10	-40.46	-53.83	-55.19	-55.43
**4TS2 DS,beta,S1**	16.79	13.90	14.70	-2.52	-1.72	-3.88
**4 Pr DS,beta,S1**	-39.31	-41.01	-42.18	-44.81	-45.98	-46.58
**4TS2 DC,beta,S1**	33.53	30.19	27.69	24.95	22.45	28.62
**4 Pr DC,beta,S1**	28.24	25.47	22.80	10.32	7.65	11.84

Table S6: Imaginary frequency (cm^{-1}) of the transition state structure along the C^{beta} pathway of substrate (3-phenyl propionic acid or 3-phenylpropionate; S1) activation by P450 $OleT_{JE}$ model complex as calculated at the UB3LYP level of theory.

System	Imaginary Frequency (cm^{-1})
**4TS1 HA,beta,S1**	i1669.8
**4TS2 reb,beta,S1**	i238.7
**4TS2 DS,beta,S1**	i90.3
**4TS2 DC,beta,S1**	i137.4

Model-S2 Data

Table S7: Absolute (free) energies (in au) of optimized geometries for the C^{alpha} pathway of substrate (2-phenyl butanoic acid or 2-phenylbutyrate; S2) activation by P450 $OleT_{JE}$ model complex as calculated at the UB3LYP level of theory.

System	BS1 (Gas)			BS2 + Solv		
	E_{BS1}	E_{BS1}+ Δ ZPE	E_{BS1}+ ΔG	$E_{BS2,solv}$ + ΔZPE	$E_{BS2,solv}$ + ΔG	$E_{BS2,Solv,D}$
**4Re S2**	-3917.263509	-3915.858953	-3916.012330	-3917.630220	-3917.783597	-3919.263180
**4 TS1 alpha,HA,S2**	-3917.244045	-3915.844463	-3915.985709	-3917.602961	-3917.744207	-3919.238963
**4IM1 alpha,S2**	-3917.289342	-3915.884809	-3916.029864	-3917.651933	-3917.796988	-3919.299615
**4Pr OH,alpha,S2**	-3917.350466	-3915.943745	-3916.088360	-3917.719302	-3917.863917	-3919.364650
**4Pr DS,alpha,S2**	-3917.345164	-3915.942047	-3916.091062	-3917.722218	-3917.871233	-3919.350859

Table S8: Relative (free) energies (kcal mol^{-1}) of optimized geometries for the C^{alpha} pathway of substrate (2-phenyl butanoic acid or 2-phenylbutyrate; S2) activation by P450 $OleT_{JE}$ model complex as calculated at the UB3LYP level of theory.

System	BS1 (Gas)			BS2 + Solv		
	ΔE_{BS1}	ΔE_{BS1}+ΔZPE	ΔE_{BS1}+ΔG	$\Delta E_{BS2,Solv}$+ ΔZPE	$\Delta E_{BS2,Solv}$+ΔG	$\Delta E_{BS2,Solv,D}$+ΔZPE
**4Re S2**	0.00	0.00	0.00	0.00	0.00	0.00
**4 TS1 alpha,HA,S2**	12.21	9.09	16.70	17.11	24.72	12.08
**4IM1 alpha,S2**	-16.21	-16.22	-11.00	-13.63	-8.40	-22.88
**4Pr OH,alpha,S2**	-54.57	-53.21	-47.71	-55.90	-50.40	-62.31
**4Pr DS,alpha,S2**	-51.24	-52.14	-49.41	-57.73	-54.99	-55.92

Table S9: Imaginary frequency (cm^{-1}) of the transition state structure along the C^{alpha} pathway of substrate (2-phenyl butanoic acid or 2-phenylbutyrate; S2) activation by P450 $OleT_{JE}$ model complex as calculated at the UB3LYP level of theory.

System	Imaginary Frequency (cm^{-1})
**4 TS1 alpha,HA,S2**	i1751.1
**4TS2 reb,alpha,S2**	i255.5

Table S10: Absolute (free) energies (in au) of optimized geometries for the C^{beta} pathway of substrate (2-phenyl butanoic acid or 2-phenylbutyrate; S2) activation by P450 $OleT_{JE}$ model complex as calculated at the UB3LYP level of theory.

System	BS1 (Gas)			BS2 + Solv		
	E_{BS1}	E_{BS1}+ Δ ZPE	E_{BS1}+ ΔG	$E_{BS2,solv}$ + ΔZPE	$E_{BS2,solv}$ + ΔG	$E_{BS2,Solv,D}$
**4 TS1 beta,HA,S2**	-3917.237998	-3915.839318	-3915.984011	-3917.603225	-3917.747918	-3919.233626
**4IM1 beta,S2**	-3917.243933	-3915.842263	-3915.991032	-3917.624343	-3917.773111	-3919.259034
**4 TS2 beta,DS,S2**	-3917.253023	-3915.853885	-3915.995392	-3917.619387	-3917.760894	-3919.246726
**4 Pr beta,DS,S2**	-3917.348966	-3915.946350	-3916.096040	-3917.722386	-3917.872076	-3919.341592
**4 TS2 beta,DC,S2**	-3917.212883	-3915.813121	-3915.961915	-3917.594996	-3917.743790	-3919.224620
**4 Pr beta,DC,S2**	-3917.236538	-3915.835067	-3915.986775	-3917.615976	-3917.767684	-3919.253117

Table S11: Relative (free) energies (kcal mol^{-1}) of optimized geometries for the C^{beta} pathway of substrate (2-phenyl butanoic acid or 2-phenylbutyrate; S2) activation by P450 $OleT_{JE}$ model complex as calculated at the UB3LYP level of theory.

System	BS1 (Gas)			BS2 + Solv		
	ΔE_{BS1}	ΔE_{BS1}+ΔZPE	ΔE_{BS1}+ΔG	$\Delta E_{BS2,Solv}$+ ΔZPE	$\Delta E_{BS2,Solv}$+ΔG	$\Delta E_{BS2,Solv,D}$+ΔZPE
**4 TS1 beta,HA,S2**	16.01	12.32	17.77	16.94	22.39	14.86
**4IM1 beta,S2**	12.28	10.47	13.36	3.69	6.58	0.79
**4 TS2 beta,DS,S2**	6.58	3.18	10.63	6.80	14.25	6.93
**4 Pr beta,DS,S2**	-53.62	-54.84	-52.53	-57.83	-55.52	-50.42
**4 TS2 beta,DC,S2**	31.77	28.76	31.64	22.10	24.98	21.19
**4 Pr beta,DC,S2**	16.92	14.99	16.04	8.94	9.99	4.38

Table S12: Imaginary frequency (cm^{-1}) of the transition state structure along the C^{beta} pathway of substrate (2-phenyl butanoic acid or 2-phenylbutyrate; S2) activation by P450 $OleT_{JE}$ model complex as calculated at the UB3LYP level of theory.

System	Imaginary Frequency (cm^{-1})
**4 TS1 beta,HA,S2**	i1579.2
**4 TS2 beta,DS,S2**	i330.5
**4 TS2 beta,DC,S2**	i291.3

Table S13: Absolute (free) energies (in au) of optimized geometries for the C^{gamma} pathway of substrate (2-phenyl butanoic acid or 2-phenylbutyrate; S2) activation by P450 OleT$_{JE}$ model complex as calculated at the UB3LYP level of theory.

System	BS1 (Gas)			BS2 + Solv		
	E_{BS1}	E_{BS1}+ Δ ZPE	E_{BS1}+ ΔG	$E_{BS2,solv}$ + ΔZPE	$E_{BS2,solv}$ + ΔG	$E_{BS2,Solv,D}$
**4 TS gamma,HA,S2**	-3917.228455	-3915.831381	-3915.976709	-3917.572820	-3917.718149	-3919.204583
**4IM1 gamma,HA,S2**	-3917.236897	-3915.836426	-3915.984758	-3917.612439	-3917.760772	-3919.244989
**4 TS2 gamma,rebound,S2**	-3917.236733	-3915.836137	-3915.981611	-3917.604033	-3917.749507	-3919.234605
**4Pr gamma,OH,S2**	-3917.330842	-3915.923650	-3916.073405	-3917.701976	-3917.851731	-3919.341084
**4 TS2 gamma,DS,S2**	-3917.209908	-3915.814808	-3915.959304	-3917.568507	-3917.713003	-3919.201675
**4Pr gamma,DS,S2**	-3917.319246	-3915.916746	-3916.066287	-3917.694932	-3917.844473	-3919.331547

Table S14: Relative (free) energies (kcal mol^{-1}) of optimized geometries for the C^{gamma} pathway of substrate (2-phenyl butanoic acid or 2-phenylbutyrate; S2) activation by P450 OleT$_{JE}$ model complex as calculated at the UB3LYP level of theory.

System	BS1 (Gas)			BS2 + Solv		
	ΔE_{BS1}	ΔE_{BS1}+ΔZPE	ΔE_{BS1}+ΔG	$\Delta E_{BS2,Solv}$+ ΔZPE	$\Delta E_{BS2,Solv}$+ΔG	$\Delta E_{BS2,Solv,D}$+ΔZPE
**4 TS gamma,HA,S2**	22.00	17.30	22.35	36.02	41.07	32.07
**4IM1 gamma,HA,S2**	16.70	14.14	17.30	11.16	14.32	8.85
**4 TS2 gamma,rebound,S2**	16.80	14.32	19.28	16.43	21.39	15.45
**4Pr gamma,OH,S2**	-42.25	-40.60	-38.33	-45.03	-42.75	-47.23
**4 TS2 gamma,DS,S2**	33.63	27.70	33.27	38.73	44.30	32.66
**4Pr gamma,DS,S2**	-34.98	-36.27	-33.86	-40.61	-38.20	-44.19

Table S15: Imaginary frequency (cm^{-1}) of the transition state structure along the C^{gamma} pathway of substrate (2-phenyl butanoic acid or 2-phenylbutyrate; S2) activation by P450 OleT$_{JE}$ model complex as calculated at the UB3LYP level of theory.

System	Imaginary Frequency (cm^{-1})
**4 TS gamma,HA,S2**	i1420.9
**4 TS2 gamma,DS,S2**	i1629.8

BDE- Model S1

Table S16: Absolute (free) energies (in au) of optimized geometries of substrate (3-phenyl propionic acid or 3-phenylpropionate; S1) and substrate fragments as calculated at the UB3LYP level of theory. Substrate_S1_alpha stands for substrate with a hydrogen atom removed from the C^{α}–H position, which is a radical, while Substrate_S1_beta has the radical on the C^{β} carbon atom.

System	BS1 (Gas)			BS2 + Solv		
	E_{BS1}	E_{BS1}+ ΔZPE	E_{BS1}+ ΔG	$E_{BS2,solv}$ + ΔZPE	$E_{BS2,solv}$ + ΔG	$E_{BS2,Solv,D}$
**Substrate_S1**	-498.7460898	-498.585962	-498.621987	-499.117010	-498.956882	-498.992908
**H atom**	-0.500273	-0.500273	-0.510927	-0.502279	-0.502279	-0.512933
**Substrate_S1_alpha**	-498.0897466	-497.943491	-497.979909	-498.459970	-498.313714	-498.350132
**Substrate_S1_beta**	-498.1041851	-497.957793	-497.994336	-498.472246	-498.325854	-498.362397

Table S17: Bond Dissociation Energy (BDE) and bond dissociation free energy (BDFE) values (kcal mol^{-1}) of optimized geometries of substrate (3-phenyl propionic acid or 3-phenylpropionate; S1) by removal of a hydrogen atom from specific C–H bonds in the substrate and as calculated at the UB3LYP level of theory.

System	BDE_{BS1}	BDE_{BS2} + ΔZPE	$BDFE_{BS2}$
**CH,S1,alpha**	97.9	88.4	81.5
**CH,S1,beta**	88.9	80.8	73.8

BDE- Model S2

Table S18: Absolute (free) energies (in au) of optimized geometries of substrate (2-phenyl butanoic acid or 2-phenylbutyrate; S2) and substrate fragments as calculated at the UB3LYP level of theory. Substrate_S2_alpha stands for substrate with a hydrogen atom removed from the C^{α}–H position, which is a radical.

System	BS1 (Gas)			BS2 + Solv		
	E_{BS1}	E_{BS1}+ ΔZPE	E_{BS1}+ ΔG	$E_{BS2,solv}$ + ΔZPE	$E_{BS2,solv}$ + ΔG	$E_{BS2,Solv,D}$
**Substrate_S2**	-538.0495606	-537.860968	-537.898569	-538.4363631	-538.2477701	-538.2853721
**H atom**	-0.500273	-0.500273	-0.510927	-0.50227909	-0.502279	-0.512933
**Substrate_S2_alpha**	-537.4147507	-537.239307	-537.27731	-537.7966863	-537.6212433	-537.6592453
**Substrate_S2_beta**	-537.3882572	-537.214491	-537.253159	-537.7742641	-537.6004981	-537.6391661
**Substrate_S2**	-537.3788463	-537.205609	-537.24415	-537.7661333	-537.5928963	-537.6314363

Table S19: Bond Dissociation Energy (BDE) and bond dissociation free energy (BDFE) values (kcal mol^{-1}) of optimized geometries of substrate (2-phenyl butanoic acid or 2-phenylbutyrate; S2) by removal of a hydrogen atom from specific C–H bonds in the substrate and as calculated at the UB3LYP level of theory.

System	BDE_{BS1}	BDE_{BS2} + ΔZPE	$BDFE_{BS2}$
**CH,S2,alpha**	84.4	78.0	71.0
**CH,S2,beta**	101.0	91.0	83.6
**CH,S2,gamma**	107.0	95.8	88.5

Model-S1- Charges and Spin state

Table S20: Group charges of optimized geometries for the C^{alpha} pathway of substrate (3-phenyl propionic acid or 3-phenylpropionate; S1) activation by a P450 $OleT_{JE}$ model complex as calculated at the UB3LYP level of theory.

System		Fe	O	Substrate	Amino Acids	Total
4Re S1	Gas	0.5	-0.4	-0.6	0.5	0.0
4TS1 HA,alpha,S1	Gas	0.5	-0.5	-0.4	0.5	0.0
4IM1 alpha,S1	Gas	0.4	-0.6	-0.3	0.5	0.0
4TS2 reb,alpha,S1	Gas	0.5	-0.6	-0.1	0.3	0.0
4Pr OH,alpha,S1	Gas	0.6	-0.6	0.1	0.0	0.0
4TS2 DS,alpha,S1	Gas	0.6	-0.7	0.0	0.2	0.0
4Pr DS,alpha,S1	Gas	0.4	-0.7	0.2	0.1	0.0

Table S21: Group spin densities of optimized geometries for the C^{alpha} pathway of substrate (3-phenyl propionic acid or 3-phenylpropionate; S1) activation by a P450 $OleT_{JE}$ model complex as calculated at the UB3LYP level of theory.

System		Fe	O	Substrate	Amino Acids	Total
4Re S1	Gas	1.1	0.9	0.0	1.0	3.0
4TS1 HA,alpha,S1	Gas	0.9	0.6	0.5	0.9	3.0
4IM1 alpha,S1	Gas	0.9	0.2	1.0	1.0	3.0
4TS2 reb,alpha,S1	Gas	2.2	0.0	0.8	0.1	3.0
4Pr OH,alpha,S1	Gas	2.6	0.0	0.0	0.4	3.0
4TS2 DS,alpha,S1	Gas	3.0	0.0	0.0	0.0	3.0
4Pr DS,alpha,S1	Gas	1.1	0.0	2.0	-0.1	3.0

Table S22: Group charges of optimized geometries for the C^{beta} pathway of substrate (3-phenyl propionic acid or 3-phenylpropionate; S1) activation by a P450 $OleT_{JE}$ model complex as calculated at the UB3LYP level of theory.

System		Fe	O	Substrate	Amino Acids	Total
4TS1 HA,beta,S1	Gas	0.5	-0.5	-0.3	0.4	0.0
4IM1 beta,S1	Gas	0.5	-0.6	-0.3	0.4	0.0
4TS2 reb,beta,S1	Gas	0.5	-0.6	-0.1	0.2	0.0
4 Pr OH,beta,S1	Gas	0.5	-0.6	0.0	0.1	0.0
4TS2 DS,beta,S1	Gas	0.5	-0.6	-0.1	0.2	0.0
4 Pr DS,beta,S1	Gas	0.6	-0.7	0.2	0.0	0.0
4TS2 DC,beta,S1	Gas	0.5	-0.6	-0.3	0.4	0.0
4 Pr DC,beta,S1	Gas	0.5	-0.6	-0.3	0.4	0.0

Table S23: Group spin densities of optimized geometries for the C^{beta} pathway of substrate (3-phenyl propionic acid or 3-phenylpropionate; S1) activation by a P450 $OleT_{JE}$ model complex as calculated at the UB3LYP level of theory.

System		Fe	O	Substrate	Amino Acids	Total
4TS1 HA,beta,S1	Gas	1.3	0.7	0.5	0.5	3.0
4IM1 beta,S1	Gas	1.9	0.3	1.0	-0.1	3.0
4TS2 reb,beta,S1	Gas	2.4	0.0	0.8	-0.1	3.0
4 Pr OH,beta,S1	Gas	2.5	0.0	0.0	0.5	3.0
4TS2 DS,beta,S1	Gas	2.6	0.1	0.7	-0.3	3.0
4 Pr DS,beta,S1	Gas	2.6	0.0	0.0	0.4	3.0
4TS2 DC,beta,S1	Gas	1.9	0.3	0.9	-0.1	3.0
4 Pr DC,beta,S1	Gas	1.9	0.3	0.9	-0.1	3.0

Model-S2- Charges and Spin state

Table S24: Group charges of optimized geometries for the C^{alpha} pathway of substrate (2-phenyl butanoic acid or 2-phenylbutyrate; S2) activation by a P450 $OleT_{JE}$ model complex as calculated at the UB3LYP level of theory.

System		Fe	O	Substrate	Amino Acids	Total
4Re S2	Gas	0.49	-0.34	-0.65	0.99	0.00
4 TS1 alpha,HA,S2	Gas	0.49	-0.50	-0.39	0.40	0.00
4IM1 alpha,S2	Gas	0.46	-0.65	-0.22	0.41	0.00
4TS2 reb,alpha,S2	Gas	0.48	-0.61	-0.19	0.32	0.00
4Pr OH,alpha,S2	Gas	0.58	-0.62	0.02	0.02	0.00
4Pr DS,alpha,S2	Gas	0.60	-0.75	0.18	-0.03	0.00

Table S25: Group spin densities of optimized geometries for the C^{alpha} pathway of substrate (2-phenyl butanoic acid or 2-phenylbutyrate; S2) activation by a P450 $OleT_{JE}$ model complex as calculated at the UB3LYP level of theory.

System		Fe	O	Substrate	Amino Acids	Total
4Re S2	Gas	1.06	0.96	0.00	2.04	3.00
4 TS1 alpha,HA,S2	Gas	1.30	0.75	0.45	0.50	3.00
4IM1 alpha,S2	Gas	1.84	0.34	1.00	-0.17	3.00
4TS2 reb,alpha,S2	Gas	1.99	-0.03	0.85	0.20	3.00
4Pr OH,alpha,S2	Gas	2.58	0.01	0.00	0.42	3.00
4Pr DS,alpha,S2	Gas	2.62	0.03	0.00	0.34	3.00

Table S26: Group charges of optimized geometries for the C^{beta} pathway of substrate substrate (2-phenyl butanoic acid or 2-phenylbutyrate; S2) activation by a P450 $OleT_{JE}$ model complex as calculated at the UB3LYP level of theory.

System		Fe	O	Substrate	Amino Acids	Total
4 TS1 beta,HA,S2	Gas	0.46	-0.52	-0.32	0.38	0.00
4IM1 beta,S2	Gas	0.44	-0.60	-0.23	0.39	0.00
4 TS2 beta,DS,S2	Gas	0.54	-0.70	-0.01	0.17	0.00
4 Pr beta,DS,S2	Gas	0.60	-0.76	0.19	-0.03	0.00
4 TS2 beta,DC,S2	Gas	0.45	-0.61	-0.26	0.42	0.00
4 Pr beta,DC,S2	Gas	0.45	-0.61	-0.29	0.45	0.00

Table S27: Group spin densities of optimized geometries for the C^{beta} pathway of substrate (2-phenyl butanoic acid or 2-phenylbutyrate; S2) activation by a P450 $OleT_{JE}$ model complex as calculated at the UB3LYP level of theory.

System		Fe	O	Substrate	Amino Acids	Total
4 TS1 beta,HA,S2	Gas	1.33	0.69	0.54	0.44	3.00
4IM1 beta,S2	Gas	1.82	0.30	0.97	-0.09	3.00
4 TS2 beta,DS,S2	Gas	2.34	0.07	0.70	-0.11	3.00
4 Pr beta,DS,S2	Gas	2.61	0.03	0.00	0.35	3.00
4 TS2 beta,DC,S2	Gas	1.85	0.30	0.94	-0.09	3.00
4 Pr beta,DC,S2	Gas	1.85	0.26	0.92	-0.04	3.00

Table S28: Group charges of optimized geometries for the C^{gamma} pathway of substrate (2-phenyl butanoic acid or 2-phenylbutyrate; S2) activation by a P450 $OleT_{JE}$ model complex as calculated at the UB3LYP level of theory.

System		Fe	O	Substrate	Amino Acids	Total
**4 TS gamma,HA,S2**	**Gas**	0.46	-0.56	-0.35	0.45	0.00
**4IM1 gamma,HA,S2**	**Gas**	0.42	-0.63	-0.28	0.49	0.00
**4 TS2 gamma,rebound,S2**	**Gas**	0.42	-0.63	-0.27	0.48	0.00
**4Pr gamma,OH,S2**	**Gas**	0.53	-0.64	0.01	0.10	0.00
**4 TS2 gamma,DS,S2**	**Gas**	0.38	-0.63	0.01	0.24	0.00
**4Pr gamma,DS,S2**	**Gas**	0.57	-0.68	0.17	-0.05	0.00

Table S29: Group spin densities of optimized geometries for the C^{gamma} pathway of substrate (2-phenyl butanoic acid or 2-phenylbutyrate; S2) activation by a P450 $OleT_{JE}$ model complex as calculated at the UB3LYP level of theory.

System		Fe	O	Substrate	Amino Acids	Total
**4 TS gamma,HA,S2**	**Gas**	0.88	0.59	0.58	0.95	3.00
**4IM1 gamma,HA,S2**	**Gas**	0.87	0.20	0.97	0.97	3.00
**4 TS2 gamma,rebound,S2**	**Gas**	0.87	0.20	0.97	0.97	3.00
**4Pr gamma,OH,S2**	**Gas**	2.49	0.00	0.00	0.51	3.00
**4 TS2 gamma,DS,S2**	**Gas**	1.00	0.31	1.51	0.19	3.00
**4Pr gamma,DS,S2**	**Gas**	2.60	0.02	0.00	0.37	3.00

Cartesian coordinates of optimized geometries:

Model-S1 Data

$^{2}Re_{S1}$

26	-5.416003000	-2.059434000	-3.254485000
16	-6.515756000	-3.365337000	-5.152833000
1	-7.074389000	-2.298042000	-5.831057000
6	5.329831000	3.876489000	1.470870000
6	5.234417000	2.360104000	1.808626000
6	3.805495000	1.762519000	1.779686000
6	3.251764000	1.668977000	0.344067000
6	3.800480000	0.373017000	2.448852000
6	4.903280000	4.770399000	2.620226000
8	3.694326000	5.045723000	2.857607000
1	4.691850000	4.107647000	0.612994000
1	5.855900000	1.812291000	1.085734000
1	5.686150000	2.190223000	2.797021000
1	3.148534000	2.424315000	2.360871000
1	2.209243000	1.334507000	0.339662000
1	3.279701000	2.635569000	-0.173020000
1	3.842798000	0.956592000	-0.247891000
1	2.801653000	-0.076363000	2.420172000
1	4.120479000	0.431824000	3.497410000
1	4.483815000	-0.311098000	1.927497000
7	5.909697000	5.244483000	3.407587000
6	5.697069000	6.111406000	4.568295000
6	6.542426000	7.404595000	4.489550000
6	8.035236000	7.137988000	4.419513000
6	8.766661000	6.836647000	5.583234000
6	10.133845000	6.550955000	5.517044000
6	10.795702000	6.560135000	4.282493000
6	10.082021000	6.860860000	3.117539000
6	8.712728000	7.147625000	3.186885000
1	6.865134000	5.010507000	3.174057000
1	5.943124000	5.563637000	5.487867000
1	6.222348000	7.979901000	3.613062000
1	6.310000000	8.009250000	5.375590000
1	8.262359000	6.835177000	6.546179000
1	10.683076000	6.326777000	6.426126000
1	11.857397000	6.341506000	4.231313000
1	10.589073000	6.879529000	2.157879000
1	8.167098000	7.398046000	2.280957000
6	2.086603000	1.622453000	-3.709733000
6	0.928266000	2.533292000	-3.249330000
6	-0.163463000	2.761723000	-4.308526000
6	1.664177000	0.172517000	-4.004576000
1	2.557805000	2.055504000	-4.606807000
1	0.475849000	2.114861000	-2.339322000
1	1.347234000	3.510242000	-2.963610000

1	0.261829000	3.186909000	-5.229407000
1	-0.664785000	1.822923000	-4.576586000
1	-0.925867000	3.452042000	-3.931783000
1	2.530784000	-0.450626000	-4.260440000
1	0.959255000	0.123436000	-4.843221000
1	1.173958000	-0.273356000	-3.130150000
6	-2.530528000	6.264056000	-0.595589000
6	-1.417567000	5.928182000	0.431012000
6	-0.103107000	5.439650000	-0.204152000
6	0.905361000	4.932917000	0.843161000
7	0.390014000	3.724463000	1.505185000
6	0.356940000	3.513315000	2.824213000
7	1.117432000	4.258330000	3.673832000
7	-0.452748000	2.555526000	3.315223000
6	-2.864551000	5.081302000	-1.496620000
8	-2.321135000	4.975050000	-2.631582000
1	-2.192166000	7.066791000	-1.258101000
1	-1.780310000	5.157222000	1.122224000
1	-1.221735000	6.824872000	1.037383000
1	-0.308296000	4.638190000	-0.922910000
1	0.364105000	6.254214000	-0.774377000
1	1.865434000	4.710455000	0.363126000
1	1.093622000	5.702464000	1.598852000
1	0.002489000	2.954187000	0.885987000
1	1.996355000	4.672123000	3.362423000
1	0.927255000	4.229303000	4.661949000
1	-1.099103000	2.012857000	2.675518000
1	-0.300100000	2.207538000	4.248968000
7	-3.713506000	4.128979000	-1.023040000
6	-3.982641000	2.898831000	-1.811990000
6	-5.130530000	2.215639000	-1.047262000
6	-4.922184000	2.675888000	0.413614000
6	-4.438238000	4.133062000	0.270465000
1	-3.075144000	2.283451000	-1.837342000
1	-6.098504000	2.573992000	-1.423089000
1	-5.110701000	1.127834000	-1.155945000
1	-5.833133000	2.603896000	1.016343000
1	-4.144466000	2.072525000	0.896183000
1	-3.784239000	4.430215000	1.094015000
1	-5.284547000	4.835471000	0.232867000
6	-0.920988000	-3.078980000	-8.691171000
6	-0.983199000	-1.568680000	-8.621158000
6	-2.107956000	-0.869479000	-9.091834000
6	-2.180272000	0.525539000	-8.997723000
6	-1.125737000	1.249014000	-8.427116000
6	0.003405000	0.565736000	-7.956525000
6	0.072824000	-0.829054000	-8.057146000
1	0.102250000	-3.430710000	-8.864022000
1	-1.268969000	-3.531568000	-7.752550000
1	-2.931265000	-1.422852000	-9.534804000

1	-3.056713000	1.045231000	-9.372946000
1	-1.176811000	2.330876000	-8.356218000
1	0.828103000	1.117703000	-7.516956000
1	0.958393000	-1.349839000	-7.701897000
6	-7.954454000	-3.539463000	-1.466262000
6	-6.748867000	-4.221207000	-1.547765000
6	-6.476902000	-5.490491000	-0.906253000
6	-5.202519000	-5.844372000	-1.242677000
6	-4.680166000	-4.791481000	-2.087331000
7	-5.639979000	-3.806190000	-2.265411000
6	-3.407342000	-4.769932000	-2.632623000
6	-2.892477000	-3.755961000	-3.431157000
6	-1.554845000	-3.745455000	-3.982095000
6	-1.426503000	-2.586748000	-4.695305000
6	-2.686102000	-1.883867000	-4.586840000
7	-3.571591000	-2.612624000	-3.811637000
6	-2.954991000	-0.660895000	-5.186800000
6	-4.160036000	0.019366000	-5.105951000
6	-4.428012000	1.293488000	-5.737878000
6	-5.719234000	1.622430000	-5.438518000
6	-6.250149000	0.551883000	-4.620618000
7	-5.284910000	-0.420701000	-4.426247000
6	-7.544485000	0.509353000	-4.116439000
6	-8.067421000	-0.507100000	-3.335717000
6	-9.407678000	-0.530014000	-2.792991000
6	-9.518978000	-1.664497000	-2.041691000
6	-8.250433000	-2.354897000	-2.117801000
7	-7.373774000	-1.644345000	-2.931152000
1	-8.735531000	-3.980887000	-0.856351000
1	-2.756128000	-5.609433000	-2.413253000
1	-2.161861000	-0.204528000	-5.768737000
1	-8.196655000	1.344458000	-4.349700000
8	-1.903344000	1.038794000	1.651340000
6	-1.343311000	0.867983000	0.497444000
8	-0.462812000	1.672668000	0.001484000
6	-1.769679000	-0.325276000	-0.350794000
6	-2.164154000	-1.575978000	0.467186000
1	-2.640096000	-0.024830000	-0.950926000
1	-0.958911000	-0.549272000	-1.051616000
1	-2.881482000	-1.261103000	1.231638000
8	-4.923764000	-1.226398000	-1.900122000
1	-1.553770000	-3.469074000	-9.494996000
1	2.861038000	1.612453000	-2.928638000
1	-4.233535000	3.161201000	-2.843012000
1	-3.420163000	6.620951000	-0.062089000
1	6.361073000	4.117896000	1.183669000
1	4.631567000	6.350676000	4.590063000
1	-10.372500000	-2.014234000	-1.479583000
1	-7.181576000	-6.027988000	-0.288873000
1	-6.272779000	2.501760000	-5.733648000

1	-10.151531000	0.233791000	-2.966845000
1	-4.653256000	-6.728463000	-0.954082000
1	-0.823083000	-4.525716000	-3.831871000
1	-3.709300000	1.844529000	-6.325065000
1	-0.570953000	-2.221910000	-5.243405000
6	-0.986252000	-2.274913000	1.119518000
6	-0.535767000	-1.899746000	2.399035000
6	-0.299820000	-3.303122000	0.448739000
6	0.564151000	-2.534762000	2.987279000
1	-1.052171000	-1.097487000	2.915735000
6	0.800985000	-3.939340000	1.033353000
1	-0.634889000	-3.602802000	-0.541376000
6	1.237391000	-3.557770000	2.307464000
1	0.893401000	-2.235976000	3.978922000
1	1.315542000	-4.732414000	0.497728000
1	2.088596000	-4.052841000	2.765655000
1	-2.682350000	-2.272239000	-0.202293000

$^4Re_{S1}$

26	-4.652674000	-2.578096000	-2.555227000
16	-5.846657000	-4.460197000	-3.837204000
1	-7.031286000	-3.797980000	-4.101497000
6	6.348896000	3.828818000	0.744805000
6	6.026631000	2.509681000	1.500362000
6	4.538638000	2.081480000	1.473249000
6	4.086467000	1.651148000	0.063745000
6	4.296645000	0.942788000	2.484320000
6	5.800493000	5.069945000	1.427459000
8	4.745566000	5.642556000	1.043778000
1	5.924507000	3.799989000	-0.261195000
1	6.632125000	1.706878000	1.055920000
1	6.359737000	2.606482000	2.544929000
1	3.929650000	2.943882000	1.781683000
1	3.018003000	1.411844000	0.047547000
1	4.252003000	2.439270000	-0.680066000
1	4.642366000	0.760407000	-0.260029000
1	3.246696000	0.630990000	2.480915000
1	4.559859000	1.250207000	3.505413000
1	4.903658000	0.062849000	2.232397000
7	6.523235000	5.530728000	2.493243000
6	6.193599000	6.743734000	3.245163000
6	5.933774000	6.496791000	4.752510000
6	4.615346000	5.822950000	5.081472000
6	3.463626000	6.597641000	5.316264000
6	2.244014000	5.993847000	5.646486000
6	2.155723000	4.597288000	5.743489000
6	3.292769000	3.814868000	5.503731000
6	4.510326000	4.424798000	5.178297000
1	7.376324000	5.045600000	2.734984000
1	5.313925000	7.178913000	2.765642000

1	6.766441000	5.909268000	5.162782000
1	5.972948000	7.474279000	5.250303000
1	3.528391000	7.681181000	5.261651000
1	1.373283000	6.610015000	5.848975000
1	1.218960000	4.128007000	6.029181000
1	3.235448000	2.734029000	5.581702000
1	5.389670000	3.810056000	5.011755000
6	2.953858000	0.249364000	-3.803340000
6	1.912008000	1.380998000	-3.673752000
6	0.787835000	1.327993000	-4.723374000
6	2.397871000	-1.148709000	-3.481794000
1	3.372952000	0.253511000	-4.822101000
1	1.471904000	1.356496000	-2.667087000
1	2.432545000	2.346767000	-3.764707000
1	1.199085000	1.375112000	-5.742548000
1	0.204212000	0.403099000	-4.640605000
1	0.095399000	2.167228000	-4.594020000
1	3.180108000	-1.915250000	-3.553361000
1	1.591090000	-1.428949000	-4.169237000
1	1.989066000	-1.175482000	-2.464004000
6	-1.576081000	5.943826000	-2.522809000
6	-0.438428000	5.915961000	-1.469770000
6	0.838627000	5.192808000	-1.933798000
6	1.871750000	5.048201000	-0.800784000
7	1.352149000	4.165809000	0.254724000
6	1.359622000	4.432870000	1.564887000
7	2.133063000	5.427922000	2.076427000
7	0.575523000	3.708127000	2.386299000
6	-1.989645000	4.553071000	-2.986256000
8	-1.521027000	4.080619000	-4.063017000
1	-1.236782000	6.473409000	-3.418274000
1	-0.798143000	5.434144000	-0.552317000
1	-0.193280000	6.954005000	-1.200889000
1	0.590256000	4.197799000	-2.321054000
1	1.302587000	5.742086000	-2.764321000
1	2.811156000	4.642660000	-1.194095000
1	2.100302000	6.027740000	-0.368366000
1	0.953450000	3.229373000	-0.039157000
1	3.003700000	5.690201000	1.616710000
1	2.023583000	5.690147000	3.044343000
1	-0.096287000	2.992612000	1.998860000
1	0.756359000	3.726345000	3.378065000
7	-2.827665000	3.829187000	-2.199941000
6	-3.178263000	2.426847000	-2.550692000
6	-4.288576000	2.061674000	-1.549799000
6	-3.984791000	2.949879000	-0.321848000
6	-3.482204000	4.268512000	-0.942722000
1	-2.290068000	1.794614000	-2.434718000
1	-5.270776000	2.316234000	-1.969471000
1	-4.288354000	0.993847000	-1.316319000

1	-4.860025000	3.106407000	0.316699000
1	-3.192036000	2.504147000	0.290132000
1	-2.780172000	4.789265000	-0.287737000
1	-4.315997000	4.950659000	-1.166795000
6	-2.046412000	0.493079000	-9.772310000
6	-2.803741000	1.406933000	-8.831888000
6	-4.154403000	1.720691000	-9.070547000
6	-4.845619000	2.598386000	-8.227533000
6	-4.197981000	3.171406000	-7.123973000
6	-2.856224000	2.862118000	-6.863882000
6	-2.170866000	1.985937000	-7.717036000
1	-1.148177000	0.084838000	-9.296987000
1	-2.666196000	-0.347651000	-10.104825000
1	-4.662712000	1.279568000	-9.924198000
1	-5.884644000	2.838492000	-8.436124000
1	-4.731017000	3.857743000	-6.472820000
1	-2.349664000	3.292866000	-6.004191000
1	-1.127833000	1.756221000	-7.514924000
6	-5.219093000	-4.698448000	0.090054000
6	-4.027294000	-4.847616000	-0.604376000
6	-2.993296000	-5.802763000	-0.268845000
6	-2.005074000	-5.667237000	-1.201719000
6	-2.420706000	-4.620605000	-2.110405000
7	-3.662960000	-4.131037000	-1.732179000
6	-1.677814000	-4.169060000	-3.188433000
6	-2.050020000	-3.142478000	-4.046202000
6	-1.250289000	-2.669423000	-5.156526000
6	-1.947078000	-1.652025000	-5.743189000
6	-3.179995000	-1.502860000	-4.999999000
7	-3.225982000	-2.421414000	-3.966199000
6	-4.170208000	-0.579648000	-5.303805000
6	-5.369610000	-0.444805000	-4.620753000
6	-6.396682000	0.518583000	-4.952178000
6	-7.415731000	0.336930000	-4.060302000
6	-7.018312000	-0.737511000	-3.176186000
7	-5.766745000	-1.208349000	-3.533424000
6	-7.789397000	-1.226230000	-2.128303000
6	-7.421568000	-2.250461000	-1.273834000
6	-8.223959000	-2.742913000	-0.176278000
6	-7.496971000	-3.712696000	0.451596000
6	-6.241208000	-3.832364000	-0.255404000
7	-6.212586000	-2.940807000	-1.323353000
1	-5.370624000	-5.327152000	0.960901000
1	-0.720595000	-4.646322000	-3.369806000
1	-3.991473000	0.088291000	-6.139356000
1	-8.757521000	-0.764334000	-1.965121000
8	-0.975815000	1.748500000	1.377390000
6	-0.450180000	1.168867000	0.347028000
8	0.460938000	1.711145000	-0.391659000
6	-0.957577000	-0.210879000	-0.061959000

6	-1.561864000	-1.049495000	1.086625000
1	-1.735179000	-0.068354000	-0.825976000
1	-0.135020000	-0.742673000	-0.552498000
1	-2.242005000	-0.400122000	1.648090000
8	-3.950317000	-1.447260000	-1.572861000
1	-1.721090000	1.031226000	-10.673188000
1	3.792984000	0.459503000	-3.123531000
1	-3.494097000	2.367759000	-3.595207000
1	-2.433960000	6.492050000	-2.113391000
1	7.438113000	3.926470000	0.640413000
1	7.020639000	7.458752000	3.142876000
1	-7.770234000	-4.300979000	1.315274000
1	-3.037018000	-6.484487000	0.567724000
1	-8.353013000	0.869999000	-3.994168000
1	-9.210076000	-2.378550000	0.072029000
1	-1.077952000	-6.215649000	-1.281812000
1	-0.285742000	-3.069542000	-5.431952000
1	-6.322547000	1.228576000	-5.762400000
1	-1.672148000	-1.045558000	-6.592703000
6	-0.531867000	-1.650565000	2.025394000
6	0.045639000	-0.886311000	3.057921000
6	-0.119035000	-2.986818000	1.878081000
6	1.004846000	-1.443051000	3.911269000
1	-0.266380000	0.146446000	3.172552000
6	0.841403000	-3.545569000	2.729909000
1	-0.561978000	-3.594229000	1.092558000
6	1.408271000	-2.775068000	3.751210000
1	1.433634000	-0.839825000	4.707384000
1	1.143106000	-4.581155000	2.598782000
1	2.150498000	-3.207333000	4.416070000
1	-2.163717000	-1.850276000	0.642364000

^{4}**TS1**$_{HA,alpha,S1}$

26	-3.245746000	-2.343708000	-1.718916000
16	-3.040019000	-4.488283000	-2.947969000
1	-4.323229000	-4.547729000	-3.459773000
6	6.007494000	1.272783000	1.079925000
6	5.123040000	0.403614000	2.018871000
6	3.608161000	0.428401000	1.705058000
6	3.271797000	-0.296777000	0.386618000
6	2.800427000	-0.187761000	2.864065000
6	5.833237000	2.764914000	1.300986000
8	4.996667000	3.449800000	0.652724000
1	5.758247000	1.067451000	0.035805000
1	5.481401000	-0.634047000	1.958131000
1	5.286357000	0.727627000	3.057587000
1	3.296815000	1.476057000	1.600889000
1	2.193099000	-0.248664000	0.201771000
1	3.786373000	0.148825000	-0.473710000
1	3.569387000	-1.353875000	0.445458000

1	1.729558000	-0.139911000	2.641755000
1	2.981504000	0.344402000	3.807094000
1	3.070721000	-1.241803000	3.016216000
7	6.633731000	3.321396000	2.260271000
6	6.655269000	4.749376000	2.587708000
6	6.305056000	5.061482000	4.064650000
6	4.843766000	4.897559000	4.435839000
6	3.976056000	6.005706000	4.406367000
6	2.630746000	5.877384000	4.775115000
6	2.129039000	4.630797000	5.176252000
6	2.979684000	3.517754000	5.198465000
6	4.324306000	3.653494000	4.834479000
1	7.306280000	2.725065000	2.722433000
1	5.951801000	5.237604000	1.909739000
1	6.928754000	4.432113000	4.714470000
1	6.611723000	6.097753000	4.255685000
1	4.363208000	6.979530000	4.118109000
1	1.984629000	6.750063000	4.773667000
1	1.095290000	4.534231000	5.495067000
1	2.599810000	2.550177000	5.509932000
1	4.979717000	2.788952000	4.874919000
6	2.820695000	4.105083000	-7.842512000
6	1.868481000	3.898558000	-6.645040000
6	0.754875000	2.865447000	-6.887838000
6	3.660519000	2.866619000	-8.198629000
1	2.230475000	4.411270000	-8.719253000
1	2.460436000	3.599905000	-5.765670000
1	1.408848000	4.865024000	-6.391411000
1	0.161098000	3.137487000	-7.770828000
1	1.167101000	1.863521000	-7.060696000
1	0.072519000	2.810147000	-6.031626000
1	4.348916000	3.080338000	-9.024982000
1	3.029555000	2.024048000	-8.503494000
1	4.260562000	2.539842000	-7.339217000
6	-0.916077000	4.821096000	-3.201761000
6	0.210518000	4.632951000	-2.154136000
6	1.066461000	3.368827000	-2.349381000
6	2.060612000	3.156932000	-1.189996000
7	1.343753000	2.914417000	0.070445000
6	1.426277000	3.666165000	1.175251000
7	2.481072000	4.496620000	1.388827000
7	0.435162000	3.594802000	2.083486000
6	-1.877797000	3.641480000	-3.270587000
8	-1.748406000	2.767105000	-4.183298000
1	-0.479906000	4.920121000	-4.200228000
1	-0.226559000	4.603046000	-1.149099000
1	0.859186000	5.520680000	-2.185808000
1	0.424637000	2.483816000	-2.436838000
1	1.632815000	3.438195000	-3.288632000
1	2.713821000	2.303831000	-1.403107000

1	2.704254000	4.034188000	-1.077993000
1	0.702114000	2.074479000	0.130406000
1	3.393187000	4.275820000	0.990794000
1	2.458757000	5.127460000	2.175630000
1	-0.436205000	3.041886000	1.873591000
1	0.604455000	3.916063000	3.024352000
7	-2.839431000	3.535128000	-2.320784000
6	-3.754140000	2.360025000	-2.276081000
6	-4.807617000	2.748474000	-1.224978000
6	-4.051405000	3.714490000	-0.285683000
6	-3.139492000	4.509111000	-1.240453000
1	-3.201309000	1.465723000	-1.971847000
1	-5.657099000	3.258321000	-1.698373000
1	-5.185464000	1.865773000	-0.702946000
1	-4.719799000	4.371268000	0.280366000
1	-3.434242000	3.149874000	0.422580000
1	-2.228503000	4.850048000	-0.744508000
1	-3.656545000	5.387015000	-1.655992000
6	-3.340509000	1.284639000	-10.636543000
6	-3.899824000	1.936438000	-9.389991000
6	-5.241153000	2.349930000	-9.330666000
6	-5.763126000	2.931532000	-8.168830000
6	-4.947814000	3.110570000	-7.045398000
6	-3.605179000	2.707250000	-7.087935000
6	-3.092219000	2.126178000	-8.253926000
1	-3.959153000	1.501196000	-11.513922000
1	-2.322365000	1.631403000	-10.847583000
1	-5.878879000	2.216601000	-10.200682000
1	-6.802239000	3.246934000	-8.144290000
1	-5.349843000	3.565026000	-6.144910000
1	-2.965386000	2.841266000	-6.219688000
1	-2.050016000	1.818630000	-8.284082000
6	-3.756265000	-4.400962000	0.985897000
6	-2.439572000	-4.070408000	0.687956000
6	-1.283687000	-4.532581000	1.427190000
6	-0.180701000	-4.025950000	0.799517000
6	-0.654427000	-3.243248000	-0.321566000
7	-2.039317000	-3.277047000	-0.372818000
6	0.157090000	-2.539343000	-1.199459000
6	-0.287418000	-1.768429000	-2.264844000
6	0.569459000	-0.988798000	-3.132838000
6	-0.242903000	-0.356734000	-4.030972000
6	-1.598931000	-0.760566000	-3.725599000
7	-1.607807000	-1.617423000	-2.643602000
6	-2.717501000	-0.353129000	-4.443744000
6	-4.023852000	-0.737415000	-4.184332000
6	-5.172776000	-0.329013000	-4.968560000
6	-6.269097000	-0.907724000	-4.396487000
6	-5.800191000	-1.670151000	-3.257145000
7	-4.426647000	-1.557294000	-3.141360000

6	-6.612280000	-2.408260000	-2.405309000
6	-6.172164000	-3.143714000	-1.316131000
6	-7.027442000	-3.886285000	-0.417321000
6	-6.224683000	-4.434481000	0.542037000
6	-4.866176000	-4.041796000	0.239517000
7	-4.846754000	-3.256968000	-0.911156000
1	-3.926612000	-5.022784000	1.859063000
1	1.226859000	-2.572882000	-1.023822000
1	-2.553756000	0.337184000	-5.261795000
1	-7.678815000	-2.404835000	-2.605700000
8	-1.680304000	1.994973000	1.563651000
6	-1.252062000	0.850812000	1.122410000
8	-0.134490000	0.704849000	0.483134000
6	-2.078152000	-0.372055000	1.361251000
6	-3.239697000	-0.290840000	2.343299000
1	-2.714301000	-0.624372000	0.164221000
1	-1.466271000	-1.270981000	1.442254000
1	-3.761530000	0.657169000	2.183830000
8	-3.420662000	-0.867478000	-0.744272000
1	-3.294179000	0.192370000	-10.529061000
1	3.496502000	4.943269000	-7.618743000
1	-4.172210000	2.176604000	-3.268672000
1	-1.458004000	5.749847000	-2.982163000
1	7.061721000	1.001992000	1.225042000
1	7.656876000	5.144868000	2.373218000
1	-6.510858000	-5.050501000	1.382141000
1	-1.325612000	-5.167027000	2.300523000
1	-7.302331000	-0.835810000	-4.703878000
1	-8.100506000	-3.962822000	-0.516328000
1	0.859140000	-4.158562000	1.060077000
1	1.644232000	-0.932005000	-3.041703000
1	-5.124850000	0.316348000	-5.833403000
1	0.022137000	0.333189000	-4.817186000
6	-2.794577000	-0.394256000	3.796989000
6	-2.375769000	0.746652000	4.506126000
6	-2.780751000	-1.635606000	4.456529000
6	-1.966881000	0.646591000	5.840235000
1	-2.366860000	1.700675000	3.989947000
6	-2.367839000	-1.738047000	5.789882000
1	-3.100402000	-2.524564000	3.918851000
6	-1.961315000	-0.595192000	6.488369000
1	-1.657046000	1.538936000	6.377812000
1	-2.369336000	-2.705663000	6.283778000
1	-1.647052000	-0.670984000	7.525237000
1	-3.944445000	-1.101674000	2.124629000

^{4}IM1$_{alpha,S1}$

26	-4.652674000	-2.578096000	-2.555227000
16	-5.846657000	-4.460197000	-3.837204000
1	-7.031286000	-3.797980000	-4.101497000

6	6.348896000	3.828818000	0.744805000
6	6.026631000	2.509681000	1.500362000
6	4.538638000	2.081480000	1.473249000
6	4.086467000	1.651148000	0.063745000
6	4.296645000	0.942788000	2.484320000
6	5.800493000	5.069945000	1.427459000
8	4.745566000	5.642556000	1.043778000
1	5.924507000	3.799989000	-0.261195000
1	6.632125000	1.706878000	1.055920000
1	6.359737000	2.606482000	2.544929000
1	3.929650000	2.943882000	1.781683000
1	3.018003000	1.411844000	0.047547000
1	4.252003000	2.439270000	-0.680066000
1	4.642366000	0.760407000	-0.260029000
1	3.246696000	0.630990000	2.480915000
1	4.559859000	1.250207000	3.505413000
1	4.903658000	0.062849000	2.232397000
7	6.523235000	5.530728000	2.493243000
6	6.193599000	6.743734000	3.245163000
6	5.933774000	6.496791000	4.752510000
6	4.615346000	5.822950000	5.081472000
6	3.463626000	6.597641000	5.316264000
6	2.244014000	5.993847000	5.646486000
6	2.155723000	4.597288000	5.743489000
6	3.292769000	3.814868000	5.503731000
6	4.510326000	4.424798000	5.178297000
1	7.376324000	5.045600000	2.734984000
1	5.313925000	7.178913000	2.765642000
1	6.766441000	5.909268000	5.162782000
1	5.972948000	7.474279000	5.250303000
1	3.528391000	7.681181000	5.261651000
1	1.373283000	6.610015000	5.848975000
1	1.218960000	4.128007000	6.029181000
1	3.235448000	2.734029000	5.581702000
1	5.389670000	3.810056000	5.011755000
6	2.953858000	0.249364000	-3.803340000
6	1.912008000	1.380998000	-3.673752000
6	0.787835000	1.327993000	-4.723374000
6	2.397871000	-1.148709000	-3.481794000
1	3.372952000	0.253511000	-4.822101000
1	1.471904000	1.356496000	-2.667087000
1	2.432545000	2.346767000	-3.764707000
1	1.199085000	1.375112000	-5.742548000
1	0.204212000	0.403099000	-4.640605000
1	0.095399000	2.167228000	-4.594020000
1	3.180108000	-1.915250000	-3.553361000
1	1.591090000	-1.428949000	-4.169237000
1	1.989066000	-1.175482000	-2.464004000
6	-1.576081000	5.943826000	-2.522809000
6	-0.438428000	5.915961000	-1.469770000

6	0.838627000	5.192808000	-1.933798000
6	1.871750000	5.048201000	-0.800784000
7	1.352149000	4.165809000	0.254724000
6	1.359622000	4.432870000	1.564887000
7	2.133063000	5.427922000	2.076427000
7	0.575523000	3.708127000	2.386299000
6	-1.989645000	4.553071000	-2.986256000
8	-1.521027000	4.080619000	-4.063017000
1	-1.236782000	6.473409000	-3.418274000
1	-0.798143000	5.434144000	-0.552317000
1	-0.193280000	6.954005000	-1.200889000
1	0.590256000	4.197799000	-2.321054000
1	1.302587000	5.742086000	-2.764321000
1	2.811156000	4.642660000	-1.194095000
1	2.100302000	6.027740000	-0.368366000
1	0.953450000	3.229373000	-0.039157000
1	3.003700000	5.690201000	1.616710000
1	2.023583000	5.690147000	3.044343000
1	-0.096287000	2.992612000	1.998860000
1	0.756359000	3.726345000	3.378065000
7	-2.827665000	3.829187000	-2.199941000
6	-3.178263000	2.426847000	-2.550692000
6	-4.288576000	2.061674000	-1.549799000
6	-3.984791000	2.949879000	-0.321848000
6	-3.482204000	4.268512000	-0.942722000
1	-2.290068000	1.794614000	-2.434718000
1	-5.270776000	2.316234000	-1.969471000
1	-4.288354000	0.993847000	-1.316319000
1	-4.860025000	3.106407000	0.316699000
1	-3.192036000	2.504147000	0.290132000
1	-2.780172000	4.789265000	-0.287737000
1	-4.315997000	4.950659000	-1.166795000
6	-2.046412000	0.493079000	-9.772310000
6	-2.803741000	1.406933000	-8.831888000
6	-4.154403000	1.720691000	-9.070547000
6	-4.845619000	2.598386000	-8.227533000
6	-4.197981000	3.171406000	-7.123973000
6	-2.856224000	2.862118000	-6.863882000
6	-2.170866000	1.985937000	-7.717036000
1	-1.148177000	0.084838000	-9.296987000
1	-2.666196000	-0.347651000	-10.104825000
1	-4.662712000	1.279568000	-9.924198000
1	-5.884644000	2.838492000	-8.436124000
1	-4.731017000	3.857743000	-6.472820000
1	-2.349664000	3.292866000	-6.004191000
1	-1.127833000	1.756221000	-7.514924000
6	-5.219093000	-4.698448000	0.090054000
6	-4.027294000	-4.847616000	-0.604376000
6	-2.993296000	-5.802763000	-0.268845000
6	-2.005074000	-5.667237000	-1.201719000

6	-2.420706000	-4.620605000	-2.110405000
7	-3.662960000	-4.131037000	-1.732179000
6	-1.677814000	-4.169060000	-3.188433000
6	-2.050020000	-3.142478000	-4.046202000
6	-1.250289000	-2.669423000	-5.156526000
6	-1.947078000	-1.652025000	-5.743189000
6	-3.179995000	-1.502860000	-4.999999000
7	-3.225982000	-2.421414000	-3.966199000
6	-4.170208000	-0.579648000	-5.303805000
6	-5.369610000	-0.444805000	-4.620753000
6	-6.396682000	0.518583000	-4.952178000
6	-7.415731000	0.336930000	-4.060302000
6	-7.018312000	-0.737511000	-3.176186000
7	-5.766745000	-1.208349000	-3.533424000
6	-7.789397000	-1.226230000	-2.128303000
6	-7.421568000	-2.250461000	-1.273834000
6	-8.223959000	-2.742913000	-0.176278000
6	-7.496971000	-3.712696000	0.451596000
6	-6.241208000	-3.832364000	-0.255404000
7	-6.212586000	-2.940807000	-1.323353000
1	-5.370624000	-5.327152000	0.960901000
1	-0.720595000	-4.646322000	-3.369806000
1	-3.991473000	0.088291000	-6.139356000
1	-8.757521000	-0.764334000	-1.965121000
8	-0.975815000	1.748500000	1.377390000
6	-0.450180000	1.168867000	0.347028000
8	0.460938000	1.711145000	-0.391659000
6	-0.957577000	-0.210879000	-0.061959000
6	-1.561864000	-1.049495000	1.086625000
1	-1.735179000	-0.068354000	-0.825976000
1	-0.135020000	-0.742673000	-0.552498000
1	-2.242005000	-0.400122000	1.648090000
8	-3.950317000	-1.447260000	-1.572861000
1	-1.721090000	1.031226000	-10.673188000
1	3.792984000	0.459503000	-3.123531000
1	-3.494097000	2.367759000	-3.595207000
1	-2.433960000	6.492050000	-2.113391000
1	7.438113000	3.926470000	0.640413000
1	7.020639000	7.458752000	3.142876000
1	-7.770234000	-4.300979000	1.315274000
1	-3.037018000	-6.484487000	0.567724000
1	-8.353013000	0.869999000	-3.994168000
1	-9.210076000	-2.378550000	0.072029000
1	-1.077952000	-6.215649000	-1.281812000
1	-0.285742000	-3.069542000	-5.431952000
1	-6.322547000	1.228576000	-5.762400000
1	-1.672148000	-1.045558000	-6.592703000
6	-0.531867000	-1.650565000	2.025394000
6	0.045639000	-0.886311000	3.057921000
6	-0.119035000	-2.986818000	1.878081000

6	1.004846000	-1.443051000	3.911269000
1	-0.266380000	0.146446000	3.172552000
6	0.841403000	-3.545569000	2.729909000
1	-0.561978000	-3.594229000	1.092558000
6	1.408271000	-2.775068000	3.751210000
1	1.433634000	-0.839825000	4.707384000
1	1.143106000	-4.581155000	2.598782000
1	2.150498000	-3.207333000	4.416070000
1	-2.163717000	-1.850276000	0.642364000

^{4}TS2$_{reb,alpha,S1}$

26	-3.523894000	-2.091571000	-0.725640000
16	-3.168791000	-4.253206000	-1.783753000
1	-4.156845000	-4.160727000	-2.745425000
6	6.201420000	2.046597000	-1.711560000
6	6.661983000	0.891735000	-0.781897000
6	5.546855000	0.168465000	0.010634000
6	4.468876000	-0.432600000	-0.912276000
6	6.172154000	-0.922203000	0.903706000
6	5.503215000	3.172468000	-0.972363000
8	4.257968000	3.164459000	-0.773406000
1	5.500383000	1.669675000	-2.460473000
1	7.184975000	0.154003000	-1.407520000
1	7.406949000	1.280641000	-0.072145000
1	5.057433000	0.903227000	0.666243000
1	3.745157000	-1.013468000	-0.328203000
1	3.915372000	0.348896000	-1.442848000
1	4.916708000	-1.108565000	-1.653176000
1	5.407654000	-1.407635000	1.521039000
1	6.933726000	-0.504702000	1.574476000
1	6.651594000	-1.699584000	0.294280000
7	6.299700000	4.184437000	-0.521294000
6	5.801196000	5.362429000	0.195469000
6	6.355613000	5.493282000	1.636281000
6	5.808545000	4.474786000	2.616832000
6	4.643956000	4.754059000	3.354879000
6	4.122980000	3.819171000	4.259081000
6	4.758054000	2.581726000	4.433942000
6	5.919114000	2.292768000	3.706460000
6	6.440362000	3.233889000	2.810634000
1	7.284584000	4.153305000	-0.746006000
1	4.711818000	5.283132000	0.207867000
1	7.451916000	5.432336000	1.601741000
1	6.113131000	6.504941000	1.985697000
1	4.150178000	5.714278000	3.232339000
1	3.238193000	4.065728000	4.838723000
1	4.363685000	1.859638000	5.141659000
1	6.423838000	1.341955000	3.844538000
1	7.351611000	3.007801000	2.264748000
6	3.522191000	5.694030000	-5.162029000

6	1.991114000	5.746364000	-4.973133000
6	1.210091000	4.700435000	-5.786912000
6	4.172650000	4.400907000	-4.639593000
1	3.757685000	5.816272000	-6.229871000
1	1.762406000	5.624555000	-3.902792000
1	1.634819000	6.749241000	-5.248808000
1	1.383689000	4.834911000	-6.862566000
1	1.515945000	3.678787000	-5.527867000
1	0.133322000	4.778435000	-5.600482000
1	5.264063000	4.436806000	-4.748958000
1	3.815494000	3.521956000	-5.188254000
1	3.941774000	4.246084000	-3.577143000
6	-1.988274000	5.491680000	-2.495815000
6	-1.127243000	4.732675000	-1.452658000
6	-0.275173000	3.598476000	-2.053313000
6	0.843168000	3.124712000	-1.115973000
7	0.300032000	2.666603000	0.172775000
6	1.012898000	2.617192000	1.303767000
7	2.351084000	2.835481000	1.295972000
7	0.388438000	2.365282000	2.470927000
6	-2.721739000	4.618373000	-3.505386000
8	-2.260232000	4.477989000	-4.676573000
1	-1.338565000	6.133983000	-3.097987000
1	-1.758085000	4.319255000	-0.660140000
1	-0.469701000	5.472002000	-0.971923000
1	-0.920499000	2.749822000	-2.311599000
1	0.191213000	3.931562000	-2.988973000
1	1.412690000	2.318424000	-1.597289000
1	1.543429000	3.950119000	-0.934264000
1	-0.703885000	2.361816000	0.211658000
1	2.902330000	2.910078000	0.443854000
1	2.844899000	2.970740000	2.165229000
1	-0.659863000	2.322028000	2.529790000
1	0.930753000	2.150756000	3.291903000
7	-3.884518000	4.022246000	-3.132933000
6	-4.694184000	3.259649000	-4.113737000
6	-6.049045000	3.093635000	-3.406513000
6	-5.662839000	3.022984000	-1.913061000
6	-4.493184000	4.020965000	-1.777278000
1	-4.223490000	2.291197000	-4.319669000
1	-6.687461000	3.965654000	-3.597027000
1	-6.580736000	2.199644000	-3.742549000
1	-6.492812000	3.266896000	-1.243110000
1	-5.313928000	2.013052000	-1.668593000
1	-3.774442000	3.687835000	-1.025388000
1	-4.850155000	5.027327000	-1.516590000
6	0.172304000	-0.872272000	-7.745697000
6	-1.090210000	-0.038774000	-7.691932000
6	-2.270961000	-0.480158000	-8.314164000
6	-3.447227000	0.274434000	-8.238118000

6	-3.461890000	1.485179000	-7.533602000
6	-2.294263000	1.942394000	-6.906720000
6	-1.120282000	1.182603000	-6.994647000
1	1.068716000	-0.244186000	-7.697462000
1	0.219911000	-1.576094000	-6.903146000
1	-2.266325000	-1.418844000	-8.861806000
1	-4.346687000	-0.079599000	-8.733635000
1	-4.371846000	2.075955000	-7.483257000
1	-2.296545000	2.873849000	-6.347016000
1	-0.212639000	1.550646000	-6.522163000
6	-4.131501000	-3.863701000	2.144373000
6	-2.816218000	-3.533513000	1.860548000
6	-1.690795000	-3.849150000	2.712952000
6	-0.580854000	-3.301233000	2.135056000
6	-1.011946000	-2.649497000	0.917068000
7	-2.377027000	-2.814554000	0.761976000
6	-0.175601000	-1.966222000	0.047027000
6	-0.582028000	-1.366493000	-1.134219000
6	0.309400000	-0.752213000	-2.092969000
6	-0.449231000	-0.368918000	-3.164262000
6	-1.816848000	-0.735924000	-2.871831000
7	-1.882265000	-1.326135000	-1.616651000
6	-2.880947000	-0.548628000	-3.739014000
6	-4.193309000	-0.904659000	-3.464072000
6	-5.311382000	-0.651737000	-4.344846000
6	-6.439615000	-1.073379000	-3.694598000
6	-6.024820000	-1.602318000	-2.414841000
7	-4.645279000	-1.504846000	-2.297966000
6	-6.884139000	-2.134627000	-1.465185000
6	-6.484565000	-2.698523000	-0.263811000
6	-7.375654000	-3.332934000	0.686090000
6	-6.598580000	-3.837369000	1.689390000
6	-5.224465000	-3.509492000	1.370707000
7	-5.179406000	-2.806561000	0.180939000
1	-4.322145000	-4.414145000	3.057943000
1	0.879615000	-1.922899000	0.294598000
1	-2.674121000	-0.082643000	-4.695903000
1	-7.944395000	-2.136465000	-1.693779000
8	-2.301877000	2.185462000	2.586335000
6	-2.900241000	1.877091000	1.485497000
8	-2.337835000	1.941976000	0.304533000
6	-4.299121000	1.416645000	1.531781000
6	-5.045962000	1.261356000	2.826718000
1	-3.121502000	0.223819000	-0.131900000
1	-4.875154000	1.524176000	0.622880000
1	-4.802010000	2.152210000	3.430108000
8	-3.779665000	-0.447221000	0.191747000
1	0.223062000	-1.464813000	-8.665602000
1	3.973629000	6.554840000	-4.646968000
1	-4.747184000	3.813870000	-5.054649000

**1**	**-2.700190000**	**6.139339000**	**-1.967723000**
**1**	**7.074402000**	**2.435879000**	**-2.251258000**
**1**	**6.069465000**	**6.260880000**	**-0.374720000**
**1**	**-6.908061000**	**-4.382840000**	**2.568731000**
**1**	**-1.762119000**	**-4.405732000**	**3.635762000**
**1**	**-7.462907000**	**-1.043774000**	**-4.040522000**
**1**	**-8.449103000**	**-3.388303000**	**0.575894000**
**1**	**0.440467000**	**-3.326289000**	**2.486907000**
**1**	**1.377599000**	**-0.657348000**	**-1.958993000**
**1**	**-5.220996000**	**-0.212201000**	**-5.327667000**
**1**	**-0.133066000**	**0.108061000**	**-4.080401000**
**6**	**-4.781887000**	**0.040679000**	**3.711599000**
**6**	**-3.519137000**	**-0.200820000**	**4.280808000**
**6**	**-5.847096000**	**-0.808660000**	**4.050755000**
**6**	**-3.334659000**	**-1.265081000**	**5.169072000**
**1**	**-2.699024000**	**0.460798000**	**4.026389000**
**6**	**-5.664213000**	**-1.869912000**	**4.945792000**
**1**	**-6.827341000**	**-0.636116000**	**3.615218000**
**6**	**-4.406023000**	**-2.100545000**	**5.511282000**
**1**	**-2.353302000**	**-1.437400000**	**5.601858000**
**1**	**-6.504517000**	**-2.508851000**	**5.202269000**
**1**	**-4.262416000**	**-2.916899000**	**6.213502000**
**1**	**-6.119869000**	**1.296423000**	**2.615183000**

$^{4}Pr_{OH,alpha,S1}$

26	-2.445461000	-2.715986000	-1.623812000
16	-2.571049000	-4.431842000	-3.435923000
1	-2.569827000	-3.548292000	-4.499792000
6	5.494413000	1.239860000	1.078394000
6	4.898098000	0.172765000	2.030627000
6	3.360178000	0.234323000	2.202784000
6	2.615649000	-0.184458000	0.919994000
6	2.924046000	-0.648660000	3.388750000
6	5.359994000	2.672686000	1.571409000
8	4.704188000	3.540478000	0.933439000
1	5.006103000	1.198236000	0.102704000
1	5.171846000	-0.820086000	1.646479000
1	5.376211000	0.256051000	3.019007000
1	3.083598000	1.274993000	2.434778000
1	1.535577000	-0.041109000	1.024872000
1	2.932330000	0.400351000	0.049279000
1	2.796115000	-1.244790000	0.698551000
1	1.838564000	-0.611022000	3.528894000
1	3.401331000	-0.330324000	4.325477000
1	3.200948000	-1.696843000	3.212887000
7	6.015837000	2.974918000	2.729919000
6	6.097753000	4.321805000	3.302887000
6	5.515114000	4.434294000	4.734196000
6	4.000806000	4.422419000	4.825409000
6	3.283963000	5.633981000	4.824038000

6	1.888563000	5.644999000	4.943896000
6	1.185474000	4.437222000	5.061273000
6	1.884849000	3.223077000	5.046614000
6	3.280590000	3.219968000	4.934708000
1	6.542073000	2.240928000	3.183998000
1	5.572094000	4.987985000	2.615384000
1	5.933531000	3.626151000	5.349652000
1	5.892652000	5.371956000	5.161773000
1	3.824811000	6.574393000	4.756817000
1	1.356337000	6.591066000	4.973306000
1	0.106977000	4.442892000	5.192212000
1	1.344574000	2.285385000	5.128927000
1	3.815861000	2.275586000	4.945958000
6	3.068922000	3.612886000	-8.350064000
6	2.050811000	3.755656000	-7.198558000
6	0.859258000	2.785893000	-7.280932000
6	3.818841000	2.270031000	-8.361813000
1	2.544543000	3.747257000	-9.307965000
1	2.574271000	3.614256000	-6.239632000
1	1.671279000	4.788206000	-7.191335000
1	0.322793000	2.909239000	-8.230998000
1	1.185219000	1.740453000	-7.220065000
1	0.146590000	2.961844000	-6.467111000
1	4.555089000	2.235629000	-9.173691000
1	3.134117000	1.425838000	-8.500222000
1	4.355333000	2.113073000	-7.416762000
6	-0.441092000	5.232819000	-3.648604000
6	0.528168000	4.953047000	-2.471115000
6	1.228465000	3.584805000	-2.533989000
6	2.004143000	3.265190000	-1.240529000
7	1.078442000	3.104621000	-0.109237000
6	1.060958000	3.832163000	1.015205000
7	2.136132000	4.557624000	1.409843000
7	-0.061985000	3.847406000	1.760866000
6	-1.588321000	4.235763000	-3.741842000
8	-1.517599000	3.243953000	-4.530667000
1	0.102931000	5.167713000	-4.595801000
1	-0.019209000	5.021428000	-1.522961000
1	1.285001000	5.750285000	-2.452931000
1	0.495447000	2.789668000	-2.715259000
1	1.929809000	3.559884000	-3.379641000
1	2.575814000	2.339178000	-1.368471000
1	2.722454000	4.058145000	-1.015960000
1	0.355756000	2.345750000	-0.172404000
1	3.079481000	4.297592000	1.119883000
1	2.049524000	5.171153000	2.205896000
1	-0.943999000	3.410409000	1.413095000
1	-0.004899000	4.121946000	2.729514000
7	-2.673656000	4.424528000	-2.947988000
6	-3.814482000	3.470888000	-2.979119000

6	-4.868707000	4.127707000	-2.070298000
6	-4.024602000	4.953881000	-1.074961000
6	-2.885949000	5.506315000	-1.953946000
1	-3.487347000	2.500544000	-2.592062000
1	-5.523156000	4.784976000	-2.656848000
1	-5.497469000	3.384000000	-1.573339000
1	-4.593439000	5.752105000	-0.588374000
1	-3.608779000	4.296123000	-0.302724000
1	-1.979007000	5.702030000	-1.378812000
1	-3.183675000	6.437170000	-2.458739000
6	-5.890282000	-0.417691000	-7.963829000
6	-5.548845000	1.045995000	-7.784602000
6	-6.433346000	2.056483000	-8.196300000
6	-6.112534000	3.408871000	-8.023076000
6	-4.898969000	3.772843000	-7.429970000
6	-4.003898000	2.777290000	-7.012879000
6	-4.329840000	1.427970000	-7.193132000
1	-5.898224000	-0.945675000	-7.001851000
1	-6.875041000	-0.544641000	-8.424857000
1	-7.378866000	1.780855000	-8.655830000
1	-6.810309000	4.174000000	-8.351079000
1	-4.649022000	4.820985000	-7.295445000
1	-3.063710000	3.044441000	-6.539977000
1	-3.630699000	0.660050000	-6.872309000
6	-2.325330000	-5.073631000	0.878247000
6	-1.120062000	-4.656886000	0.332661000
6	0.173235000	-5.198975000	0.686606000
6	1.101548000	-4.569075000	-0.094395000
6	0.388846000	-3.624542000	-0.925792000
7	-0.970565000	-3.691915000	-0.652302000
6	0.989767000	-2.756095000	-1.824997000
6	0.318803000	-1.796965000	-2.569468000
6	0.954748000	-0.818834000	-3.423955000
6	-0.032316000	-0.016465000	-3.926720000
6	-1.286137000	-0.510377000	-3.402726000
7	-1.055440000	-1.595922000	-2.568513000
6	-2.528326000	0.007182000	-3.738145000
6	-3.741868000	-0.503641000	-3.296553000
6	-5.039625000	-0.019895000	-3.714063000
6	-5.978613000	-0.786194000	-3.078631000
6	-5.268540000	-1.738371000	-2.255837000
7	-3.899320000	-1.557691000	-2.404734000
6	-5.872392000	-2.659905000	-1.413372000
6	-5.193696000	-3.531544000	-0.575991000
6	-5.827976000	-4.441134000	0.352363000
6	-4.830638000	-5.100583000	1.014679000
6	-3.574584000	-4.611183000	0.490484000
7	-3.813856000	-3.646985000	-0.476957000
1	-2.290788000	-5.842271000	1.642889000
1	2.067615000	-2.811680000	-1.932862000

1	-2.541414000	0.874432000	-4.388843000
1	-6.956583000	-2.685623000	-1.392691000
8	-2.303311000	2.501690000	0.892985000
6	-1.903239000	1.358951000	0.472967000
8	-0.723650000	1.098872000	0.011677000
6	-2.869780000	0.164780000	0.520595000
6	-3.556676000	0.005977000	1.892227000
1	-1.210424000	-0.732593000	-0.043137000
1	-3.646478000	0.315281000	-0.240107000
1	-4.221032000	0.866869000	2.020709000
8	-2.127522000	-1.040099000	0.174826000
1	-5.155012000	-0.925162000	-8.600986000
1	3.801084000	4.430791000	-8.282501000
1	-4.152095000	3.327108000	-4.008901000
1	-0.829156000	6.255100000	-3.558984000
1	6.560702000	1.025736000	0.917948000
1	7.151943000	4.627811000	3.327267000
1	-4.921963000	-5.856548000	1.781229000
1	0.332877000	-5.968693000	1.427990000
1	-7.054784000	-0.720312000	-3.149677000
1	-6.896383000	-4.548080000	0.472133000
1	2.171380000	-4.719094000	-0.116165000
1	2.019391000	-0.760862000	-3.599655000
1	-5.194292000	0.784884000	-4.417898000
1	0.050048000	0.835094000	-4.586153000
6	-2.614714000	-0.091754000	3.075555000
6	-2.439001000	1.003537000	3.939866000
6	-1.904506000	-1.277218000	3.341410000
6	-1.589708000	0.915999000	5.049338000
1	-2.973225000	1.925788000	3.733250000
6	-1.049191000	-1.364108000	4.445736000
1	-2.024919000	-2.124931000	2.676085000
6	-0.891541000	-0.270240000	5.307763000
1	-1.488556000	1.764399000	5.721519000
1	-0.515675000	-2.289881000	4.640616000
1	-0.245025000	-0.348080000	6.177276000
1	-4.181847000	-0.892911000	1.833626000

$^{4}TS2_{DS,alpha,S1}$

26	-4.060472000	-2.627071000	-1.773193000
16	-3.478603000	-3.946116000	-3.660892000
1	-2.453542000	-4.668454000	-3.080456000
6	6.706428000	2.000410000	1.385100000
6	5.851847000	1.089267000	2.312970000
6	4.392691000	0.867405000	1.848458000
6	4.309576000	-0.022086000	0.592047000
6	3.543774000	0.260073000	2.982721000
6	6.329444000	3.468006000	1.473457000
8	5.427430000	3.978651000	0.753548000
1	6.580692000	1.692828000	0.343445000

1	6.354332000	0.114318000	2.387198000
1	5.853114000	1.517305000	3.326442000
1	3.963689000	1.845635000	1.596442000
1	3.263806000	-0.149985000	0.292197000
1	4.855755000	0.406956000	-0.256950000
1	4.730097000	-1.016982000	0.794078000
1	2.508936000	0.131433000	2.647473000
1	3.542421000	0.905507000	3.871087000
1	3.930967000	-0.723652000	3.281656000
7	7.017716000	4.207104000	2.393614000
6	6.841511000	5.648965000	2.589160000
6	6.369466000	6.039989000	4.012121000
6	4.920755000	5.720614000	4.328142000
6	3.915754000	6.678163000	4.093003000
6	2.577286000	6.410682000	4.408227000
6	2.220616000	5.171679000	4.960605000
6	3.210143000	4.206809000	5.189973000
6	4.547073000	4.482433000	4.879087000
1	7.749713000	3.749806000	2.919474000
1	6.124095000	5.982758000	1.836169000
1	7.027391000	5.557055000	4.747386000
1	6.531613000	7.120330000	4.118096000
1	4.187746000	7.648509000	3.685799000
1	1.821943000	7.174564000	4.249496000
1	1.189592000	4.973543000	5.239118000
1	2.944160000	3.248433000	5.624343000
1	5.309028000	3.735380000	5.080190000
6	3.415358000	2.153878000	-7.718425000
6	2.443579000	2.486203000	-6.565743000
6	1.225703000	3.327419000	-6.984262000
6	2.818648000	1.230978000	-8.794385000
1	3.750817000	3.092381000	-8.185749000
1	2.099483000	1.548102000	-6.104855000
1	2.998774000	3.025734000	-5.783192000
1	1.545535000	4.273155000	-7.443209000
1	0.604556000	2.799479000	-7.717690000
1	0.590985000	3.562443000	-6.122526000
1	3.559173000	0.995452000	-9.568237000
1	1.957019000	1.692000000	-9.290330000
1	2.481811000	0.283729000	-8.353947000
6	0.196741000	5.644747000	-3.005310000
6	1.249671000	5.095922000	-2.007808000
6	1.812303000	3.708653000	-2.364812000
6	2.698921000	3.114625000	-1.248597000
7	1.932581000	2.862833000	-0.016054000
6	1.825027000	3.692717000	1.030403000
7	2.748711000	4.653803000	1.277077000
7	0.761857000	3.560243000	1.848994000
6	-0.995789000	4.718575000	-3.207948000
8	-1.034755000	3.939867000	-4.206734000

1	0.652943000	5.770205000	-3.991676000
1	0.804529000	5.048906000	-1.007507000
1	2.073227000	5.821078000	-1.945230000
1	0.989712000	3.013501000	-2.571627000
1	2.405908000	3.769327000	-3.287738000
1	3.104853000	2.151407000	-1.573548000
1	3.555900000	3.759853000	-1.038437000
1	1.389470000	1.966635000	0.056929000
1	3.710893000	4.546593000	0.955510000
1	2.580786000	5.323643000	2.012817000
1	-0.027983000	2.930267000	1.576570000
1	0.794065000	3.954128000	2.776761000
7	-1.994284000	4.724299000	-2.286082000
6	-3.156766000	3.803152000	-2.420611000
6	-4.093073000	4.223530000	-1.273949000
6	-3.127139000	4.785744000	-0.208306000
6	-2.077749000	5.538518000	-1.049145000
1	-2.812987000	2.770297000	-2.307941000
1	-4.788321000	5.003617000	-1.609445000
1	-4.685296000	3.382627000	-0.903154000
1	-3.619709000	5.438282000	0.519079000
1	-2.646668000	3.960310000	0.330850000
1	-1.111561000	5.599310000	-0.544490000
1	-2.411429000	6.560496000	-1.282550000
6	-4.207479000	-0.063048000	-8.412458000
6	-4.353816000	1.331808000	-7.844243000
6	-5.559051000	2.044512000	-7.969424000
6	-5.700617000	3.322790000	-7.413910000
6	-4.636037000	3.909997000	-6.720609000
6	-3.425599000	3.215299000	-6.588109000
6	-3.290736000	1.939749000	-7.150553000
1	-4.989448000	-0.280044000	-9.147424000
1	-3.235927000	-0.196903000	-8.901660000
1	-6.388356000	1.595865000	-8.510104000
1	-6.639137000	3.857949000	-7.526882000
1	-4.744418000	4.902117000	-6.292483000
1	-2.594987000	3.649563000	-6.039771000
1	-2.348747000	1.407358000	-7.047324000
6	-4.025505000	-5.460987000	0.193052000
6	-2.846750000	-4.733249000	0.100250000
6	-1.602352000	-5.047085000	0.782073000
6	-0.722891000	-4.033521000	0.513754000
6	-1.408643000	-3.083183000	-0.351752000
7	-2.672704000	-3.535605000	-0.598430000
6	-0.863735000	-1.841797000	-0.786642000
6	-1.403755000	-1.011189000	-1.807336000
6	-0.726291000	0.103804000	-2.465795000
6	-1.597462000	0.617662000	-3.388771000
6	-2.827626000	-0.164363000	-3.286973000
7	-2.648600000	-1.150080000	-2.326013000

6	-4.016886000	0.045648000	-3.966310000
6	-5.230636000	-0.656530000	-3.805707000
6	-6.453606000	-0.365065000	-4.528838000
6	-7.401551000	-1.258633000	-4.101713000
6	-6.774552000	-2.118189000	-3.116949000
7	-5.456560000	-1.711930000	-2.946690000
6	-7.391153000	-3.200757000	-2.480930000
6	-6.815033000	-4.115164000	-1.596847000
6	-7.453478000	-5.279846000	-1.004952000
6	-6.499421000	-5.924178000	-0.260353000
6	-5.270503000	-5.155591000	-0.386394000
7	-5.499171000	-4.070289000	-1.188221000
1	-3.984289000	-6.361682000	0.798874000
1	0.179785000	-1.665312000	-0.550252000
1	-4.028700000	0.849100000	-4.696534000
1	-8.434435000	-3.366015000	-2.735132000
8	-1.145886000	1.750623000	1.110740000
6	-0.560179000	0.624496000	0.881982000
8	0.660477000	0.506519000	0.464338000
6	-1.311716000	-0.636063000	1.222616000
6	-2.705662000	-0.616901000	1.580006000
1	-5.095451000	-0.761543000	-0.499104000
1	-0.682760000	-1.424991000	1.622674000
1	-3.048988000	0.426026000	1.639773000
8	-4.492717000	-1.491125000	-0.240007000
1	-4.277358000	-0.821940000	-7.621743000
1	4.315198000	1.679092000	-7.300322000
1	-3.603087000	3.902228000	-3.413614000
1	-0.136899000	6.633192000	-2.664300000
1	7.767952000	1.878041000	1.636596000
1	7.797757000	6.148661000	2.386664000
1	-6.610197000	-6.832647000	0.314446000
1	-1.440487000	-5.921345000	1.396273000
1	-8.428730000	-1.342195000	-4.426849000
1	-8.485659000	-5.565679000	-1.149553000
1	0.295100000	-3.927561000	0.861855000
1	0.271893000	0.437467000	-2.228081000
1	-6.556840000	0.417558000	-5.267030000
1	-1.444203000	1.467339000	-4.039746000
6	-3.100268000	-1.435242000	2.798945000
6	-2.331200000	-1.374020000	3.978280000
6	-4.257676000	-2.236050000	2.796198000
6	-2.703340000	-2.091710000	5.119851000
1	-1.444339000	-0.745663000	4.003510000
6	-4.631173000	-2.950718000	3.939875000
1	-4.841164000	-2.300582000	1.883495000
6	-3.857445000	-2.883781000	5.104884000
1	-2.097535000	-2.027808000	6.019592000
1	-5.525788000	-3.566371000	3.917209000
1	-4.149636000	-3.441020000	5.990178000

1	-3.421588000	-1.016671000	0.674249000

$^4Pr_{DS,alpha,S1}$

26	-3.709558000	-3.321237000	-1.666337000
16	-2.780377000	-4.561184000	-3.361855000
1	-2.547313000	-5.719682000	-2.644802000
6	6.014899000	1.318900000	0.763406000
6	5.106956000	0.530124000	1.747615000
6	3.608550000	0.918662000	1.722345000
6	2.924393000	0.513754000	0.401539000
6	2.874511000	0.284221000	2.920608000
6	6.188348000	2.780431000	1.138717000
8	5.492117000	3.696436000	0.624149000
1	5.592799000	1.291030000	-0.243867000
1	5.199220000	-0.539281000	1.509599000
1	5.497653000	0.655391000	2.768603000
1	3.537349000	2.010827000	1.824750000
1	1.878341000	0.839713000	0.388418000
1	3.415840000	0.960378000	-0.470348000
1	2.949416000	-0.578134000	0.277056000
1	1.816377000	0.567761000	2.919634000
1	3.312600000	0.603587000	3.875175000
1	2.928480000	-0.811991000	2.876780000
7	7.144573000	3.042721000	2.080058000
6	7.502986000	4.388655000	2.534287000
6	7.316927000	4.607071000	4.056603000
6	5.877916000	4.722307000	4.521738000
6	5.248301000	5.980295000	4.573882000
6	3.927119000	6.106874000	5.021271000
6	3.209239000	4.969885000	5.420610000
6	3.822152000	3.711392000	5.365764000
6	5.145323000	3.592141000	4.923653000
1	7.693063000	2.268706000	2.428804000
1	6.885892000	5.086211000	1.963673000
1	7.819597000	3.791811000	4.594605000
1	7.856287000	5.526421000	4.318446000
1	5.804156000	6.868115000	4.284064000
1	3.468345000	7.089195000	5.080117000
1	2.195233000	5.068328000	5.796841000
1	3.276824000	2.827322000	5.679944000
1	5.617627000	2.614618000	4.903706000
6	3.355330000	3.864243000	-7.879432000
6	2.336028000	4.055964000	-6.736044000
6	1.319917000	2.910294000	-6.589438000
6	4.314048000	2.679102000	-7.673398000
1	2.809031000	3.732077000	-8.825383000
1	2.883332000	4.184941000	-5.788643000
1	1.791160000	4.996421000	-6.905496000
1	0.767062000	2.761644000	-7.526648000
1	1.812852000	1.962592000	-6.341683000

1	0.592138000	3.126106000	-5.799493000
1	5.039141000	2.609533000	-8.493204000
1	3.774147000	1.726727000	-7.626818000
1	4.876903000	2.789422000	-6.737069000
6	-0.296201000	5.833295000	-3.330984000
6	0.761609000	5.699050000	-2.205495000
6	1.616454000	4.421913000	-2.287300000
6	2.551841000	4.270989000	-1.073698000
7	1.760810000	4.008395000	0.137985000
6	2.028839000	4.480359000	1.359170000
7	3.231071000	5.049795000	1.639989000
7	1.085093000	4.396403000	2.317795000
6	-1.324158000	4.710485000	-3.328165000
8	-1.181296000	3.703323000	-4.083038000
1	0.198928000	5.795476000	-4.306103000
1	0.268234000	5.728725000	-1.225910000
1	1.420133000	6.578800000	-2.249623000
1	0.970110000	3.538769000	-2.358679000
1	2.226328000	4.438606000	-3.200458000
1	3.269982000	3.459440000	-1.241288000
1	3.134454000	5.188260000	-0.926435000
1	0.937469000	3.348605000	0.049794000
1	4.065443000	4.756477000	1.134239000
1	3.367351000	5.505798000	2.529682000
1	0.121196000	4.034327000	2.097958000
1	1.352179000	4.524291000	3.281557000
7	-2.379210000	4.802097000	-2.476111000
6	-3.369776000	3.698633000	-2.382399000
6	-4.432464000	4.238772000	-1.406472000
6	-3.659384000	5.253409000	-0.532789000
6	-2.676869000	5.901817000	-1.527130000
1	-2.870185000	2.801751000	-2.001328000
1	-5.234013000	4.746124000	-1.957988000
1	-4.889624000	3.438237000	-0.817807000
1	-4.312891000	5.993132000	-0.060146000
1	-3.099135000	4.737390000	0.255855000
1	-1.770439000	6.259506000	-1.035202000
1	-3.141293000	6.748245000	-2.054345000
6	-4.087370000	-0.790940000	-8.192058000
6	-4.299504000	0.610567000	-7.662090000
6	-5.544980000	1.251654000	-7.786800000
6	-5.749818000	2.533728000	-7.261737000
6	-4.709034000	3.197721000	-6.602033000
6	-3.459510000	2.575582000	-6.471804000
6	-3.261931000	1.294481000	-7.002213000
1	-3.075683000	-0.919157000	-8.592172000
1	-4.217053000	-1.537267000	-7.396611000
1	-6.355259000	0.744256000	-8.304202000
1	-6.718377000	3.012900000	-7.372793000
1	-4.866090000	4.193727000	-6.198535000

1	-2.647609000	3.074427000	-5.950871000
1	-2.290947000	0.817396000	-6.899972000
6	-3.476754000	-5.945680000	0.579333000
6	-2.409071000	-5.061612000	0.512776000
6	-1.186959000	-5.176490000	1.277099000
6	-0.385088000	-4.128767000	0.913610000
6	-1.107212000	-3.360289000	-0.077348000
7	-2.348834000	-3.938847000	-0.305156000
6	-0.623871000	-2.210175000	-0.689011000
6	-1.297949000	-1.454725000	-1.637182000
6	-0.773161000	-0.249068000	-2.246934000
6	-1.718145000	0.208579000	-3.121145000
6	-2.834513000	-0.713889000	-3.054590000
7	-2.568983000	-1.718322000	-2.130036000
6	-3.997166000	-0.606808000	-3.803042000
6	-5.060084000	-1.497394000	-3.737381000
6	-6.264905000	-1.402350000	-4.535519000
6	-7.054991000	-2.462667000	-4.192367000
6	-6.343292000	-3.217922000	-3.181003000
7	-5.128281000	-2.607827000	-2.908376000
6	-6.819779000	-4.375544000	-2.584482000
6	-6.153335000	-5.123496000	-1.624129000
6	-6.685000000	-6.321935000	-1.007009000
6	-5.750562000	-6.763631000	-0.114079000
6	-4.636492000	-5.840688000	-0.175230000
7	-4.900307000	-4.845154000	-1.104054000
1	-3.395474000	-6.782850000	1.264655000
1	0.366559000	-1.871958000	-0.402376000
1	-4.072699000	0.221021000	-4.498918000
1	-7.797208000	-4.728675000	-2.896920000
8	-1.325948000	3.290874000	1.730960000
6	-1.256721000	2.398758000	0.792894000
8	-0.247121000	2.255000000	-0.014818000
6	-2.406105000	1.503918000	0.583037000
6	-3.528227000	1.395460000	1.518737000
1	-4.716282000	-1.239689000	-0.429138000
1	-2.399761000	0.937008000	-0.346549000
1	-4.242601000	2.219187000	1.558063000
8	-4.573550000	-2.164453000	-0.159173000
1	-4.800490000	-1.031486000	-8.987728000
1	3.945116000	4.785992000	-7.990723000
1	-3.768698000	3.462322000	-3.372861000
1	-0.790607000	6.809035000	-3.246962000
1	6.999890000	0.835243000	0.719520000
1	8.552841000	4.580513000	2.276390000
1	-5.795955000	-7.629100000	0.531368000
1	-0.978603000	-5.964279000	1.987025000
1	-8.028699000	-2.725897000	-4.580205000
1	-7.648213000	-6.753922000	-1.237862000
1	0.607184000	-3.890526000	1.269357000

1	0.168796000	0.208807000	-1.989051000
1	-6.456025000	-0.623286000	-5.259663000
1	-1.679796000	1.102824000	-3.728000000
6	-3.757558000	0.289192000	2.377271000
6	-2.898241000	-0.857803000	2.379547000
6	-4.887186000	0.263427000	3.254247000
6	-3.166947000	-1.953109000	3.198180000
1	-2.025030000	-0.860771000	1.734744000
6	-5.141860000	-0.834650000	4.067453000
1	-5.550919000	1.123157000	3.274977000
6	-4.289802000	-1.953451000	4.046331000
1	-2.501251000	-2.810779000	3.176719000
1	-6.008392000	-0.829588000	4.721672000
1	-4.497599000	-2.811393000	4.676928000
1	-4.129184000	-2.208452000	0.712365000

$^{4}TS1_{HA,beta,S1}$

26	-4.547929000	-2.673411000	-2.076842000
16	-5.280901000	-4.468861000	-3.513994000
1	-5.997464000	-5.177677000	-2.568482000
6	6.547232000	4.256452000	0.529636000
6	6.421826000	2.857393000	1.192050000
6	4.984523000	2.286644000	1.267945000
6	4.414484000	1.961014000	-0.126651000
6	4.963007000	1.030401000	2.161791000
6	5.898664000	5.370881000	1.333167000
8	4.777760000	5.857985000	1.026440000
1	6.073868000	4.252752000	-0.454487000
1	7.050437000	2.156473000	0.624395000
1	6.847785000	2.902921000	2.205928000
1	4.339250000	3.044771000	1.736413000
1	3.384294000	1.595722000	-0.058589000
1	4.399551000	2.839611000	-0.781073000
1	5.018998000	1.185385000	-0.616545000
1	3.946913000	0.629266000	2.243625000
1	5.327252000	1.250187000	3.173955000
1	5.599766000	0.241059000	1.740485000
7	6.605134000	5.815255000	2.415922000
6	6.173235000	6.916991000	3.280206000
6	5.976054000	6.513345000	4.762874000
6	4.752009000	5.663789000	5.045556000
6	3.528508000	6.271663000	5.384586000
6	2.395532000	5.499583000	5.671637000
6	2.467883000	4.099894000	5.617595000
6	3.677414000	3.483343000	5.272538000
6	4.808143000	4.260260000	4.993950000
1	7.512449000	5.406602000	2.593154000
1	5.243093000	7.300497000	2.854833000
1	6.879639000	5.994366000	5.110944000
1	5.912989000	7.442842000	5.343310000

1	3.469631000	7.355126000	5.446623000
1	1.468463000	5.986580000	5.958581000
1	1.599793000	3.497734000	5.869216000
1	3.743980000	2.401010000	5.232905000
1	5.746556000	3.772061000	4.749071000
6	2.909362000	0.685116000	-3.989318000
6	1.832527000	1.791137000	-4.009710000
6	0.570816000	1.436378000	-4.814677000
6	2.488966000	-0.567282000	-3.199430000
1	3.170680000	0.405778000	-5.022248000
1	1.539657000	2.011251000	-2.974747000
1	2.272210000	2.709144000	-4.427287000
1	0.819418000	1.226897000	-5.865194000
1	0.071651000	0.550730000	-4.403480000
1	-0.146857000	2.264117000	-4.786499000
1	3.307817000	-1.296037000	-3.143174000
1	1.630555000	-1.068035000	-3.662474000
1	2.200177000	-0.288686000	-2.178658000
6	-1.614963000	6.126084000	-2.347649000
6	-0.457812000	5.976563000	-1.326727000
6	0.814351000	5.334184000	-1.907606000
6	1.908660000	5.130226000	-0.842585000
7	1.477551000	4.148174000	0.163989000
6	1.487961000	4.328174000	1.490427000
7	2.220893000	5.319857000	2.060517000
7	0.747056000	3.512653000	2.264382000
6	-2.091166000	4.795088000	-2.916211000
8	-1.640107000	4.379311000	-4.022678000
1	-1.272778000	6.717064000	-3.202885000
1	-0.794024000	5.379753000	-0.469452000
1	-0.213969000	6.975699000	-0.936190000
1	0.573612000	4.370004000	-2.370682000
1	1.221398000	5.968097000	-2.707238000
1	2.836413000	4.788072000	-1.315688000
1	2.132157000	6.078823000	-0.344725000
1	1.132056000	3.213897000	-0.180481000
1	3.062846000	5.670591000	1.605634000
1	2.111759000	5.512944000	3.044607000
1	0.066245000	2.835982000	1.829336000
1	0.933697000	3.466916000	3.254009000
7	-2.976145000	4.061231000	-2.190720000
6	-3.416005000	2.721358000	-2.662494000
6	-4.421993000	2.253284000	-1.591418000
6	-4.030557000	3.052336000	-0.326536000
6	-3.582153000	4.415696000	-0.884831000
1	-2.542281000	2.064477000	-2.738860000
1	-5.443610000	2.507382000	-1.900520000
1	-4.381805000	1.171821000	-1.433486000
1	-4.857050000	3.150075000	0.384340000
1	-3.190372000	2.573741000	0.190157000

1	-2.860833000	4.913628000	-0.233843000
1	-4.439010000	5.090593000	-1.031401000
6	-2.427414000	0.261782000	-9.328019000
6	-3.102439000	1.381814000	-8.564605000
6	-4.404074000	1.795879000	-8.904206000
6	-5.028193000	2.837791000	-8.210426000
6	-4.361241000	3.482920000	-7.159264000
6	-3.067622000	3.081425000	-6.803907000
6	-2.448563000	2.038111000	-7.507562000
1	-2.303803000	0.518278000	-10.388136000
1	-1.433963000	0.045654000	-8.921876000
1	-4.927262000	1.299947000	-9.718016000
1	-6.031179000	3.147007000	-8.490762000
1	-4.844341000	4.293786000	-6.622427000
1	-2.545943000	3.567879000	-5.984104000
1	-1.440432000	1.737850000	-7.234240000
6	-5.714452000	-4.360509000	0.681719000
6	-4.458523000	-4.720260000	0.212235000
6	-3.615695000	-5.743201000	0.795218000
6	-2.490206000	-5.820842000	0.025892000
6	-2.628585000	-4.841622000	-1.031134000
7	-3.841411000	-4.184037000	-0.902812000
6	-1.674140000	-4.588248000	-2.003993000
6	-1.777398000	-3.626267000	-2.997250000
6	-0.750036000	-3.340881000	-3.976141000
6	-1.204492000	-2.306963000	-4.746067000
6	-2.518657000	-1.955112000	-4.250813000
7	-2.851882000	-2.770567000	-3.186884000
6	-3.330065000	-0.964299000	-4.790178000
6	-4.616061000	-0.675277000	-4.366760000
6	-5.486932000	0.294206000	-5.000999000
6	-6.681422000	0.247770000	-4.341825000
6	-6.553095000	-0.739307000	-3.289831000
7	-5.283348000	-1.292629000	-3.316405000
6	-7.548473000	-1.057843000	-2.380746000
6	-7.409827000	-1.961144000	-1.338449000
6	-8.423343000	-2.229463000	-0.341708000
6	-7.895966000	-3.134521000	0.534692000
6	-6.557975000	-3.441942000	0.078182000
7	-6.276771000	-2.712971000	-1.071484000
1	-6.077552000	-4.861292000	1.572961000
1	-0.765567000	-5.179822000	-1.975959000
1	-2.940704000	-0.391243000	-5.624677000
1	-8.498036000	-0.541953000	-2.474719000
8	-0.891463000	1.738260000	1.049402000
6	-0.380075000	1.228907000	-0.017707000
8	0.664412000	1.688146000	-0.619218000
6	-1.092854000	0.030255000	-0.666579000
6	-1.802804000	-0.863968000	0.334762000
1	-1.838606000	0.447893000	-1.355925000

1	-0.371815000	-0.504740000	-1.287879000
1	-2.407988000	-0.270883000	1.026381000
8	-3.859259000	-1.468610000	-1.026384000
1	-3.014612000	-0.664431000	-9.287359000
1	3.826816000	1.094262000	-3.539934000
1	-3.853885000	2.794236000	-3.661972000
1	-2.445136000	6.667936000	-1.878104000
1	7.610790000	4.488640000	0.382287000
1	6.921613000	7.718717000	3.229807000
1	-8.358389000	-3.568509000	1.409441000
1	-3.866887000	-6.315922000	1.676123000
1	-7.576382000	0.820879000	-4.536273000
1	-9.401705000	-1.771521000	-0.325489000
1	-1.631785000	-6.462958000	0.155884000
1	0.189349000	-3.869035000	-4.050582000
1	-5.206388000	0.909908000	-5.843064000
1	-0.712465000	-1.820468000	-5.575171000
6	-1.071433000	-1.941430000	1.026819000
6	-1.532635000	-2.389253000	2.290025000
6	0.076623000	-2.569750000	0.487447000
6	-0.874657000	-3.402250000	2.985055000
1	-2.412809000	-1.919142000	2.718969000
6	0.737985000	-3.581048000	1.188089000
1	0.457270000	-2.257852000	-0.478397000
6	0.268721000	-4.003732000	2.438633000
1	-1.245773000	-3.721254000	3.954492000
1	1.624552000	-4.039056000	0.759048000
1	0.786474000	-4.788997000	2.981049000
1	-2.790965000	-1.375774000	-0.362479000

^{4}IM1$_{beta,S1}$

26	-4.794494000	-2.556596000	-2.116289000
16	-5.715030000	-4.203930000	-3.529438000
1	-6.913249000	-4.376199000	-2.863833000
6	6.470433000	4.359662000	0.657818000
6	6.219684000	2.967914000	1.302142000
6	4.757728000	2.462266000	1.242239000
6	4.305720000	2.156246000	-0.199265000
6	4.593424000	1.212924000	2.130829000
6	5.821685000	5.503994000	1.416823000
8	4.711043000	5.993244000	1.077479000
1	6.073372000	4.380818000	-0.359622000
1	6.866623000	2.238999000	0.793330000
1	6.550812000	2.997623000	2.351139000
1	4.106594000	3.251031000	1.646050000
1	3.257339000	1.840175000	-0.222770000
1	4.395929000	3.031367000	-0.852680000
1	4.915545000	1.349990000	-0.629882000
1	3.556783000	0.859424000	2.114311000
1	4.867025000	1.421842000	3.173499000

1	5.231152000	0.393009000	1.774222000
7	6.513286000	5.968421000	2.501211000
6	6.078714000	7.095737000	3.330136000
6	5.870284000	6.734563000	4.822183000
6	4.642745000	5.895293000	5.120013000
6	3.420091000	6.514585000	5.441208000
6	2.283263000	5.752775000	5.740457000
6	2.350993000	4.351992000	5.717306000
6	3.559953000	3.723987000	5.391089000
6	4.694400000	4.490746000	5.099882000
1	7.413706000	5.556273000	2.703422000
1	5.152916000	7.470113000	2.887517000
1	6.770510000	6.224036000	5.190942000
1	5.805060000	7.680052000	5.375860000
1	3.364701000	7.599335000	5.479356000
1	1.356551000	6.248998000	6.012225000
1	1.479716000	3.758373000	5.977894000
1	3.622715000	2.640764000	5.375830000
1	5.631772000	3.994131000	4.868374000
6	2.987819000	0.327798000	-3.984530000
6	1.947866000	1.457224000	-3.822145000
6	0.701549000	1.301804000	-4.710842000
6	2.517207000	-1.036092000	-3.449552000
1	3.257715000	0.231574000	-5.047968000
1	1.633592000	1.519010000	-2.771058000
1	2.432277000	2.415635000	-4.062515000
1	0.981144000	1.257550000	-5.773666000
1	0.147138000	0.385484000	-4.471492000
1	0.018980000	2.147253000	-4.570169000
1	3.290082000	-1.804318000	-3.579792000
1	1.612453000	-1.381659000	-3.962816000
1	2.286176000	-0.968214000	-2.378582000
6	-1.616213000	6.071156000	-2.504626000
6	-0.487037000	6.097645000	-1.443002000
6	0.832976000	5.455600000	-1.906689000
6	1.851367000	5.327262000	-0.759385000
7	1.369718000	4.367024000	0.246042000
6	1.394941000	4.550571000	1.570765000
7	2.136573000	5.544697000	2.127644000
7	0.662585000	3.742479000	2.361528000
6	-1.965155000	4.659261000	-2.958564000
8	-1.456929000	4.193026000	-4.020325000
1	-1.292773000	6.608852000	-3.401218000
1	-0.825086000	5.583195000	-0.535020000
1	-0.303448000	7.144242000	-1.158873000
1	0.642097000	4.464052000	-2.333750000
1	1.282261000	6.056703000	-2.708749000
1	2.824154000	5.003834000	-1.147842000
1	2.003914000	6.299359000	-0.278527000
1	1.005205000	3.438002000	-0.098116000

1	2.979286000	5.882257000	1.664831000
1	2.042826000	5.737784000	3.113400000
1	0.012795000	3.022647000	1.950242000
1	0.861068000	3.713146000	3.349638000
7	-2.788785000	3.914260000	-2.177550000
6	-3.093956000	2.499707000	-2.523195000
6	-4.203040000	2.107399000	-1.531679000
6	-3.931749000	3.007761000	-0.305417000
6	-3.469348000	4.339585000	-0.929283000
1	-2.186884000	1.896538000	-2.395857000
1	-5.186359000	2.339022000	-1.961460000
1	-4.184384000	1.040188000	-1.293513000
1	-4.812864000	3.137797000	0.330955000
1	-3.126175000	2.586685000	0.307867000
1	-2.791754000	4.890217000	-0.272601000
1	-4.324969000	4.990098000	-1.164761000
6	-2.201241000	0.112140000	-9.343570000
6	-2.873216000	1.244449000	-8.595387000
6	-4.151229000	1.694074000	-8.976875000
6	-4.770716000	2.749267000	-8.299304000
6	-4.122862000	3.372196000	-7.222701000
6	-2.853117000	2.935038000	-6.825572000
6	-2.238527000	1.878418000	-7.513251000
1	-1.232968000	-0.138735000	-8.898423000
1	-2.817935000	-0.795596000	-9.340157000
1	-4.659318000	1.216132000	-9.810802000
1	-5.754411000	3.087314000	-8.612953000
1	-4.601650000	4.194232000	-6.699130000
1	-2.344908000	3.405058000	-5.987841000
1	-1.248721000	1.550296000	-7.207046000
6	-5.925743000	-4.234862000	0.675204000
6	-4.674869000	-4.578233000	0.188331000
6	-3.832576000	-5.615255000	0.739326000
6	-2.720776000	-5.696383000	-0.050508000
6	-2.865632000	-4.709427000	-1.095435000
7	-4.056232000	-4.016994000	-0.920256000
6	-1.958031000	-4.508913000	-2.121412000
6	-2.105325000	-3.579120000	-3.138019000
6	-1.126755000	-3.335934000	-4.173326000
6	-1.599283000	-2.310404000	-4.943802000
6	-2.876275000	-1.915802000	-4.393972000
7	-3.175287000	-2.710376000	-3.298420000
6	-3.661911000	-0.888424000	-4.892446000
6	-4.890628000	-0.518721000	-4.374318000
6	-5.736265000	0.511894000	-4.937978000
6	-6.880555000	0.541412000	-4.192815000
6	-6.747585000	-0.463991000	-3.160629000
7	-5.518912000	-1.093342000	-3.279331000
6	-7.714580000	-0.748909000	-2.211965000
6	-7.588376000	-1.709518000	-1.221680000

6	-8.571740000	-1.960508000	-0.193302000
6	-8.064830000	-2.932326000	0.623111000
6	-6.766053000	-3.293566000	0.103284000
7	-6.497915000	-2.547317000	-1.037582000
1	-6.278853000	-4.750657000	1.561208000
1	-1.059195000	-5.114164000	-2.120188000
1	-3.293924000	-0.339459000	-5.751877000
1	-8.631143000	-0.170087000	-2.237334000
8	-0.825434000	1.767320000	1.273604000
6	-0.275904000	1.278682000	0.216521000
8	0.581871000	1.899123000	-0.519668000
6	-0.682876000	-0.138333000	-0.228466000
6	-0.922085000	-1.060681000	0.924329000
1	-1.610474000	-0.026692000	-0.816790000
1	0.080189000	-0.500154000	-0.920126000
1	-1.562259000	-0.663340000	1.708406000
8	-4.003319000	-1.309698000	-1.049574000
1	-2.023942000	0.376220000	-10.394258000
1	3.910514000	0.613255000	-3.457490000
1	-3.397151000	2.426169000	-3.570275000
1	-2.499987000	6.584666000	-2.105920000
1	7.552936000	4.533664000	0.592215000
1	6.829626000	7.893961000	3.262631000
1	-8.512922000	-3.369402000	1.503597000
1	-4.076860000	-6.199290000	1.614582000
1	-7.746191000	1.175465000	-4.318452000
1	-9.516360000	-1.442584000	-0.112917000
1	-1.867004000	-6.348195000	0.057071000
1	-0.199818000	-3.880576000	-4.277050000
1	-5.472108000	1.115496000	-5.794115000
1	-1.137710000	-1.848029000	-5.803561000
6	-0.320645000	-2.328464000	1.138397000
6	-0.614016000	-3.056771000	2.336784000
6	0.592859000	-2.938994000	0.221249000
6	-0.036379000	-4.293108000	2.596645000
1	-1.303751000	-2.617298000	3.052178000
6	1.167991000	-4.176433000	0.494680000
1	0.843190000	-2.428791000	-0.701935000
6	0.862620000	-4.867347000	1.679905000
1	-0.278796000	-4.817601000	3.516536000
1	1.865833000	-4.609686000	-0.216684000
1	1.319377000	-5.829840000	1.888029000
1	-3.405729000	-1.685154000	-0.365745000

$^{4}TS2_{reb,beta,S1}$

26	-4.375033000	-2.965708000	-2.345078000
16	-5.617260000	-4.441174000	-3.765963000
1	-6.366794000	-3.498360000	-4.443569000
6	6.577100000	4.163963000	0.558883000
6	6.290403000	2.795201000	1.236729000

6	4.811078000	2.339553000	1.207356000
6	4.334225000	2.002518000	-0.219196000
6	4.612323000	1.126706000	2.138412000
6	5.988463000	5.345216000	1.310252000
8	4.886216000	5.865816000	0.990234000
1	6.157050000	4.179843000	-0.449410000
1	6.904651000	2.034004000	0.734849000
1	6.638166000	2.836156000	2.279995000
1	4.192226000	3.163710000	1.590439000
1	3.274699000	1.725044000	-0.223788000
1	4.450289000	2.851356000	-0.902572000
1	4.910430000	1.160931000	-0.627918000
1	3.563803000	0.809318000	2.141259000
1	4.901460000	1.360417000	3.171536000
1	5.219270000	0.274376000	1.804710000
7	6.724260000	5.806929000	2.365886000
6	6.352295000	6.966466000	3.180813000
6	6.145656000	6.640262000	4.681328000
6	4.877120000	5.876798000	5.010477000
6	3.698900000	6.570424000	5.345701000
6	2.526045000	5.880534000	5.678085000
6	2.512108000	4.477987000	5.675587000
6	3.675756000	3.776248000	5.334773000
6	4.846355000	4.471451000	5.008762000
1	7.615354000	5.368216000	2.553018000
1	5.439203000	7.376125000	2.743154000
1	7.021043000	6.085418000	5.045640000
1	6.141646000	7.596084000	5.220749000
1	3.707134000	7.656932000	5.369614000
1	1.635618000	6.433828000	5.960760000
1	1.613769000	3.939974000	5.963693000
1	3.675802000	2.691058000	5.336183000
1	5.748033000	3.917878000	4.764861000
6	2.874584000	0.282830000	-3.973948000
6	1.890785000	1.465073000	-3.839907000
6	0.661416000	1.372512000	-4.760812000
6	2.314188000	-1.053798000	-3.457443000
1	3.173280000	0.174193000	-5.028495000
1	1.555798000	1.539769000	-2.795978000
1	2.429597000	2.397802000	-4.065808000
1	0.965567000	1.321093000	-5.816426000
1	0.061010000	0.480659000	-4.542718000
1	0.014962000	2.248006000	-4.633036000
1	3.057397000	-1.856995000	-3.541539000
1	1.423933000	-1.363985000	-4.016020000
1	2.027974000	-0.966964000	-2.401446000
6	-1.471424000	6.197112000	-2.474619000
6	-0.332203000	6.139811000	-1.424518000
6	0.953053000	5.454722000	-1.923542000
6	2.001827000	5.294051000	-0.807469000

7	1.516052000	4.361645000	0.221981000
6	1.537783000	4.576810000	1.542524000
7	2.313242000	5.553206000	2.081580000
7	0.766579000	3.815534000	2.342805000
6	-1.894618000	4.817907000	-2.962772000
8	-1.412953000	4.350036000	-4.035089000
1	-1.128531000	6.741711000	-3.359667000
1	-0.684016000	5.614852000	-0.527485000
1	-0.098738000	7.168555000	-1.112991000
1	0.718587000	4.469436000	-2.343682000
1	1.397852000	6.040499000	-2.739360000
1	2.946962000	4.927227000	-1.223817000
1	2.210320000	6.262027000	-0.340555000
1	1.133369000	3.434777000	-0.099265000
1	3.171081000	5.845890000	1.615094000
1	2.218405000	5.777375000	3.060649000
1	0.070800000	3.141084000	1.934710000
1	0.954835000	3.793129000	3.332960000
7	-2.755519000	4.094826000	-2.198983000
6	-3.117451000	2.703884000	-2.579190000
6	-4.174980000	2.292761000	-1.537252000
6	-3.871416000	3.187764000	-0.313398000
6	-3.417601000	4.519517000	-0.942273000
1	-2.219803000	2.076250000	-2.539158000
1	-5.180656000	2.500342000	-1.921592000
1	-4.125198000	1.224283000	-1.309602000
1	-4.739584000	3.319068000	0.340338000
1	-3.054248000	2.766447000	0.283181000
1	-2.729763000	5.067530000	-0.294690000
1	-4.275662000	5.171667000	-1.164300000
6	-2.394461000	0.220184000	-9.259216000
6	-3.061060000	1.343780000	-8.493689000
6	-4.391507000	1.708984000	-8.765854000
6	-5.016746000	2.731472000	-8.043191000
6	-4.319067000	3.407171000	-7.032854000
6	-2.992088000	3.057327000	-6.748098000
6	-2.373625000	2.033980000	-7.478862000
1	-1.308250000	0.355556000	-9.301307000
1	-2.585681000	-0.754240000	-8.788104000
1	-4.937237000	1.190891000	-9.550185000
1	-6.042559000	3.004443000	-8.274081000
1	-4.800543000	4.206925000	-6.477885000
1	-2.441447000	3.570277000	-5.964283000
1	-1.340840000	1.775372000	-7.259985000
6	-5.068180000	-5.025954000	0.328172000
6	-3.957736000	-5.323296000	-0.449695000
6	-3.093268000	-6.464388000	-0.244640000
6	-2.142226000	-6.437571000	-1.225309000
6	-2.399383000	-5.274036000	-2.044528000
7	-3.508514000	-4.601111000	-1.547564000

6	-1.625497000	-4.886130000	-3.129628000
6	-1.843942000	-3.757729000	-3.905668000
6	-1.002061000	-3.317558000	-5.000320000
6	-1.542748000	-2.159602000	-5.485155000
6	-2.730088000	-1.880142000	-4.701448000
7	-2.887847000	-2.866750000	-3.747011000
6	-3.597962000	-0.820839000	-4.917779000
6	-4.772760000	-0.596030000	-4.212582000
6	-5.709976000	0.468858000	-4.506090000
6	-6.742957000	0.352331000	-3.619020000
6	-6.451565000	-0.777845000	-2.761606000
7	-5.249707000	-1.347510000	-3.150330000
6	-7.244758000	-1.184035000	-1.696516000
6	-6.958768000	-2.237178000	-0.841636000
6	-7.762209000	-2.633114000	0.298322000
6	-7.139975000	-3.701941000	0.878881000
6	-5.951761000	-3.982248000	0.096839000
7	-5.864525000	-3.074360000	-0.940141000
1	-5.275311000	-5.681105000	1.167046000
1	-0.773966000	-5.509113000	-3.380190000
1	-3.356022000	-0.124917000	-5.713443000
1	-8.152899000	-0.620798000	-1.511141000
8	-0.879455000	1.972933000	1.221648000
6	-0.344305000	1.413122000	0.193066000
8	0.641672000	1.894292000	-0.479396000
6	-0.932097000	0.072256000	-0.301146000
6	-1.916776000	-0.526820000	0.642009000
1	-1.426003000	0.264831000	-1.261547000
1	-0.090764000	-0.590631000	-0.525581000
1	-2.891967000	-0.058284000	0.686395000
8	-3.365375000	-1.837407000	-1.195233000
1	-2.767395000	0.158845000	-10.287574000
1	3.793427000	0.520336000	-3.416838000
1	-3.489107000	2.675432000	-3.606697000
1	-2.324519000	6.744092000	-2.054539000
1	7.663267000	4.296640000	0.464972000
1	7.138986000	7.726872000	3.090623000
1	-7.448800000	-4.266117000	1.747046000
1	-3.217140000	-7.187453000	0.548325000
1	-7.627050000	0.967793000	-3.535301000
1	-8.678079000	-2.145941000	0.600257000
1	-1.332100000	-7.132510000	-1.392042000
1	-0.116390000	-3.835884000	-5.337846000
1	-5.576916000	1.192711000	-5.297175000
1	-1.188988000	-1.542903000	-6.298150000
6	-1.655437000	-1.566666000	1.576751000
6	-2.702541000	-2.011422000	2.442245000
6	-0.384603000	-2.205596000	1.703133000
6	-2.489842000	-3.023487000	3.370217000
1	-3.679432000	-1.546203000	2.354212000

6	-0.182847000	-3.217178000	2.637006000
1	0.437155000	-1.893122000	1.068705000
6	-1.228706000	-3.634954000	3.476440000
1	-3.302728000	-3.343568000	4.014782000
1	0.792832000	-3.687177000	2.716456000
1	-1.064078000	-4.426072000	4.201020000
1	-2.754330000	-2.420654000	-0.695724000

$^{4}Pr_{OH,beta,S1}$

26	-4.613998000	-2.900315000	-2.544646000
16	-5.472064000	-4.583994000	-4.126814000
1	-6.601641000	-4.935160000	-3.410907000
6	6.548261000	3.776036000	0.827914000
6	6.220747000	2.468729000	1.600455000
6	4.733590000	2.038982000	1.566445000
6	4.290827000	1.597708000	0.157533000
6	4.485907000	0.908078000	2.584979000
6	5.962211000	5.022460000	1.469021000
8	4.916328000	5.576138000	1.035537000
1	6.155261000	3.722453000	-0.189697000
1	6.831730000	1.660097000	1.174522000
1	6.543319000	2.582168000	2.646581000
1	4.121112000	2.902243000	1.865545000
1	3.224693000	1.348795000	0.138097000
1	4.451128000	2.384268000	-0.588436000
1	4.855626000	0.710823000	-0.161044000
1	3.432128000	0.607676000	2.582886000
1	4.749801000	1.219039000	3.604386000
1	5.087153000	0.022701000	2.338145000
7	6.638321000	5.507698000	2.553334000
6	6.267762000	6.728286000	3.273925000
6	5.935747000	6.497869000	4.769688000
6	4.602544000	5.827594000	5.041977000
6	3.439941000	6.604664000	5.204000000
6	2.205681000	6.005115000	5.483989000
6	2.113543000	4.610352000	5.601840000
6	3.261512000	3.825500000	5.433440000
6	4.493595000	4.431282000	5.158857000
1	7.486869000	5.036095000	2.834758000
1	5.410934000	7.154470000	2.747340000
1	6.747381000	5.915055000	5.226073000
1	5.951086000	7.480754000	5.258034000
1	3.507020000	7.687144000	5.133796000
1	1.325936000	6.623712000	5.633287000
1	1.164407000	4.144683000	5.850367000
1	3.200809000	2.746273000	5.529490000
1	5.381103000	3.815108000	5.050920000
6	3.016775000	0.506347000	-3.754175000
6	2.040822000	1.697873000	-3.862989000
6	0.920806000	1.509433000	-4.900614000

6	2.379111000	-0.747112000	-3.129773000
1	3.419000000	0.266911000	-4.751136000
1	1.591690000	1.865365000	-2.874760000
1	2.610588000	2.604459000	-4.117557000
1	1.337945000	1.382668000	-5.909780000
1	0.312729000	0.625335000	-4.675289000
1	0.250033000	2.375880000	-4.908204000
1	3.114538000	-1.552976000	-3.010565000
1	1.560189000	-1.132409000	-3.748601000
1	1.964733000	-0.506440000	-2.143353000
6	-1.319923000	6.080902000	-2.610671000
6	-0.192731000	5.915993000	-1.558426000
6	1.034073000	5.137723000	-2.066161000
6	2.090583000	4.913435000	-0.966579000
7	1.555952000	4.057596000	0.103790000
6	1.490651000	4.377349000	1.402733000
7	2.260537000	5.366405000	1.925510000
7	0.634043000	3.704568000	2.193719000
6	-1.874257000	4.752707000	-3.110478000
8	-1.441696000	4.247850000	-4.186583000
1	-0.926057000	6.596688000	-3.491828000
1	-0.589529000	5.407846000	-0.670586000
1	0.120882000	6.918507000	-1.231928000
1	0.723734000	4.167525000	-2.470873000
1	1.506443000	5.681346000	-2.896018000
1	2.984324000	4.445507000	-1.394786000
1	2.401460000	5.870911000	-0.538349000
1	1.171400000	3.115549000	-0.167131000
1	3.161765000	5.598177000	1.508768000
1	2.093636000	5.675190000	2.871332000
1	-0.079872000	3.059791000	1.765872000
1	0.749621000	3.739877000	3.194515000
7	-2.809129000	4.115224000	-2.356316000
6	-3.317425000	2.775709000	-2.753186000
6	-4.407991000	2.462937000	-1.709281000
6	-4.006770000	3.306589000	-0.477385000
6	-3.422562000	4.591810000	-1.092895000
1	-2.491721000	2.055176000	-2.728763000
1	-5.390151000	2.775457000	-2.085146000
1	-4.464456000	1.394573000	-1.482815000
1	-4.852076000	3.515999000	0.185564000
1	-3.230354000	2.796458000	0.103965000
1	-2.680071000	5.057457000	-0.441663000
1	-4.209075000	5.331314000	-1.306109000
6	-2.406246000	0.238641000	-9.569132000
6	-3.077529000	1.281819000	-8.700868000
6	-4.437998000	1.593628000	-8.875471000
6	-5.065628000	2.544510000	-8.062672000
6	-4.341221000	3.199012000	-7.056773000
6	-2.985320000	2.900038000	-6.866561000

6	-2.364501000	1.949940000	-7.688944000
1	-1.323522000	0.396511000	-9.617333000
1	-2.573587000	-0.775177000	-9.179638000
1	-5.004601000	1.092092000	-9.655669000
1	-6.113893000	2.781282000	-8.222377000
1	-4.824521000	3.945159000	-6.432959000
1	-2.417277000	3.396636000	-6.084516000
1	-1.308651000	1.733356000	-7.546850000
6	-4.960358000	-4.890316000	0.249171000
6	-3.741924000	-4.925454000	-0.405582000
6	-2.659784000	-5.822299000	-0.080349000
6	-1.654539000	-5.585946000	-0.975790000
6	-2.106822000	-4.539746000	-1.859691000
7	-3.385282000	-4.121406000	-1.488287000
6	-1.381977000	-4.041934000	-2.927875000
6	-1.826546000	-3.050362000	-3.787779000
6	-1.051389000	-2.509177000	-4.877593000
6	-1.808054000	-1.533430000	-5.465978000
6	-3.056140000	-1.471056000	-4.746209000
7	-3.061657000	-2.411301000	-3.718994000
6	-4.079392000	-0.584686000	-5.037664000
6	-5.272224000	-0.510982000	-4.339669000
6	-6.340020000	0.412099000	-4.649151000
6	-7.351460000	0.165406000	-3.764255000
6	-6.912629000	-0.909939000	-2.904071000
7	-5.629740000	-1.310525000	-3.259536000
6	-7.672078000	-1.461829000	-1.886880000
6	-7.251697000	-2.490739000	-1.060472000
6	-8.025479000	-3.027968000	0.032093000
6	-7.261781000	-3.989751000	0.633132000
6	-6.012023000	-4.052202000	-0.084871000
7	-6.020832000	-3.137125000	-1.133604000
1	-5.102062000	-5.565006000	1.086246000
1	-0.394702000	-4.454135000	-3.103657000
1	-3.932152000	0.101064000	-5.864177000
1	-8.661746000	-1.052355000	-1.716829000
8	-1.073714000	2.008437000	0.974831000
6	-0.505967000	1.282778000	0.070098000
8	0.637307000	1.555517000	-0.464289000
6	-1.255016000	0.041448000	-0.413930000
6	-2.032342000	-0.673294000	0.700986000
1	-1.997863000	0.371793000	-1.150845000
1	-0.560383000	-0.627991000	-0.929970000
1	-2.507916000	0.102340000	1.308064000
8	-3.173474000	-1.418125000	0.149892000
1	-2.796287000	0.257994000	-10.592900000
1	3.877370000	0.807952000	-3.138463000
1	-3.695138000	2.801089000	-3.778888000
1	-2.119618000	6.702288000	-2.188799000
1	7.638740000	3.885851000	0.754426000

1	7.096833000	7.444459000	3.202883000
1	-7.505748000	-4.600172000	1.490369000
1	-2.680348000	-6.537918000	0.728385000
1	-8.312324000	0.653722000	-3.690370000
1	-9.017771000	-2.695811000	0.300414000
1	-0.690989000	-6.069220000	-1.043495000
1	-0.056161000	-2.835403000	-5.141345000
1	-6.298824000	1.139413000	-5.446542000
1	-1.556544000	-0.897460000	-6.301367000
6	-1.190636000	-1.569042000	1.604943000
6	-1.838172000	-2.517165000	2.419142000
6	0.208800000	-1.464165000	1.679164000
6	-1.107960000	-3.336301000	3.284567000
1	-2.917067000	-2.607110000	2.354825000
6	0.940105000	-2.285069000	2.547510000
1	0.735197000	-0.744981000	1.061079000
6	0.286509000	-3.222844000	3.353271000
1	-1.625395000	-4.062425000	3.905081000
1	2.021796000	-2.192882000	2.587406000
1	0.855257000	-3.859699000	4.024404000
1	-2.873378000	-2.229580000	-0.313509000

$^{4}TS2_{DS,beta,S1}$

26	-4.617016000	-2.321233000	-1.846408000
16	-5.858188000	-4.060652000	-2.951693000
1	-5.013413000	-4.239537000	-4.030211000
6	6.229098000	3.868406000	0.864330000
6	5.810498000	2.506627000	1.485387000
6	4.317281000	2.131410000	1.324472000
6	3.938386000	1.867721000	-0.146016000
6	3.984952000	0.900876000	2.192173000
6	5.623557000	5.067680000	1.572118000
8	4.555801000	5.611596000	1.181070000
1	5.915448000	3.917195000	-0.181085000
1	6.424073000	1.721836000	1.020021000
1	6.070544000	2.510359000	2.554772000
1	3.713090000	2.974095000	1.689544000
1	2.870604000	1.643168000	-0.242280000
1	4.144971000	2.732726000	-0.786271000
1	4.503500000	1.012689000	-0.541857000
1	2.925608000	0.636326000	2.100941000
1	4.200518000	1.086891000	3.252518000
1	4.573684000	0.029021000	1.877042000
7	6.301623000	5.517310000	2.670721000
6	5.904647000	6.687048000	3.458600000
6	5.603287000	6.369221000	4.944657000
6	4.309043000	5.619560000	5.195442000
6	3.115532000	6.325998000	5.436303000
6	1.916126000	5.648721000	5.690854000
6	1.890808000	4.246254000	5.703205000

6	3.070669000	3.531696000	5.457583000
6	4.267527000	4.214473000	5.209805000
1	7.169199000	5.059714000	2.914592000
1	5.027125000	7.112709000	2.966669000
1	6.448573000	5.806721000	5.364183000
1	5.575833000	7.327177000	5.479690000
1	3.130507000	7.412625000	5.446365000
1	1.011754000	6.211764000	5.900453000
1	0.969019000	3.718650000	5.930051000
1	3.061815000	2.446555000	5.472007000
1	5.180432000	3.651758000	5.039229000
6	3.007382000	0.455753000	-4.064836000
6	1.929203000	1.548776000	-3.903079000
6	0.750040000	1.425002000	-4.883601000
6	2.540160000	-0.949823000	-3.648473000
1	3.346157000	0.433765000	-5.112342000
1	1.540330000	1.534304000	-2.875339000
1	2.403500000	2.531327000	-4.046442000
1	1.103446000	1.436996000	-5.924894000
1	0.192421000	0.493177000	-4.727343000
1	0.050276000	2.257257000	-4.749977000
1	3.337238000	-1.692042000	-3.784266000
1	1.673611000	-1.277366000	-4.234040000
1	2.247559000	-0.958962000	-2.590235000
6	-1.496771000	6.245484000	-2.769402000
6	-0.433513000	6.212438000	-1.640859000
6	0.848632000	5.441232000	-2.004076000
6	1.754975000	5.209826000	-0.783072000
7	1.117856000	4.279798000	0.165442000
6	1.116197000	4.419042000	1.496408000
7	1.911148000	5.339668000	2.103641000
7	0.307814000	3.645194000	2.246835000
6	-1.880501000	4.845898000	-3.233238000
8	-1.339730000	4.356097000	-4.268315000
1	-1.092617000	6.759463000	-3.646606000
1	-0.869505000	5.759544000	-0.741668000
1	-0.174952000	7.247589000	-1.374266000
1	0.596587000	4.475173000	-2.457007000
1	1.418755000	5.995209000	-2.761809000
1	2.726692000	4.812099000	-1.097839000
1	1.946025000	6.158936000	-0.271253000
1	0.676147000	3.409537000	-0.223852000
1	2.792710000	5.624798000	1.679276000
1	1.804558000	5.507432000	3.092994000
1	-0.341102000	2.937913000	1.816164000
1	0.465969000	3.600328000	3.241750000
7	-2.747915000	4.128689000	-2.475410000
6	-3.024873000	2.698383000	-2.783326000
6	-4.158782000	2.322229000	-1.815176000
6	-3.956942000	3.279123000	-0.618947000

6	-3.493036000	4.592209000	-1.279617000
1	-2.114435000	2.115730000	-2.600680000
1	-5.133360000	2.500833000	-2.286521000
1	-4.103379000	1.267497000	-1.534395000
1	-4.866549000	3.419766000	-0.025952000
1	-3.172815000	2.895208000	0.044565000
1	-2.860303000	5.188964000	-0.618578000
1	-4.350965000	5.210767000	-1.582894000
6	-2.208466000	0.010586000	-9.416936000
6	-2.900748000	1.121038000	-8.654844000
6	-4.223970000	1.485580000	-8.963582000
6	-4.854265000	2.535898000	-8.287027000
6	-4.171208000	3.237216000	-7.283843000
6	-2.855766000	2.882909000	-6.957580000
6	-2.231772000	1.830920000	-7.642113000
1	-1.346975000	-0.377395000	-8.863082000
1	-2.888022000	-0.825952000	-9.616295000
1	-4.759987000	0.944559000	-9.739304000
1	-5.874276000	2.807286000	-8.544405000
1	-4.658668000	4.053851000	-6.759943000
1	-2.320423000	3.412961000	-6.174337000
1	-1.209979000	1.563120000	-7.386677000
6	-5.384937000	-3.813896000	1.170000000
6	-4.215435000	-4.240737000	0.555458000
6	-3.318954000	-5.267909000	1.052738000
6	-2.311572000	-5.401725000	0.138357000
6	-2.573070000	-4.452762000	-0.928621000
7	-3.729124000	-3.758897000	-0.642705000
6	-1.777667000	-4.251878000	-2.049404000
6	-2.019782000	-3.332177000	-3.065204000
6	-1.133432000	-3.095088000	-4.187166000
6	-1.693049000	-2.105988000	-4.946876000
6	-2.938072000	-1.725408000	-4.311470000
7	-3.116227000	-2.492260000	-3.170519000
6	-3.817717000	-0.771807000	-4.812756000
6	-5.035790000	-0.420627000	-4.245161000
6	-5.993597000	0.511604000	-4.812097000
6	-7.062837000	0.555067000	-3.960470000
6	-6.770711000	-0.341899000	-2.857245000
7	-5.534529000	-0.919133000	-3.059269000
6	-7.580627000	-0.562875000	-1.751134000
6	-7.285014000	-1.402802000	-0.682679000
6	-8.137712000	-1.593076000	0.471142000
6	-7.516412000	-2.488711000	1.295872000
6	-6.273575000	-2.871765000	0.660712000
7	-6.146923000	-2.185192000	-0.539133000
1	-5.646196000	-4.272545000	2.117700000
1	-0.879926000	-4.853908000	-2.134781000
1	-3.535508000	-0.273577000	-5.733984000
1	-8.525330000	-0.031343000	-1.710849000

8	-1.119609000	1.615539000	1.181569000
6	-0.564700000	1.174757000	0.106035000
8	0.160875000	1.873759000	-0.694656000
6	-0.757550000	-0.314631000	-0.245219000
6	-0.535563000	-1.174652000	0.927178000
1	-1.829785000	-0.446382000	-0.575963000
1	-0.129825000	-0.558748000	-1.104200000
1	-1.006418000	-0.822716000	1.843779000
8	-3.617618000	-0.952802000	-0.965363000
1	-1.837696000	0.365236000	-10.388536000
1	3.887491000	0.727660000	-3.462643000
1	-3.295604000	2.582809000	-3.835229000
1	-2.374931000	6.805998000	-2.425520000
1	7.324343000	3.947037000	0.881909000
1	6.709681000	7.432141000	3.413587000
1	-7.860725000	-2.873977000	2.244644000
1	-3.457708000	-5.809601000	1.977313000
1	-7.969051000	1.135715000	-4.055799000
1	-9.087652000	-1.099218000	0.615328000
1	-1.457744000	-6.062342000	0.176310000
1	-0.206827000	-3.624535000	-4.355900000
1	-5.846912000	1.049723000	-5.737477000
1	-1.316362000	-1.665447000	-5.858196000
6	0.215992000	-2.373602000	0.998963000
6	0.299106000	-3.075004000	2.243491000
6	0.917357000	-2.928186000	-0.116657000
6	1.036639000	-4.245467000	2.364332000
1	-0.233126000	-2.673339000	3.101116000
6	1.656712000	-4.098885000	0.017668000
1	0.871828000	-2.427749000	-1.076619000
6	1.723042000	-4.766736000	1.252790000
1	1.083190000	-4.760140000	3.319133000
1	2.189576000	-4.498449000	-0.840003000
1	2.303641000	-5.678812000	1.349044000
1	-4.092107000	-0.654098000	-0.159149000

$^{4}Pr_{DS,beta,S1}$

26	-5.080833000	-3.330067000	-2.858692000
16	-6.178076000	-4.502899000	-4.763981000
1	-6.612794000	-3.387469000	-5.455081000
6	6.768087000	4.967002000	0.401239000
6	6.601980000	3.641543000	1.195577000
6	5.153104000	3.105596000	1.293374000
6	4.618362000	2.618902000	-0.067926000
6	5.076008000	1.969937000	2.333399000
6	6.168201000	6.172029000	1.104380000
8	5.004642000	6.587815000	0.857009000
1	6.281024000	4.884458000	-0.573275000
1	7.229352000	2.877827000	0.714025000
1	7.008081000	3.782541000	2.208752000

1	4.508985000	3.925275000	1.641607000
1	3.582289000	2.274996000	0.020632000
1	4.634227000	3.411711000	-0.824688000
1	5.226302000	1.783730000	-0.442590000
1	4.049527000	1.597968000	2.421909000
1	5.403674000	2.309449000	3.324726000
1	5.714002000	1.125688000	2.039230000
7	6.967103000	6.776947000	2.035034000
6	6.592718000	7.982165000	2.779188000
6	6.546860000	7.783888000	4.315062000
6	5.373264000	6.972605000	4.829233000
6	4.180327000	7.614702000	5.211251000
6	3.097243000	6.882913000	5.714239000
6	3.189432000	5.488775000	5.838395000
6	4.367844000	4.837072000	5.452255000
6	5.449137000	5.574795000	4.955906000
1	7.901863000	6.415840000	2.166343000
1	5.617661000	8.295022000	2.398956000
1	7.489924000	7.323130000	4.639409000
1	6.521180000	8.783199000	4.768359000
1	4.108725000	8.696552000	5.136419000
1	2.195893000	7.399604000	6.029672000
1	2.363303000	4.922038000	6.257782000
1	4.450266000	3.759505000	5.550848000
1	6.365505000	5.062371000	4.679378000
6	2.969013000	-0.282998000	-3.250410000
6	2.080926000	0.979655000	-3.224855000
6	0.830790000	0.888775000	-4.117312000
6	2.310211000	-1.528288000	-2.630923000
1	3.256300000	-0.503019000	-4.290485000
1	1.778101000	1.197603000	-2.190778000
1	2.685783000	1.838282000	-3.552671000
1	1.112680000	0.726608000	-5.168004000
1	0.179761000	0.057812000	-3.817806000
1	0.239211000	1.808550000	-4.055973000
1	2.989432000	-2.389789000	-2.651317000
1	1.396982000	-1.812240000	-3.167144000
1	2.041186000	-1.340391000	-1.582846000
6	-1.638529000	5.739364000	-2.188821000
6	-0.489282000	5.988505000	-1.178558000
6	0.890943000	5.511395000	-1.666290000
6	1.977938000	5.666884000	-0.587865000
7	1.685648000	4.787593000	0.554850000
6	1.826195000	5.122093000	1.841911000
7	2.554700000	6.210769000	2.204849000
7	1.224066000	4.372484000	2.785197000
6	-1.797738000	4.266730000	-2.541400000
8	-1.230829000	3.797663000	-3.571252000
1	-1.420893000	6.257324000	-3.128032000
1	-0.719458000	5.488625000	-0.229448000

1	-0.447413000	7.066256000	-0.962630000
1	0.837434000	4.461805000	-1.978735000
1	1.194053000	6.083823000	-2.553281000
1	2.965087000	5.432062000	-1.003440000
1	2.016205000	6.703457000	-0.233714000
1	1.359032000	3.805137000	0.348919000
1	3.333243000	6.522552000	1.625966000
1	2.544754000	6.514376000	3.166942000
1	0.566663000	3.595859000	2.514769000
1	1.518195000	4.460479000	3.745444000
7	-2.517595000	3.475693000	-1.704457000
6	-2.642649000	2.015443000	-1.953371000
6	-3.743330000	1.573344000	-0.974375000
6	-3.600111000	2.563306000	0.203935000
6	-3.247080000	3.898458000	-0.483251000
1	-1.681563000	1.530761000	-1.743277000
1	-4.728140000	1.681390000	-1.446110000
1	-3.635409000	0.526730000	-0.678512000
1	-4.509618000	2.643612000	0.807761000
1	-2.777315000	2.265524000	0.863593000
1	-2.625467000	4.533221000	0.153092000
1	-4.152052000	4.460967000	-0.755737000
6	-1.910604000	0.038550000	-9.049249000
6	-2.569130000	1.181569000	-8.306303000
6	-3.832907000	1.659770000	-8.693386000
6	-4.453292000	2.698961000	-7.990061000
6	-3.817780000	3.277213000	-6.883643000
6	-2.556587000	2.814031000	-6.482680000
6	-1.941906000	1.776570000	-7.195919000
1	-0.820741000	0.150650000	-9.070785000
1	-2.130462000	-0.926896000	-8.572519000
1	-4.331360000	1.215979000	-9.551119000
1	-5.427201000	3.058691000	-8.309613000
1	-4.294072000	4.089865000	-6.342894000
1	-2.055230000	3.251093000	-5.623602000
1	-0.957614000	1.431939000	-6.888360000
6	-6.316831000	-5.687630000	-0.666023000
6	-5.096725000	-5.949539000	-1.274826000
6	-4.335047000	-7.163944000	-1.097013000
6	-3.197454000	-7.041925000	-1.848988000
6	-3.248394000	-5.750192000	-2.493390000
7	-4.418392000	-5.093077000	-2.133231000
6	-2.255705000	-5.240352000	-3.319513000
6	-2.284208000	-3.990521000	-3.919079000
6	-1.244087000	-3.463039000	-4.776342000
6	-1.651256000	-2.224998000	-5.188189000
6	-2.947427000	-1.982369000	-4.591333000
7	-3.312631000	-3.065389000	-3.804648000
6	-3.714886000	-0.849374000	-4.813352000
6	-4.976235000	-0.630637000	-4.277466000

6	-5.759865000	0.564665000	-4.497828000
6	-6.915362000	0.424032000	-3.779147000
6	-6.851145000	-0.856589000	-3.110414000
7	-5.664900000	-1.497087000	-3.439925000
6	-7.822259000	-1.343096000	-2.246146000
6	-7.748304000	-2.546130000	-1.561399000
6	-8.776356000	-3.061201000	-0.683621000
6	-8.351683000	-4.281070000	-0.236817000
6	-7.058907000	-4.529185000	-0.836831000
7	-6.694602000	-3.448348000	-1.630048000
1	-6.728825000	-6.456529000	-0.021233000
1	-1.393417000	-5.869613000	-3.512710000
1	-3.302869000	-0.078233000	-5.455242000
1	-8.706295000	-0.733260000	-2.093840000
8	-0.296431000	2.274889000	2.020044000
6	0.188315000	1.667410000	0.987617000
8	0.987729000	2.211886000	0.125839000
6	-0.183091000	0.244347000	0.746923000
6	-0.960396000	-0.447177000	1.602805000
1	-3.134050000	-2.506381000	-0.786955000
1	0.225490000	-0.189699000	-0.160414000
1	-1.316381000	0.093540000	2.478208000
8	-4.062580000	-2.251551000	-0.960356000
1	-2.264641000	-0.023864000	-10.083810000
1	3.903881000	-0.071771000	-2.710480000
1	-2.888960000	1.833973000	-3.001830000
1	-2.571458000	6.151895000	-1.785646000
1	7.836582000	5.144776000	0.220150000
1	7.314330000	8.776702000	2.548352000
1	-8.860886000	-4.965388000	0.426386000
1	-4.644757000	-7.997792000	-0.483492000
1	-7.740478000	1.116770000	-3.696663000
1	-9.701013000	-2.550486000	-0.456688000
1	-2.395048000	-7.755721000	-1.968204000
1	-0.332388000	-3.985523000	-5.027408000
1	-5.448481000	1.388697000	-5.123494000
1	-1.138189000	-1.529835000	-5.836020000
6	-1.391344000	-1.845118000	1.483615000
6	-2.297177000	-2.364596000	2.432717000
6	-0.947144000	-2.706226000	0.452761000
6	-2.752299000	-3.685175000	2.355225000
1	-2.645102000	-1.718738000	3.233845000
6	-1.399814000	-4.026367000	0.376690000
1	-0.237875000	-2.343948000	-0.283925000
6	-2.308040000	-4.522902000	1.325418000
1	-3.450518000	-4.059211000	3.097727000
1	-1.051245000	-4.669167000	-0.424584000
1	-2.663652000	-5.544971000	1.252265000
1	-4.610815000	-2.476511000	-0.185429000

$^{4}TS2_{DC,beta,S1}$

26	-4.848911000	-2.772488000	-2.644821000
16	-5.694583000	-4.307145000	-4.227731000
1	-6.771271000	-4.761788000	-3.491393000
6	6.628548000	4.292175000	0.335843000
6	6.483746000	2.921623000	1.051353000
6	5.031199000	2.425267000	1.253625000
6	4.331178000	2.112490000	-0.083371000
6	5.023279000	1.182692000	2.166840000
6	6.078664000	5.459673000	1.136962000
8	4.964285000	5.988796000	0.876980000
1	6.098572000	4.279491000	-0.618791000
1	7.032342000	2.173788000	0.461232000
1	6.987592000	2.973669000	2.028581000
1	4.465380000	3.220486000	1.762367000
1	3.297769000	1.787126000	0.076081000
1	4.293441000	2.986011000	-0.743240000
1	4.859960000	1.310049000	-0.615572000
1	3.999667000	0.830063000	2.334411000
1	5.475555000	1.397006000	3.144027000
1	5.588147000	0.359517000	1.709546000
7	6.866274000	5.903028000	2.161969000
6	6.543883000	7.055515000	3.007911000
6	6.426841000	6.715816000	4.514970000
6	5.180893000	5.948602000	4.914514000
6	4.026133000	6.638637000	5.330146000
6	2.878020000	5.944925000	5.733866000
6	2.865191000	4.542171000	5.722704000
6	4.004107000	3.844210000	5.299763000
6	5.150428000	4.543316000	4.902794000
1	7.763506000	5.457971000	2.298892000
1	5.607507000	7.470369000	2.628337000
1	7.322479000	6.158371000	4.821439000
1	6.455284000	7.666821000	5.061989000
1	4.035880000	7.724914000	5.362953000
1	2.008096000	6.494924000	6.079692000
1	1.989566000	4.000996000	6.069243000
1	4.005423000	2.759090000	5.293907000
1	6.034485000	3.992121000	4.597300000
6	2.893721000	0.133127000	-3.703911000
6	1.941318000	1.344830000	-3.611818000
6	0.749717000	1.286193000	-4.583655000
6	2.271888000	-1.185231000	-3.211890000
1	3.232550000	0.014528000	-4.745148000
1	1.565253000	1.432689000	-2.583093000
1	2.517171000	2.260599000	-3.814997000
1	1.096603000	1.243568000	-5.626460000
1	0.127091000	0.401705000	-4.403218000
1	0.110801000	2.168573000	-4.468673000
1	2.991109000	-2.012095000	-3.270135000

1	1.393529000	-1.464115000	-3.805281000
1	1.947393000	-1.091955000	-2.167763000
6	-1.653145000	5.954639000	-2.311291000
6	-0.511332000	5.990093000	-1.263897000
6	0.837555000	5.461565000	-1.785807000
6	1.920598000	5.434607000	-0.692877000
7	1.545463000	4.486684000	0.371620000
6	1.674632000	4.705755000	1.686082000
7	2.489350000	5.684932000	2.148632000
7	0.975905000	3.952749000	2.558532000
6	-1.939722000	4.549974000	-2.828215000
8	-1.384962000	4.145889000	-3.892318000
1	-1.368335000	6.549101000	-3.185056000
1	-0.804837000	5.406624000	-0.382206000
1	-0.387116000	7.028884000	-0.923847000
1	0.712185000	4.455380000	-2.202923000
1	1.194010000	6.093530000	-2.610147000
1	2.891556000	5.156017000	-1.118271000
1	2.036000000	6.428251000	-0.246175000
1	1.158407000	3.565971000	0.072347000
1	3.306091000	5.974972000	1.609821000
1	2.469238000	5.926122000	3.128418000
1	0.218465000	3.316670000	2.235889000
1	1.250806000	3.933365000	3.528346000
7	-2.764403000	3.751834000	-2.103494000
6	-3.041832000	2.351702000	-2.523072000
6	-4.224335000	1.930728000	-1.634306000
6	-4.023962000	2.760294000	-0.346003000
6	-3.483716000	4.112460000	-0.855282000
1	-2.149077000	1.739799000	-2.345014000
1	-5.171011000	2.203154000	-2.118701000
1	-4.242208000	0.852064000	-1.454431000
1	-4.946340000	2.881325000	0.230580000
1	-3.277284000	2.290883000	0.303825000
1	-2.812551000	4.581159000	-0.130613000
1	-4.300406000	4.815009000	-1.076599000
6	-2.166869000	0.416978000	-9.530988000
6	-2.837579000	1.454171000	-8.654794000
6	-4.163334000	1.853707000	-8.900435000
6	-4.791366000	2.798148000	-8.079831000
6	-4.101760000	3.358273000	-6.996224000
6	-2.779911000	2.972112000	-6.735671000
6	-2.158428000	2.028619000	-7.564702000
1	-1.080979000	0.558891000	-9.557718000
1	-2.354976000	-0.601469000	-9.163240000
1	-4.702402000	1.425974000	-9.741752000
1	-5.812050000	3.102902000	-8.293770000
1	-4.584609000	4.097209000	-6.363907000
1	-2.238851000	3.399955000	-5.896024000
1	-1.128920000	1.742301000	-7.364881000

6	-5.530485000	-4.926666000	-0.047513000
6	-4.299415000	-5.050674000	-0.671285000
6	-3.305636000	-6.047073000	-0.339987000
6	-2.276243000	-5.901429000	-1.226155000
6	-2.620782000	-4.808315000	-2.106972000
7	-3.853497000	-4.287010000	-1.739940000
6	-1.836731000	-4.363668000	-3.158241000
6	-2.164931000	-3.313044000	-4.000261000
6	-1.308609000	-2.803925000	-5.047748000
6	-1.945737000	-1.730419000	-5.604739000
6	-3.205456000	-1.575439000	-4.913553000
7	-3.326145000	-2.556236000	-3.941653000
6	-4.134892000	-0.586104000	-5.192743000
6	-5.353531000	-0.451928000	-4.550141000
6	-6.350425000	0.540191000	-4.891303000
6	-7.431035000	0.315931000	-4.086663000
6	-7.106018000	-0.810478000	-3.237377000
7	-5.828296000	-1.258251000	-3.527879000
6	-7.955880000	-1.350294000	-2.286535000
6	-7.638491000	-2.415059000	-1.458327000
6	-8.494495000	-2.922254000	-0.409989000
6	-7.806782000	-3.915513000	0.229129000
6	-6.523075000	-4.035865000	-0.423851000
7	-6.445849000	-3.122829000	-1.467381000
1	-5.740142000	-5.589009000	0.785020000
1	-0.889416000	-4.863873000	-3.325338000
1	-3.896640000	0.121553000	-5.978468000
1	-8.932132000	-0.893768000	-2.166478000
8	-0.857291000	2.162169000	1.531208000
6	-0.357651000	1.622842000	0.504627000
8	0.586259000	1.967705000	-0.270619000
6	-1.252346000	-0.639347000	-0.068322000
6	-1.863692000	-1.194812000	1.016393000
1	-1.832557000	-0.120836000	-0.820949000
1	-0.221199000	-0.843923000	-0.334217000
1	-2.924340000	-0.995940000	1.164335000
8	-4.130079000	-1.607496000	-1.457653000
1	-2.538931000	0.461192000	-10.560482000
1	3.795631000	0.342609000	-3.108968000
1	-3.262191000	2.318173000	-3.592209000
1	-2.553891000	6.405395000	-1.877557000
1	7.690522000	4.470893000	0.119180000
1	7.324600000	7.816445000	2.878362000
1	-8.122605000	-4.515521000	1.070157000
1	-3.402064000	-6.762223000	0.463744000
1	-8.369685000	0.850135000	-4.058703000
1	-9.483983000	-2.546081000	-0.194368000
1	-1.361483000	-6.472149000	-1.290915000
1	-0.343316000	-3.216939000	-5.301436000
1	-6.218310000	1.290904000	-5.656569000

1	-1.607360000	-1.084839000	-6.401225000
6	-1.228953000	-1.989885000	2.068110000
6	-2.029836000	-2.565129000	3.081607000
6	0.166327000	-2.216572000	2.129484000
6	-1.469709000	-3.338824000	4.099766000
1	-3.103409000	-2.397769000	3.058226000
6	0.725111000	-2.990578000	3.147172000
1	0.813468000	-1.773535000	1.379799000
6	-0.086975000	-3.559359000	4.138736000
1	-2.110652000	-3.769496000	4.863877000
1	1.799659000	-3.150036000	3.170854000
1	0.351487000	-4.161039000	4.929075000
1	-3.354579000	-1.935807000	-0.949063000

$^{4}Pr_{DC,beta,S1}$

26	-5.100398000	-2.583924000	-2.236374000
16	-5.808069000	-4.195370000	-3.809943000
1	-7.064620000	-4.430603000	-3.285819000
6	6.681701000	4.656574000	0.576112000
6	6.583452000	3.237122000	1.199126000
6	5.160875000	2.627235000	1.238762000
6	4.614830000	2.334923000	-0.172704000
6	5.162688000	1.341525000	2.090000000
6	5.984933000	5.729258000	1.395152000
8	4.849876000	6.181445000	1.084009000
1	6.228299000	4.665294000	-0.417312000
1	7.237910000	2.568838000	0.621603000
1	6.994122000	3.265731000	2.219861000
1	4.491464000	3.352775000	1.725259000
1	3.596779000	1.933540000	-0.129180000
1	4.578632000	3.235411000	-0.795872000
1	5.247278000	1.596544000	-0.683981000
1	4.156872000	0.911382000	2.147965000
1	5.511204000	1.536380000	3.112723000
1	5.823483000	0.583249000	1.649536000
7	6.661374000	6.174447000	2.495697000
6	6.183372000	7.242198000	3.378384000
6	5.986412000	6.800399000	4.850299000
6	4.788793000	5.905367000	5.105207000
6	3.543441000	6.466122000	5.446929000
6	2.434932000	5.651777000	5.712698000
6	2.554207000	4.256152000	5.634158000
6	3.785764000	3.686533000	5.285387000
6	4.891666000	4.505587000	5.027842000
1	7.581009000	5.794711000	2.674149000
1	5.245238000	7.604172000	2.951801000
1	6.903508000	6.303826000	5.195508000
1	5.888012000	7.714572000	5.449674000
1	3.448641000	7.545603000	5.530101000
1	1.491060000	6.102294000	6.004328000

1	1.706469000	3.620844000	5.873775000
1	3.889188000	2.607847000	5.228900000
1	5.847357000	4.052884000	4.781850000
6	3.104152000	0.292298000	-3.836337000
6	2.132085000	1.481588000	-3.991335000
6	0.842949000	1.152312000	-4.762400000
6	2.560032000	-0.832780000	-2.938063000
1	3.353743000	-0.112500000	-4.829906000
1	1.859998000	1.844681000	-2.991855000
1	2.653304000	2.303567000	-4.504636000
1	1.069223000	0.827902000	-5.788803000
1	0.280220000	0.350062000	-4.270275000
1	0.190477000	2.031136000	-4.806333000
1	3.310729000	-1.618984000	-2.785268000
1	1.670515000	-1.303828000	-3.372222000
1	2.271033000	-0.431159000	-1.959212000
6	-1.447963000	6.014350000	-2.596474000
6	-0.353902000	6.101553000	-1.501990000
6	1.021100000	5.568028000	-1.943890000
6	2.021249000	5.490085000	-0.776741000
7	1.593386000	4.466880000	0.194292000
6	1.614879000	4.596887000	1.526048000
7	2.318422000	5.593833000	2.116294000
7	0.919522000	3.732086000	2.292828000
6	-1.713562000	4.582303000	-3.048141000
8	-1.141784000	4.131951000	-4.084005000
1	-1.123721000	6.561847000	-3.486879000
1	-0.680342000	5.544293000	-0.614815000
1	-0.253861000	7.152135000	-1.191990000
1	0.913447000	4.577031000	-2.401131000
1	1.443836000	6.219948000	-2.719984000
1	3.026363000	5.254976000	-1.145258000
1	2.083384000	6.455255000	-0.262819000
1	1.288252000	3.547313000	-0.191421000
1	3.147645000	5.980175000	1.664090000
1	2.214060000	5.755827000	3.107027000
1	0.265605000	3.036790000	1.881081000
1	1.129256000	3.668342000	3.276993000
7	-2.533419000	3.812154000	-2.288325000
6	-2.777859000	2.380543000	-2.621029000
6	-3.964062000	1.995324000	-1.721285000
6	-3.788021000	2.907175000	-0.487370000
6	-3.295192000	4.238706000	-1.087494000
1	-1.876689000	1.799410000	-2.390410000
1	-4.909568000	2.223822000	-2.230401000
1	-3.975598000	0.931606000	-1.468716000
1	-4.710479000	3.032626000	0.088079000
1	-3.019323000	2.496500000	0.177381000
1	-2.664218000	4.798187000	-0.391674000
1	-4.137896000	4.882481000	-1.380049000

6	-2.259789000	-0.100260000	-9.165062000
6	-2.836126000	1.144604000	-8.524090000
6	-4.077771000	1.657638000	-8.940753000
6	-4.623034000	2.795501000	-8.335236000
6	-3.934010000	3.440455000	-7.299024000
6	-2.695135000	2.944910000	-6.872117000
6	-2.155290000	1.806430000	-7.486565000
1	-1.174531000	-0.152800000	-9.028175000
1	-2.690678000	-1.011819000	-8.728254000
1	-4.616505000	1.164171000	-9.745666000
1	-5.579654000	3.181113000	-8.676470000
1	-4.354630000	4.326116000	-6.832365000
1	-2.150675000	3.433699000	-6.068773000
1	-1.187326000	1.435992000	-7.159233000
6	-6.525287000	-4.396558000	0.325713000
6	-5.218217000	-4.700042000	-0.020823000
6	-4.428189000	-5.748156000	0.585293000
6	-3.224681000	-5.773314000	-0.062663000
6	-3.260720000	-4.740463000	-1.072423000
7	-4.480994000	-4.081618000	-1.019769000
6	-2.234879000	-4.473744000	-1.963799000
6	-2.281139000	-3.509920000	-2.957518000
6	-1.192025000	-3.210578000	-3.859727000
6	-1.601558000	-2.181850000	-4.660284000
6	-2.947374000	-1.837720000	-4.259325000
7	-3.349905000	-2.666674000	-3.224767000
6	-3.697332000	-0.818007000	-4.824167000
6	-4.983223000	-0.481700000	-4.438150000
6	-5.774665000	0.561631000	-5.056201000
6	-6.989411000	0.563132000	-4.432207000
6	-6.956339000	-0.475776000	-3.424342000
7	-5.718276000	-1.095518000	-3.436004000
6	-8.016451000	-0.800887000	-2.595548000
6	-7.990557000	-1.801376000	-1.637192000
6	-9.082710000	-2.105530000	-0.740967000
6	-8.662237000	-3.111685000	0.082834000
6	-7.307436000	-3.438936000	-0.298908000
7	-6.918961000	-2.639160000	-1.366257000
1	-6.972448000	-4.957246000	1.139160000
1	-1.325637000	-5.056272000	-1.871078000
1	-3.247445000	-0.242169000	-5.625301000
1	-8.930372000	-0.226344000	-2.698025000
8	-0.612103000	1.750481000	1.093322000
6	-0.020306000	1.390248000	0.026927000
8	0.890092000	1.944162000	-0.661723000
6	-2.707996000	-1.059279000	1.855895000
6	-2.317846000	-2.345922000	1.934643000
1	-3.725021000	-0.777061000	2.113145000
1	-2.042897000	-0.247966000	1.571046000
1	-3.039711000	-3.089903000	2.273051000

8	-4.473338000	-1.364782000	-1.055335000
1	-2.466256000	-0.130161000	-10.240946000
1	4.049031000	0.661735000	-3.409218000
1	-2.984662000	2.276648000	-3.688359000
1	-2.366547000	6.487097000	-2.228049000
1	7.740786000	4.922240000	0.456735000
1	6.904713000	8.069343000	3.352718000
1	-9.204031000	-3.592858000	0.884140000
1	-4.769728000	-6.381435000	1.391292000
1	-7.842233000	1.197288000	-4.626604000
1	-10.036643000	-1.598498000	-0.747345000
1	-2.379907000	-6.422401000	0.113125000
1	-0.240972000	-3.722361000	-3.858663000
1	-5.428585000	1.192425000	-5.862248000
1	-1.052894000	-1.682044000	-5.444431000
6	-0.977953000	-2.885881000	1.634957000
6	-0.651108000	-4.186745000	2.070740000
6	-0.003706000	-2.156605000	0.920497000
6	0.608470000	-4.738672000	1.819162000
1	-1.394812000	-4.763363000	2.614034000
6	1.252868000	-2.710728000	0.666169000
1	-0.231006000	-1.161964000	0.548649000
6	1.567245000	-4.001356000	1.114631000
1	0.840009000	-5.740183000	2.170279000
1	1.983946000	-2.135726000	0.105973000
1	2.544824000	-4.428177000	0.911160000
1	-3.867696000	-1.692696000	-0.351954000

Full Substrate (S1)

8	-1.072147000	1.620148000	1.618017000
6	-0.632921000	1.324598000	0.441572000
8	-0.044122000	2.085219000	-0.396749000
6	-0.868848000	-0.145887000	-0.052808000
6	-1.483180000	-1.093578000	1.003049000
1	-1.542288000	-0.082323000	-0.921311000
1	0.085256000	-0.534350000	-0.433274000
1	-2.203029000	-0.486823000	1.566980000
6	-0.485117000	-1.697748000	1.972855000
6	0.191245000	-0.882427000	2.905378000
6	-0.207395000	-3.076158000	1.969050000
6	1.110550000	-1.440668000	3.798952000
1	-0.030157000	0.183167000	2.887685000
6	0.714003000	-3.635198000	2.864972000
1	-0.721554000	-3.717151000	1.255595000
6	1.377682000	-2.817727000	3.786118000
1	1.623575000	-0.797534000	4.510193000
1	0.911154000	-4.704860000	2.842312000
1	2.093759000	-3.246044000	4.483630000

1	-2.028747000	-1.911420000	0.508318000

Substrate_S1_C2_alpha (Phenyl_Propionic_acid_non_fix remove_alpha)

8	-1.034698000	1.678937000	1.605333000
6	-0.649794000	1.361896000	0.402710000
8	-0.233629000	2.157274000	-0.519832000
6	-0.693905000	-0.085312000	0.060825000
6	-1.367246000	-1.081229000	0.966498000
1	-0.166870000	-0.430262000	-0.827840000
1	-2.132782000	-0.527525000	1.526199000
6	-0.417201000	-1.722343000	1.977877000
6	0.235172000	-0.906844000	2.925571000
6	-0.170420000	-3.104328000	1.992457000
6	1.101898000	-1.473443000	3.864081000
1	0.034507000	0.163413000	2.882922000
6	0.702460000	-3.670850000	2.932406000
1	-0.667373000	-3.740915000	1.263099000
6	1.341050000	-2.856629000	3.873707000
1	1.597103000	-0.834413000	4.591346000
1	0.881724000	-4.743832000	2.927375000
1	2.018671000	-3.291837000	4.604428000
1	-1.855771000	-1.880168000	0.387347000

Substrate_S1_C3_beta (Phenyl_Propionic_acid_non_fix remove_beta)

8	-0.620563000	1.758773000	1.656007000
6	-1.285028000	1.303022000	0.665547000
8	-2.393585000	1.673005000	0.171057000
6	-0.578528000	0.046313000	-0.089379000
6	-0.834468000	-1.265047000	0.543781000
1	-0.961464000	0.057427000	-1.113606000
1	0.492082000	0.287680000	-0.097396000
6	-0.167615000	-1.781608000	1.688845000
6	0.742448000	-0.992519000	2.465045000
6	-0.376557000	-3.133529000	2.108319000
6	1.411520000	-1.546078000	3.552584000
1	0.827048000	0.065139000	2.231511000
6	0.303662000	-3.672056000	3.195785000
1	-1.079028000	-3.748090000	1.548657000
6	1.214451000	-2.888368000	3.926546000
1	2.087013000	-0.921585000	4.133398000
1	0.127337000	-4.706894000	3.482990000
1	1.745167000	-3.309213000	4.776667000
1	-1.624169000	-1.883017000	0.116088000

Model-S2 Data

$^{2}Re_{S2}$

26	-5.729708000	-2.246379000	-2.764438000
16	-6.738951000	-3.636785000	-4.659268000
1	-7.327950000	-2.605293000	-5.366764000
6	5.499283000	5.316562000	0.486911000
6	5.717428000	3.817377000	0.850058000
6	4.420197000	2.985687000	1.008514000
6	3.736947000	2.722243000	-0.348210000
6	4.726722000	1.653284000	1.721564000
6	5.102638000	6.156589000	1.685530000
8	3.911607000	6.241117000	2.097299000
1	4.710576000	5.408056000	-0.265254000
1	6.331603000	3.361770000	0.060264000
1	6.305856000	3.761078000	1.776626000
1	3.726863000	3.559133000	1.639787000
1	2.769538000	2.226223000	-0.216121000
1	3.552834000	3.648889000	-0.905850000
1	4.368450000	2.077620000	-0.974699000
1	3.821850000	1.042508000	1.815559000
1	5.137404000	1.823020000	2.724926000
1	5.460442000	1.067491000	1.151301000
7	6.121335000	6.796103000	2.327588000
6	5.944256000	7.575253000	3.555999000
6	6.849803000	7.104196000	4.708870000
6	6.713289000	5.647762000	5.129307000
6	5.500601000	4.943415000	5.035757000
6	5.409875000	3.616061000	5.473278000
6	6.526548000	2.969884000	6.014717000
6	7.740304000	3.660026000	6.110732000
6	7.829959000	4.983361000	5.667837000
1	7.057986000	6.693209000	1.963317000
1	4.887060000	7.499643000	3.817022000
1	7.899296000	7.298537000	4.445267000
1	6.638754000	7.758817000	5.568798000
1	4.625498000	5.414802000	4.601021000
1	4.466300000	3.086204000	5.385895000
1	6.453339000	1.941034000	6.351878000
1	8.615809000	3.168393000	6.523312000
1	8.777748000	5.510722000	5.741824000
6	2.027764000	1.902362000	-4.114089000
6	0.836462000	2.712058000	-3.558769000
6	-0.460235000	2.576470000	-4.375228000
6	1.845049000	0.377211000	-4.024879000
1	2.205992000	2.190719000	-5.162613000
1	0.645817000	2.410832000	-2.519098000
1	1.120121000	3.774969000	-3.528992000
1	-0.297735000	2.865648000	-5.423741000
1	-0.831483000	1.543479000	-4.369283000

1	-1.245268000	3.218277000	-3.961055000
1	2.729626000	-0.152372000	-4.401211000
1	0.981726000	0.041516000	-4.612434000
1	1.684755000	0.067388000	-2.984840000
6	-2.724891000	6.262860000	-0.768229000
6	-1.468942000	6.154084000	0.134126000
6	-0.183745000	5.771917000	-0.621737000
6	0.999182000	5.501307000	0.326263000
7	0.730547000	4.320102000	1.162283000
6	0.827221000	4.271212000	2.493988000
7	1.525088000	5.217379000	3.182232000
7	0.214025000	3.276145000	3.163284000
6	-3.015759000	4.970772000	-1.522353000
8	-2.593456000	4.815848000	-2.702461000
1	-2.565200000	7.031810000	-1.530498000
1	-1.647258000	5.410452000	0.920939000
1	-1.321970000	7.118230000	0.643011000
1	-0.359547000	4.884867000	-1.240808000
1	0.100095000	6.578474000	-1.311639000
1	1.913586000	5.336854000	-0.254869000
1	1.176956000	6.365734000	0.973540000
1	0.409050000	3.437579000	0.669782000
1	2.306259000	5.709274000	2.748981000
1	1.398567000	5.300662000	4.177374000
1	-0.369971000	2.556705000	2.653570000
1	0.482192000	3.081171000	4.115362000
7	-3.694068000	3.985595000	-0.873834000
6	-3.935476000	2.672914000	-1.528322000
6	-4.936277000	1.979738000	-0.588550000
6	-4.579402000	2.556271000	0.799489000
6	-4.251904000	4.032948000	0.499803000
1	-2.987495000	2.124830000	-1.599570000
1	-5.962759000	2.260557000	-0.860301000
1	-4.870541000	0.889829000	-0.636518000
1	-5.389375000	2.453304000	1.528140000
1	-3.690323000	2.057414000	1.203500000
1	-3.531932000	4.447640000	1.209927000
1	-5.158249000	4.656383000	0.525129000
6	-1.546632000	-3.564099000	-8.134663000
6	-1.526009000	-2.051302000	-8.158537000
6	-2.640405000	-1.320241000	-8.604371000
6	-2.634049000	0.079908000	-8.595334000
6	-1.509281000	0.776404000	-8.136751000
6	-0.389209000	0.060691000	-7.693193000
6	-0.398937000	-1.338987000	-7.708508000
1	-0.563561000	-3.982756000	-8.378102000
1	-1.824908000	-3.938492000	-7.140102000
1	-3.518065000	-1.852779000	-8.959847000
1	-3.504551000	0.624093000	-8.949169000
1	-1.500048000	1.861695000	-8.130410000

1	0.489426000	0.591447000	-7.340480000
1	0.479015000	-1.886422000	-7.374982000
6	-8.205919000	-3.849640000	-0.993463000
6	-6.960127000	-4.458392000	-1.044806000
6	-6.620837000	-5.695069000	-0.372386000
6	-5.318745000	-5.970839000	-0.674270000
6	-4.847815000	-4.903328000	-1.530741000
7	-5.865722000	-3.987018000	-1.749496000
6	-3.569113000	-4.812563000	-2.054327000
6	-3.108279000	-3.791758000	-2.876143000
6	-1.771287000	-3.721853000	-3.424673000
6	-1.708791000	-2.583562000	-4.178913000
6	-3.007221000	-1.950943000	-4.093967000
7	-3.851603000	-2.703693000	-3.296398000
6	-3.340950000	-0.761255000	-4.726847000
6	-4.578926000	-0.141396000	-4.657162000
6	-4.910480000	1.107307000	-5.309460000
6	-6.214623000	1.379482000	-5.008251000
6	-6.690781000	0.297846000	-4.171718000
7	-5.678920000	-0.623958000	-3.965594000
6	-7.982429000	0.196558000	-3.669250000
6	-8.458655000	-0.839843000	-2.884810000
6	-9.802261000	-0.930889000	-2.357335000
6	-9.863078000	-2.067307000	-1.603259000
6	-8.558658000	-2.689496000	-1.660244000
7	-7.711112000	-1.937065000	-2.466739000
1	-8.969730000	-4.329543000	-0.390749000
1	-2.868768000	-5.601696000	-1.801927000
1	-2.573612000	-0.279800000	-5.323131000
1	-8.674850000	0.995491000	-3.913166000
8	-1.022913000	1.303892000	1.838785000
6	-0.545085000	1.101705000	0.654670000
8	0.070917000	2.005322000	-0.031998000
6	-0.654546000	-0.295200000	0.025951000
6	-2.007952000	-1.020060000	0.298615000
6	-2.252929000	-1.522854000	1.729120000
1	-0.598481000	-0.124514000	-1.055445000
1	-2.820436000	-0.342933000	0.008167000
1	-2.063804000	-1.870246000	-0.395431000
1	-2.226085000	-0.691270000	2.436878000
1	-1.496226000	-2.256879000	2.030748000
8	-5.302203000	-1.369475000	-1.419253000
1	-2.272478000	-3.965836000	-8.849043000
1	2.936493000	2.180206000	-3.559844000
1	-4.310902000	2.823964000	-2.543338000
1	-3.584307000	6.567524000	-0.158318000
1	6.418542000	5.721359000	0.044649000
1	6.151143000	8.634127000	3.353055000
1	-10.703898000	-2.459716000	-1.050225000
1	-7.302601000	-6.265523000	0.241180000

1	-6.810699000	2.226480000	-5.314904000
1	-10.583239000	-0.208158000	-2.543529000
1	-4.719549000	-6.811430000	-0.356394000
1	-0.996404000	-4.452815000	-3.246029000
1	-4.221834000	1.683503000	-5.908727000
1	-0.877667000	-2.192899000	-4.746766000
6	0.552176000	-1.157246000	0.402362000
6	1.094622000	-2.042528000	-0.546229000
6	1.126549000	-1.121792000	1.686322000
6	2.177776000	-2.868735000	-0.225904000
1	0.660506000	-2.081824000	-1.541833000
6	2.211442000	-1.945994000	2.007709000
1	0.713554000	-0.444916000	2.426435000
6	2.742170000	-2.823073000	1.053994000
1	2.583839000	-3.541652000	-0.975922000
1	2.640070000	-1.905724000	3.005273000
1	3.584663000	-3.460853000	1.304414000
1	-3.234617000	-2.009051000	1.787377000

$^{4}Re_{S2}$

26	-5.749869000	-2.234428000	-2.743484000
16	-6.752732000	-3.635226000	-4.657487000
1	-7.378416000	-2.609339000	-5.341651000
6	5.526500000	5.282143000	0.486305000
6	5.734620000	3.781777000	0.850520000
6	4.431871000	2.958693000	1.008716000
6	3.747358000	2.699519000	-0.348192000
6	4.729400000	1.624421000	1.722090000
6	5.132644000	6.125089000	1.683753000
8	3.941494000	6.215615000	2.093942000
1	4.739818000	5.378136000	-0.267415000
1	6.346253000	3.321716000	0.061336000
1	6.322146000	3.722242000	1.777448000
1	3.742156000	3.536798000	1.639688000
1	2.776590000	2.210064000	-0.216374000
1	3.569612000	3.627191000	-0.906208000
1	4.374717000	2.050461000	-0.974277000
1	3.820508000	1.019637000	1.816141000
1	5.141023000	1.791634000	2.725486000
1	5.459307000	1.033656000	1.152072000
7	6.153577000	6.760344000	2.326492000
6	5.978054000	7.542063000	3.553506000
6	6.880674000	7.069452000	4.708103000
6	6.740544000	5.613308000	5.128094000
6	5.524785000	4.913671000	5.038323000
6	5.430343000	3.586516000	5.475479000
6	6.546428000	2.935694000	6.012654000
6	7.763169000	3.621017000	6.104771000
6	7.856441000	4.944309000	5.662353000

1	7.090136000	6.653264000	1.963215000
1	4.920295000	7.470562000	3.813369000
1	7.930999000	7.261782000	4.446422000
1	6.669237000	7.724530000	5.567590000
1	4.650124000	5.388822000	4.606753000
1	4.484347000	3.060469000	5.391298000
1	6.470460000	1.906973000	6.349555000
1	8.638201000	3.125736000	6.513971000
1	8.806507000	5.467954000	5.733414000
6	2.043669000	1.875506000	-4.120458000
6	0.862674000	2.696155000	-3.559075000
6	-0.434155000	2.585996000	-4.379151000
6	1.839858000	0.352224000	-4.046587000
1	2.227159000	2.171484000	-5.165920000
1	0.666280000	2.388648000	-2.522309000
1	1.160998000	3.754732000	-3.519085000
1	-0.265584000	2.883902000	-5.424266000
1	-0.819093000	1.558033000	-4.384957000
1	-1.212031000	3.233532000	-3.960416000
1	2.716208000	-0.185729000	-4.430181000
1	0.970659000	0.034833000	-4.635700000
1	1.677333000	0.033834000	-3.009478000
6	-2.694238000	6.275764000	-0.774214000
6	-1.440708000	6.160631000	0.130685000
6	-0.155883000	5.771802000	-0.622441000
6	1.023647000	5.495827000	0.328225000
7	0.747607000	4.315778000	1.163344000
6	0.846148000	4.264824000	2.494824000
7	1.549525000	5.206958000	3.183066000
7	0.229018000	3.272119000	3.163977000
6	-2.989849000	4.985219000	-1.529078000
8	-2.565714000	4.828293000	-2.708242000
1	-2.529165000	7.043976000	-1.536091000
1	-1.624348000	5.417994000	0.917221000
1	-1.289860000	7.124074000	0.639757000
1	-0.334810000	4.885340000	-1.241488000
1	0.133339000	6.576637000	-1.312101000
1	1.938810000	5.327729000	-0.250684000
1	1.203473000	6.359381000	0.976148000
1	0.420853000	3.435449000	0.670303000
1	2.333019000	5.694772000	2.749449000
1	1.425229000	5.289287000	4.178573000
1	-0.358072000	2.554996000	2.654350000
1	0.497299000	3.075263000	4.115641000
7	-3.674382000	4.003255000	-0.882209000
6	-3.919731000	2.691522000	-1.537105000
6	-4.924477000	2.001935000	-0.598830000
6	-4.567954000	2.577449000	0.789737000
6	-4.234695000	4.052910000	0.490277000
1	-2.973633000	2.140035000	-1.606975000

1	-5.949505000	2.286478000	-0.872330000
1	-4.862005000	0.911918000	-0.647190000
1	-5.379544000	2.477628000	1.517041000
1	-3.681487000	2.075355000	1.195477000
1	-3.514746000	4.465429000	1.201678000
1	-5.138993000	4.679410000	0.513692000
6	-1.562611000	-3.499132000	-8.142972000
6	-1.569448000	-1.986226000	-8.168553000
6	-2.698062000	-1.276104000	-8.612534000
6	-2.717169000	0.123916000	-8.605169000
6	-1.604096000	0.841300000	-8.150164000
6	-0.470056000	0.146637000	-7.708668000
6	-0.454362000	-1.253022000	-7.722179000
1	-0.571467000	-3.900016000	-8.383490000
1	-1.836579000	-3.877490000	-7.148699000
1	-3.566738000	-1.825002000	-8.965177000
1	-3.598314000	0.651709000	-8.957491000
1	-1.614409000	1.926608000	-8.145443000
1	0.399793000	0.693905000	-7.359520000
1	0.434367000	-1.783949000	-7.390546000
6	-8.212923000	-3.856446000	-0.976244000
6	-6.966651000	-4.462714000	-1.038688000
6	-6.619606000	-5.699990000	-0.372759000
6	-5.317284000	-5.968545000	-0.680566000
6	-4.853846000	-4.896253000	-1.534476000
7	-5.877902000	-3.984037000	-1.747669000
6	-3.575001000	-4.798359000	-2.056147000
6	-3.116982000	-3.773470000	-2.874172000
6	-1.780545000	-3.697541000	-3.425502000
6	-1.726674000	-2.561732000	-4.183354000
6	-3.029508000	-1.936523000	-4.096768000
7	-3.866360000	-2.690814000	-3.294107000
6	-3.374745000	-0.752886000	-4.734057000
6	-4.614806000	-0.137997000	-4.656406000
6	-4.951344000	1.111577000	-5.303619000
6	-6.251706000	1.386257000	-4.987766000
6	-6.720685000	0.304602000	-4.148240000
7	-5.709401000	-0.620420000	-3.955036000
6	-8.007040000	0.204014000	-3.632223000
6	-8.475640000	-0.834861000	-2.847141000
6	-9.818681000	-0.934287000	-2.320107000
6	-9.875366000	-2.075336000	-1.573105000
6	-8.568616000	-2.691920000	-1.633137000
7	-7.721966000	-1.929865000	-2.432149000
1	-8.973747000	-4.341998000	-0.374370000
1	-2.872295000	-5.585829000	-1.805107000
1	-2.614376000	-0.269307000	-5.337524000
1	-8.702133000	1.002730000	-3.869007000
8	-1.015464000	1.304265000	1.840670000
6	-0.540556000	1.102173000	0.655272000

8	0.075431000	2.005341000	-0.031975000
6	-0.654365000	-0.293803000	0.025631000
6	-2.013631000	-1.010608000	0.290448000
6	-2.270363000	-1.511111000	1.719636000
1	-0.591505000	-0.123007000	-1.055344000
1	-2.820341000	-0.329071000	-0.005641000
1	-2.070100000	-1.860909000	-0.403351000
1	-2.243343000	-0.679396000	2.427228000
1	-1.519436000	-2.248953000	2.026394000
8	-5.263442000	-1.372227000	-1.419165000
1	-2.279250000	-3.914841000	-8.858669000
1	2.955399000	2.135436000	-3.562490000
1	-4.293321000	2.843817000	-2.552623000
1	-3.553398000	6.584696000	-0.166091000
1	6.449117000	5.681018000	0.045669000
1	6.188983000	8.599891000	3.349224000
1	-10.715154000	-2.475466000	-1.024096000
1	-7.296249000	-6.275431000	0.241790000
1	-6.849218000	2.235071000	-5.286487000
1	-10.602811000	-0.214377000	-2.503878000
1	-4.712875000	-6.807210000	-0.367502000
1	-1.001457000	-4.424037000	-3.246989000
1	-4.267950000	1.686995000	-5.909617000
1	-0.899352000	-2.167720000	-4.754426000
6	0.545025000	-1.163561000	0.407694000
6	1.084227000	-2.053624000	-0.538391000
6	1.115558000	-1.130863000	1.693365000
6	2.160639000	-2.886907000	-0.214082000
1	0.652426000	-2.090997000	-1.535075000
6	2.193789000	-1.962327000	2.018798000
1	0.704773000	-0.450501000	2.431490000
6	2.721433000	-2.843851000	1.067548000
1	2.564346000	-3.563400000	-0.962160000
1	2.619521000	-1.924478000	3.017701000
1	3.558717000	-3.487224000	1.321133000
1	-3.254985000	-1.991980000	1.771586000

$^{4}TS1_{alpha,HA,S2}$

26	-2.673564000	-1.941888000	-1.738259000
16	-3.519387000	-2.554278000	-3.929065000
1	-3.353053000	-1.327755000	-4.542928000
6	5.774509000	5.004610000	0.162459000
6	5.936667000	3.910510000	-0.924493000
6	4.653428000	3.556165000	-1.712834000
6	4.069751000	4.774487000	-2.454932000
6	4.943871000	2.402914000	-2.693645000
6	4.757872000	4.654259000	1.237202000
8	3.650890000	5.260916000	1.317882000
1	5.452637000	5.947335000	-0.284486000

1	6.706677000	4.251062000	-1.631901000
1	6.326196000	2.992052000	-0.461982000
1	3.901492000	3.197930000	-0.993666000
1	3.192266000	4.481503000	-3.043535000
1	3.756950000	5.563462000	-1.762733000
1	4.807664000	5.199139000	-3.149314000
1	4.037623000	2.120603000	-3.241784000
1	5.302852000	1.513261000	-2.163711000
1	5.704741000	2.696830000	-3.429227000
7	5.110496000	3.656233000	2.087392000
6	4.253492000	3.123959000	3.153677000
6	4.402608000	1.595133000	3.297594000
6	3.922702000	0.808392000	2.091363000
6	2.549240000	0.720430000	1.801370000
6	2.087970000	-0.000982000	0.694598000
6	3.007497000	-0.647064000	-0.143502000
6	4.377481000	-0.569491000	0.132488000
6	4.831279000	0.153775000	1.243891000
1	6.006424000	3.208557000	1.950473000
1	3.222264000	3.383535000	2.906190000
1	5.453911000	1.352735000	3.510259000
1	3.835896000	1.298380000	4.190273000
1	1.821860000	1.208784000	2.443345000
1	1.019111000	-0.059136000	0.513446000
1	2.650614000	-1.216004000	-0.995554000
1	5.092283000	-1.080979000	-0.505816000
1	5.896035000	0.191291000	1.464856000
6	-3.189135000	7.089654000	-7.301548000
6	-2.977676000	6.450837000	-5.912115000
6	-4.106237000	5.502959000	-5.471425000
6	-3.160104000	6.083656000	-8.464720000
1	-4.149767000	7.626520000	-7.305787000
1	-2.020976000	5.906005000	-5.911346000
1	-2.873528000	7.254409000	-5.167782000
1	-5.073010000	6.024201000	-5.469589000
1	-4.195592000	4.642515000	-6.144866000
1	-3.926607000	5.118575000	-4.461408000
1	-3.274600000	6.591563000	-9.429823000
1	-3.965850000	5.345626000	-8.381602000
1	-2.208501000	5.536725000	-8.485260000
6	-1.938677000	6.325862000	-1.489485000
6	-0.635577000	5.903119000	-0.758485000
6	-0.113301000	4.506960000	-1.152619000
6	0.912193000	3.940059000	-0.147782000
7	0.266330000	3.567934000	1.122512000
6	0.181824000	4.313629000	2.228046000
7	1.066397000	5.311697000	2.492644000
7	-0.814338000	4.050295000	3.101531000
6	-3.090131000	5.374804000	-1.193660000
8	-3.386950000	4.454545000	-2.011695000

1	-1.783550000	6.304187000	-2.572188000
1	-0.814346000	5.925103000	0.323042000
1	0.139775000	6.655518000	-0.957971000
1	-0.945674000	3.798754000	-1.233915000
1	0.356848000	4.554816000	-2.145319000
1	1.357816000	3.025474000	-0.550912000
1	1.724998000	4.649558000	0.026402000
1	-0.231695000	2.634602000	1.161605000
1	1.990150000	5.345754000	2.052948000
1	0.850803000	5.992490000	3.202496000
1	-1.580326000	3.379986000	2.830487000
1	-0.742332000	4.356972000	4.059270000
7	-3.739825000	5.495225000	-0.006289000
6	-4.747448000	4.485252000	0.422584000
6	-5.392673000	5.118956000	1.667338000
6	-4.269074000	6.005136000	2.248743000
6	-3.571168000	6.572787000	0.997808000
1	-4.235297000	3.548222000	0.668248000
1	-6.254859000	5.736129000	1.384244000
1	-5.735480000	4.360321000	2.375531000
1	-4.639909000	6.795192000	2.908904000
1	-3.563284000	5.384771000	2.812348000
1	-2.516231000	6.793030000	1.177356000
1	-4.059842000	7.495676000	0.653178000
6	-0.399130000	-3.566003000	-6.763951000
6	1.069623000	-3.445303000	-6.425653000
6	1.755354000	-2.229742000	-6.599310000
6	3.105478000	-2.106151000	-6.250063000
6	3.799451000	-3.201340000	-5.720859000
6	3.131513000	-4.420468000	-5.549428000
6	1.781594000	-4.538866000	-5.900270000
1	-0.651547000	-4.579283000	-7.094804000
1	-1.027866000	-3.343634000	-5.890519000
1	1.224251000	-1.375500000	-7.009787000
1	3.616803000	-1.159428000	-6.399641000
1	4.848302000	-3.109473000	-5.455700000
1	3.662871000	-5.280152000	-5.151235000
1	1.270401000	-5.487881000	-5.764585000
6	-6.045752000	-1.714338000	-1.057910000
6	-5.423579000	-2.931239000	-0.825984000
6	-6.077431000	-4.093640000	-0.263889000
6	-5.126043000	-5.065193000	-0.130256000
6	-3.882425000	-4.512109000	-0.621993000
7	-4.081660000	-3.211479000	-1.044089000
6	-2.679525000	-5.202073000	-0.691344000
6	-1.512998000	-4.703938000	-1.246002000
6	-0.300325000	-5.471965000	-1.439191000
6	0.582567000	-4.669931000	-2.101986000
6	-0.069842000	-3.392813000	-2.303213000
7	-1.349502000	-3.429632000	-1.769285000

6	0.518547000	-2.298815000	-2.914696000
6	-0.070451000	-1.046774000	-3.036961000
6	0.589758000	0.126011000	-3.568999000
6	-0.287267000	1.167728000	-3.449230000
6	-1.500326000	0.640196000	-2.862479000
7	-1.348194000	-0.719154000	-2.624221000
6	-2.647873000	1.382691000	-2.627219000
6	-3.850579000	0.855216000	-2.176293000
6	-5.084196000	1.606845000	-2.066703000
6	-6.051220000	0.725944000	-1.671173000
6	-5.414417000	-0.566301000	-1.514527000
7	-4.071558000	-0.466493000	-1.833063000
1	-7.105958000	-1.647011000	-0.837403000
1	-2.664890000	-6.222511000	-0.323844000
1	1.521945000	-2.422362000	-3.307197000
1	-2.627088000	2.447351000	-2.832652000
8	-2.667154000	2.204932000	2.351246000
6	-2.067155000	1.143562000	1.916161000
8	-0.866824000	1.160811000	1.440672000
6	-2.813859000	-0.183833000	1.988172000
6	-4.342709000	-0.037835000	1.964068000
6	-4.979829000	0.363366000	3.312091000
1	-2.522542000	-0.733485000	0.828883000
1	-4.593589000	0.720212000	1.215208000
1	-4.790854000	-0.974437000	1.610104000
1	-4.581056000	1.329123000	3.631594000
1	-4.770038000	-0.377266000	4.091718000
8	-2.194436000	-1.440222000	-0.158760000
1	-0.684977000	-2.868607000	-7.558743000
1	-2.411357000	7.849590000	-7.466646000
1	-5.455529000	4.294561000	-0.386659000
1	-2.196056000	7.354941000	-1.209954000
1	6.750290000	5.183997000	0.634806000
1	4.500518000	3.612600000	4.106700000
1	-7.099205000	0.916316000	-1.489209000
1	-7.125751000	-4.141687000	-0.006847000
1	-0.151777000	2.199063000	-3.740971000
1	-5.152853000	2.664774000	-2.267789000
1	-5.240981000	-6.068322000	0.254141000
1	-0.167223000	-6.494614000	-1.117009000
1	1.590909000	0.128803000	-3.974666000
1	1.584104000	-4.899748000	-2.434340000
6	-2.242422000	-1.210396000	2.914433000
6	-2.994875000	-2.359315000	3.264218000
6	-0.930098000	-1.108902000	3.435548000
6	-2.472647000	-3.337659000	4.110871000
1	-3.991439000	-2.493319000	2.860703000
6	-0.412625000	-2.087918000	4.284689000
1	-0.318890000	-0.264242000	3.146712000
6	-1.179226000	-3.206989000	4.632917000

1	-3.074824000	-4.206290000	4.360410000
1	0.597603000	-1.981383000	4.669174000
1	-0.773247000	-3.969282000	5.290908000
1	-6.069610000	0.445636000	3.207962000

^{4}IM1$_{alpha,S2}$

26	-1.717902000	-1.389485000	-1.738322000
16	-2.850698000	-2.460295000	-3.540711000
1	-2.978387000	-1.353449000	-4.357209000
6	5.366693000	3.882315000	-0.133473000
6	5.487736000	2.576512000	-0.956174000
6	4.197893000	2.125119000	-1.682679000
6	3.711598000	3.163415000	-2.712773000
6	4.421215000	0.753388000	-2.348651000
6	4.285742000	3.860665000	0.935756000
8	3.389542000	4.756156000	0.974650000
1	5.140506000	4.730844000	-0.782615000
1	6.285213000	2.719539000	-1.699755000
1	5.829293000	1.756499000	-0.306827000
1	3.408465000	1.997806000	-0.925839000
1	2.806002000	2.805734000	-3.216396000
1	3.473629000	4.126418000	-2.247016000
1	4.477318000	3.337143000	-3.481315000
1	3.503178000	0.403129000	-2.832361000
1	4.715279000	-0.002024000	-1.610638000
1	5.208086000	0.813256000	-3.113110000
7	4.336434000	2.856171000	1.842699000
6	3.327598000	2.667024000	2.895304000
6	3.487831000	1.289712000	3.565765000
6	3.374337000	0.138290000	2.580676000
6	2.182370000	-0.072738000	1.862462000
6	2.083310000	-1.098715000	0.916833000
6	3.184312000	-1.937559000	0.683255000
6	4.372275000	-1.744903000	1.396442000
6	4.466915000	-0.710520000	2.338333000
1	5.012641000	2.115075000	1.715709000
1	2.333091000	2.745672000	2.445606000
1	4.454359000	1.243482000	4.085245000
1	2.713921000	1.209919000	4.340369000
1	1.321740000	0.570091000	2.029935000
1	1.152794000	-1.223269000	0.368680000
1	3.108824000	-2.732323000	-0.051743000
1	5.224348000	-2.396392000	1.224890000
1	5.389140000	-0.572384000	2.898994000
6	-2.319948000	6.725898000	-7.299809000
6	-2.584869000	6.236299000	-5.860001000
6	-3.941847000	5.538689000	-5.664081000
6	-2.181587000	5.592927000	-8.330956000
1	-3.135178000	7.399847000	-7.603279000

1	-1.774542000	5.552396000	-5.561341000
1	-2.524525000	7.097872000	-5.178419000
1	-4.765755000	6.204692000	-5.953428000
1	-4.020477000	4.631097000	-6.274076000
1	-4.087625000	5.251673000	-4.616858000
1	-1.957256000	5.990577000	-9.327913000
1	-3.101376000	5.002565000	-8.409325000
1	-1.369235000	4.908560000	-8.053370000
6	-2.350657000	6.358917000	-1.474507000
6	-1.082858000	5.796717000	-0.775950000
6	-0.719619000	4.358305000	-1.187637000
6	0.335050000	3.701753000	-0.271364000
7	-0.200233000	3.473465000	1.086545000
6	-0.101505000	4.322652000	2.120337000
7	0.918233000	5.212360000	2.214399000
7	-1.043638000	4.277073000	3.083139000
6	-3.592001000	5.512116000	-1.227107000
8	-3.985471000	4.677758000	-2.095690000
1	-2.199877000	6.375181000	-2.557921000
1	-1.233292000	5.831755000	0.308617000
1	-0.241043000	6.468950000	-0.991846000
1	-1.614628000	3.726962000	-1.184646000
1	-0.338291000	4.347312000	-2.217834000
1	0.606436000	2.720797000	-0.672269000
1	1.247574000	4.300934000	-0.221323000
1	-0.852348000	2.659954000	1.203715000
1	1.808059000	5.078085000	1.718930000
1	0.849831000	5.976467000	2.866763000
1	-1.914956000	3.705172000	2.930881000
1	-0.860324000	4.674712000	3.991146000
7	-4.241093000	5.639069000	-0.039148000
6	-5.397572000	4.763402000	0.290286000
6	-5.983727000	5.397549000	1.563007000
6	-4.749937000	6.027870000	2.244053000
6	-3.931848000	6.583725000	1.061744000
1	-5.035875000	3.746170000	0.476548000
1	-6.718059000	6.171154000	1.304633000
1	-6.478895000	4.657994000	2.197821000
1	-5.006372000	6.804810000	2.970653000
1	-4.178724000	5.243239000	2.751779000
1	-2.862185000	6.607960000	1.280464000
1	-4.253173000	7.601052000	0.795207000
6	-0.320138000	-3.250729000	-7.032676000
6	1.102768000	-2.757703000	-7.170198000
6	1.377799000	-1.464032000	-7.648283000
6	2.692733000	-0.991095000	-7.735673000
6	3.762304000	-1.807737000	-7.348159000
6	3.504128000	-3.101628000	-6.878423000
6	2.187653000	-3.569838000	-6.793048000
1	-0.384992000	-4.334081000	-7.181015000

1	-0.719518000	-3.035846000	-6.031877000
1	0.553344000	-0.825008000	-7.952163000
1	2.882059000	0.009404000	-8.114030000
1	4.782929000	-1.444709000	-7.420332000
1	4.326974000	-3.748468000	-6.587682000
1	1.994679000	-4.575170000	-6.428860000
6	-4.710182000	-1.741345000	-0.096754000
6	-3.802738000	-2.783099000	-0.007333000
6	-4.013439000	-3.990570000	0.760827000
6	-2.877906000	-4.740063000	0.653380000
6	-1.960915000	-4.008436000	-0.193743000
7	-2.545683000	-2.818033000	-0.590723000
6	-0.707347000	-4.461511000	-0.570153000
6	0.133457000	-3.803679000	-1.453819000
6	1.381237000	-4.340203000	-1.952974000
6	1.863440000	-3.450331000	-2.872598000
6	0.924678000	-2.351639000	-2.935303000
7	-0.119916000	-2.580431000	-2.052310000
6	1.067511000	-1.246631000	-3.757319000
6	0.187978000	-0.174513000	-3.797882000
6	0.387049000	1.017728000	-4.592573000
6	-0.651172000	1.865128000	-4.315744000
6	-1.507264000	1.194589000	-3.362497000
7	-0.980808000	-0.054233000	-3.065517000
6	-2.686934000	1.718455000	-2.854103000
6	-3.542379000	1.037465000	-2.002826000
6	-4.833366000	1.536058000	-1.583008000
6	-5.418006000	0.551921000	-0.835817000
6	-4.486541000	-0.553586000	-0.774434000
7	-3.342355000	-0.234157000	-1.487461000
1	-5.656742000	-1.854474000	0.419626000
1	-0.381045000	-5.419958000	-0.181926000
1	1.931325000	-1.213454000	-4.411224000
1	-2.979356000	2.720768000	-3.146085000
8	-3.158625000	2.688083000	2.673229000
6	-2.801792000	1.484640000	2.342386000
8	-1.787172000	1.260468000	1.553394000
6	-3.596137000	0.377228000	2.934584000
6	-5.028148000	0.730416000	3.278566000
6	-5.207586000	1.253925000	4.725520000
1	-1.403263000	0.011045000	0.285059000
1	-5.364932000	1.511675000	2.591024000
1	-5.679896000	-0.138639000	3.120891000
1	-4.611599000	2.161282000	4.854941000
1	-4.880169000	0.512482000	5.463006000
8	-0.913233000	-0.581107000	-0.355340000
1	-0.982776000	-2.768703000	-7.759481000
1	-1.401481000	7.330783000	-7.307837000
1	-6.094274000	4.735855000	-0.551307000
1	-2.514498000	7.392860000	-1.145237000

1	6.331707000	4.094304000	0.348701000
1	3.417203000	3.468754000	3.639061000
1	-6.388697000	0.555935000	-0.361445000
1	-4.911494000	-4.209506000	1.318955000
1	-0.845808000	2.846926000	-4.723029000
1	-5.197283000	2.518519000	-1.841463000
1	-2.662084000	-5.699518000	1.100109000
1	1.805160000	-5.286080000	-1.647469000
1	1.214995000	1.163569000	-5.271017000
1	2.759500000	-3.518237000	-3.471761000
6	-3.023156000	-0.884687000	3.336706000
6	-3.808177000	-1.853870000	4.035508000
6	-1.649071000	-1.212565000	3.123285000
6	-3.257934000	-3.049676000	4.488319000
1	-4.855481000	-1.658177000	4.230421000
6	-1.110341000	-2.412617000	3.577309000
1	-1.028066000	-0.511806000	2.585184000
6	-1.904270000	-3.341799000	4.265846000
1	-3.885753000	-3.759274000	5.019701000
1	-0.062470000	-2.624941000	3.388304000
1	-1.478190000	-4.275335000	4.620082000
1	-6.260031000	1.493106000	4.923421000

$^{4}Pr_{OH,alpha,S2}$

26	-2.917056000	-1.678054000	-1.845623000
16	-3.458550000	-2.520612000	-4.136865000
1	-4.228826000	-1.444554000	-4.536453000
6	5.268482000	4.516208000	-0.979369000
6	5.684505000	3.233775000	-1.726853000
6	4.535959000	2.450928000	-2.413142000
6	3.911838000	3.252528000	-3.572398000
6	5.050068000	1.084104000	-2.907553000
6	4.356754000	4.360691000	0.230848000
8	3.604920000	5.319714000	0.580319000
1	4.761669000	5.214516000	-1.651664000
1	6.419672000	3.513624000	-2.494830000
1	6.226718000	2.560321000	-1.044864000
1	3.749113000	2.261299000	-1.666646000
1	3.098018000	2.686239000	-4.039619000
1	3.496182000	4.210049000	-3.238514000
1	4.661842000	3.460911000	-4.347103000
1	4.254023000	0.517051000	-3.402846000
1	5.421711000	0.475354000	-2.074578000
1	5.869284000	1.213202000	-3.627950000
7	4.425523000	3.210161000	0.940357000
6	3.629427000	2.970620000	2.153031000
6	3.996816000	1.623309000	2.798831000
6	3.684729000	0.421938000	1.921399000
6	2.354236000	0.113617000	1.581049000

6	2.060715000	-0.996484000	0.784658000
6	3.095158000	-1.814411000	0.310085000
6	4.422301000	-1.514719000	0.634780000
6	4.714160000	-0.402678000	1.437062000
1	4.963893000	2.433803000	0.581543000
1	2.565636000	2.977563000	1.893536000
1	5.061341000	1.626043000	3.067373000
1	3.437636000	1.551278000	3.740857000
1	1.535420000	0.733812000	1.933682000
1	1.026736000	-1.223374000	0.548353000
1	2.860803000	-2.680034000	-0.300422000
1	5.228492000	-2.148254000	0.277039000
1	5.745574000	-0.189617000	1.709772000
6	-1.335705000	6.539702000	-6.767357000
6	-1.850345000	6.125668000	-5.372142000
6	-3.295106000	5.597659000	-5.357420000
6	-1.210873000	5.372718000	-7.761674000
1	-2.009183000	7.304333000	-7.182877000
1	-1.176288000	5.359595000	-4.956442000
1	-1.779114000	6.993923000	-4.699698000
1	-3.984971000	6.344420000	-5.772791000
1	-3.395760000	4.685243000	-5.956591000
1	-3.620748000	5.364896000	-4.337321000
1	-0.805234000	5.713322000	-8.721762000
1	-2.181322000	4.904238000	-7.959895000
1	-0.540301000	4.595731000	-7.371938000
6	-2.847724000	6.633892000	-0.987357000
6	-1.435512000	6.251778000	-0.470753000
6	-0.810096000	5.032632000	-1.170686000
6	0.487695000	4.551105000	-0.488553000
7	0.211573000	4.007815000	0.854179000
6	0.394330000	4.637429000	2.020648000
7	1.287967000	5.653593000	2.138800000
7	-0.315471000	4.233258000	3.095634000
6	-3.864498000	5.505627000	-0.871279000
8	-4.141551000	4.782719000	-1.873473000
1	-2.793110000	6.887789000	-2.050020000
1	-1.485215000	6.054025000	0.606807000
1	-0.777139000	7.122888000	-0.594534000
1	-1.522669000	4.199654000	-1.191440000
1	-0.584933000	5.278677000	-2.217899000
1	0.933929000	3.744522000	-1.079350000
1	1.222917000	5.356339000	-0.425979000
1	-0.267176000	3.070143000	0.892057000
1	2.121255000	5.674330000	1.539621000
1	1.260779000	6.252689000	2.947784000
1	-1.065283000	3.506403000	3.005576000
1	-0.010275000	4.491189000	4.021326000
7	-4.441385000	5.271021000	0.337932000
6	-5.391515000	4.140643000	0.514384000

6	-5.871181000	4.279930000	1.971747000
6	-4.707370000	5.015460000	2.672746000
6	-4.228307000	6.014143000	1.602965000
1	-4.868554000	3.195121000	0.337264000
1	-6.786609000	4.883228000	2.018459000
1	-6.084596000	3.308545000	2.425178000
1	-5.009035000	5.512437000	3.599601000
1	-3.905392000	4.304106000	2.901949000
1	-3.180640000	6.291075000	1.732451000
1	-4.832429000	6.933379000	1.615775000
6	-0.230038000	-2.753584000	-6.791187000
6	1.197889000	-2.542353000	-6.343020000
6	1.820289000	-1.287468000	-6.469309000
6	3.131290000	-1.081891000	-6.021873000
6	3.849951000	-2.133119000	-5.438389000
6	3.245690000	-3.390911000	-5.312677000
6	1.935608000	-3.590979000	-5.762621000
1	-0.385090000	-3.771540000	-7.165198000
1	-0.937160000	-2.602666000	-5.963489000
1	1.270556000	-0.467916000	-6.923756000
1	3.595426000	-0.106764000	-6.140693000
1	4.869645000	-1.978249000	-5.099045000
1	3.798378000	-4.217925000	-4.875752000
1	1.474670000	-4.569790000	-5.663704000
6	-6.223355000	-1.722976000	-0.931775000
6	-5.467251000	-2.847971000	-0.645789000
6	-5.986634000	-4.056074000	-0.043574000
6	-4.940104000	-4.924563000	0.083480000
6	-3.770264000	-4.263439000	-0.451884000
7	-4.105109000	-2.986109000	-0.880753000
6	-2.520627000	-4.849677000	-0.567345000
6	-1.418659000	-4.250419000	-1.160215000
6	-0.141732000	-4.894958000	-1.357427000
6	0.664586000	-4.002466000	-2.009725000
6	-0.103119000	-2.793840000	-2.199030000
7	-1.382081000	-2.962796000	-1.679816000
6	0.391586000	-1.631719000	-2.770628000
6	-0.316775000	-0.445586000	-2.879146000
6	0.216206000	0.781469000	-3.423448000
6	-0.774559000	1.721398000	-3.345492000
6	-1.931918000	1.074141000	-2.770826000
7	-1.633223000	-0.250987000	-2.477290000
6	-3.169112000	1.677197000	-2.605479000
6	-4.309419000	1.016583000	-2.175445000
6	-5.610971000	1.634408000	-2.074047000
6	-6.479575000	0.669855000	-1.640075000
6	-5.715625000	-0.542080000	-1.452678000
7	-4.388661000	-0.320479000	-1.800890000
1	-7.282636000	-1.756356000	-0.701248000
1	-2.404337000	-5.863722000	-0.200646000

1	1.408084000	-1.648462000	-3.146377000
1	-3.267594000	2.731714000	-2.837286000
8	-2.129889000	2.186283000	2.849852000
6	-1.781484000	1.344499000	1.946517000
8	-0.887947000	1.576247000	1.037833000
6	-2.412100000	-0.071482000	1.901716000
6	-3.964501000	-0.034307000	1.922939000
6	-4.639355000	0.343161000	3.248577000
1	-1.358323000	-0.042819000	0.222059000
1	-4.280223000	0.653442000	1.128707000
1	-4.285531000	-1.034991000	1.611151000
1	-4.330066000	1.338398000	3.579125000
1	-4.391530000	-0.370019000	4.041660000
8	-2.051428000	-0.640862000	0.597862000
1	-0.506187000	-2.053874000	-7.587331000
1	-0.353585000	7.022337000	-6.656727000
1	-6.199840000	4.211677000	-0.218620000
1	-3.197383000	7.524783000	-0.450705000
1	6.170180000	5.039549000	-0.627926000
1	3.805643000	3.793700000	2.854846000
1	-7.538950000	0.759881000	-1.447065000
1	-7.018647000	-4.204421000	0.239427000
1	-0.752126000	2.751786000	-3.669967000
1	-5.794227000	2.674504000	-2.298433000
1	-4.945852000	-5.926407000	0.487772000
1	0.090017000	-5.902778000	-1.044501000
1	1.215049000	0.884804000	-3.821401000
1	1.682312000	-4.135946000	-2.346143000
6	-1.831547000	-0.964563000	3.011899000
6	-1.572795000	-2.317043000	2.731891000
6	-1.584578000	-0.481095000	4.309061000
6	-1.081011000	-3.167503000	3.727665000
1	-1.749774000	-2.683501000	1.728357000
6	-1.089741000	-1.334700000	5.302367000
1	-1.777330000	0.562187000	4.531161000
6	-0.836661000	-2.680971000	5.017115000
1	-0.884485000	-4.209660000	3.493121000
1	-0.901967000	-0.945428000	6.299142000
1	-0.452336000	-3.341587000	5.788677000
1	-5.729403000	0.335141000	3.122927000

$^{4}Pr_{DS,alpha,S2}$

26	-2.409549000	-1.700266000	-1.312657000
16	-2.276809000	-2.998277000	-3.480116000
1	-3.496260000	-2.623811000	-4.012568000
6	4.619721000	4.028420000	-1.826593000
6	5.380441000	3.146222000	-2.834438000
6	4.523372000	2.159206000	-3.670176000
6	3.569948000	2.893024000	-4.634410000

6	5.438840000	1.190750000	-4.445123000
6	3.992461000	3.366058000	-0.604562000
8	3.184136000	4.029243000	0.106828000
1	3.811309000	4.577425000	-2.321013000
1	5.906295000	3.812645000	-3.532827000
1	6.176487000	2.589580000	-2.315242000
1	3.906049000	1.560202000	-2.981520000
1	2.987531000	2.173076000	-5.220294000
1	2.863854000	3.543590000	-4.104482000
1	4.137143000	3.516379000	-5.338676000
1	4.848586000	0.476248000	-5.028411000
1	6.085973000	0.622024000	-3.765249000
1	6.084912000	1.743742000	-5.140418000
7	4.364240000	2.097954000	-0.300825000
6	3.876238000	1.341348000	0.857860000
6	5.037291000	0.654652000	1.615162000
6	5.827493000	-0.308984000	0.747896000
6	5.346274000	-1.607615000	0.497513000
6	6.048184000	-2.482139000	-0.338680000
6	7.244707000	-2.072616000	-0.941487000
6	7.737613000	-0.786509000	-0.696727000
6	7.033916000	0.087165000	0.143022000
1	4.998981000	1.613041000	-0.919905000
1	3.149255000	0.588365000	0.526292000
1	5.702268000	1.428299000	2.017320000
1	4.604882000	0.121323000	2.470931000
1	4.419419000	-1.933521000	0.961864000
1	5.665257000	-3.481975000	-0.517096000
1	7.788967000	-2.751990000	-1.589233000
1	8.670438000	-0.465459000	-1.149642000
1	7.432248000	1.077944000	0.346151000
6	-0.113183000	7.204907000	-5.667479000
6	-1.079521000	6.620531000	-4.614563000
6	-2.214913000	5.761993000	-5.197769000
6	0.699573000	6.144532000	-6.429891000
1	-0.688858000	7.808915000	-6.384690000
1	-0.501501000	6.021766000	-3.893141000
1	-1.517634000	7.451060000	-4.041411000
1	-2.810363000	6.339607000	-5.917318000
1	-1.824393000	4.883045000	-5.724806000
1	-2.886014000	5.408005000	-4.407022000
1	1.401822000	6.612319000	-7.130156000
1	0.050956000	5.477127000	-7.008053000
1	1.282102000	5.523458000	-5.736326000
6	-3.191486000	6.050735000	-0.744755000
6	-2.133369000	5.372389000	0.167023000
6	-1.444849000	4.167996000	-0.498523000
6	-0.138145000	3.739450000	0.186261000
7	-0.364053000	3.330064000	1.583263000
6	0.411502000	3.669166000	2.619781000

7	1.638119000	4.222755000	2.433153000
7	-0.038811000	3.463662000	3.872277000
6	-4.265160000	5.102182000	-1.257489000
8	-4.128096000	4.496968000	-2.359906000
1	-2.689430000	6.447504000	-1.632981000
1	-2.589496000	5.058574000	1.114663000
1	-1.381820000	6.133704000	0.421546000
1	-2.133764000	3.317209000	-0.543885000
1	-1.202085000	4.411386000	-1.539982000
1	0.309702000	2.910588000	-0.375235000
1	0.578460000	4.565874000	0.170529000
1	-1.187963000	2.713174000	1.776057000
1	2.148457000	4.161547000	1.547075000
1	2.095207000	4.679016000	3.205761000
1	-1.017882000	3.122025000	4.041818000
1	0.600250000	3.511106000	4.649599000
7	-5.375693000	4.914456000	-0.491404000
6	-6.473036000	4.028991000	-0.952772000
6	-7.628438000	4.350938000	0.012508000
6	-6.912092000	4.781650000	1.312801000
6	-5.678245000	5.557328000	0.810816000
1	-6.159015000	2.980379000	-0.885494000
1	-8.232094000	5.178860000	-0.379505000
1	-8.292664000	3.494832000	0.161240000
1	-7.542097000	5.389945000	1.968159000
1	-6.590761000	3.898992000	1.876927000
1	-4.832224000	5.478593000	1.498622000
1	-5.909883000	6.622855000	0.670236000
6	-0.429048000	-0.160715000	-6.935653000
6	0.893776000	-0.834531000	-6.641851000
6	2.054599000	-0.500328000	-7.359882000
6	3.278857000	-1.123324000	-7.082029000
6	3.359230000	-2.092165000	-6.074611000
6	2.207691000	-2.431942000	-5.351266000
6	0.985657000	-1.811795000	-5.631993000
1	-0.399641000	0.382858000	-7.886194000
1	-1.243220000	-0.892330000	-6.983422000
1	1.996032000	0.243076000	-8.151075000
1	4.161348000	-0.861374000	-7.659237000
1	4.304008000	-2.585162000	-5.864263000
1	2.255138000	-3.187683000	-4.572748000
1	0.095735000	-2.092141000	-5.073149000
6	-5.507825000	-3.042082000	-0.635722000
6	-4.402761000	-3.716904000	-0.139977000
6	-4.453675000	-4.972431000	0.578412000
6	-3.165991000	-5.318906000	0.877509000
6	-2.308418000	-4.285423000	0.337321000
7	-3.083031000	-3.301840000	-0.261779000
6	-0.922225000	-4.311812000	0.367777000
6	-0.092463000	-3.375893000	-0.236280000

6	1.351915000	-3.447322000	-0.252314000
6	1.802077000	-2.367269000	-0.962561000
6	0.637553000	-1.615036000	-1.375494000
7	-0.509803000	-2.251462000	-0.930370000
6	0.677426000	-0.421830000	-2.083632000
6	-0.432347000	0.332536000	-2.434064000
6	-0.382991000	1.591095000	-3.149939000
6	-1.671999000	2.026534000	-3.291471000
6	-2.525062000	1.027643000	-2.683153000
7	-1.749934000	0.006383000	-2.154377000
6	-3.911264000	1.073190000	-2.697602000
6	-4.738642000	0.058193000	-2.235218000
6	-6.182078000	0.074236000	-2.310511000
6	-6.629666000	-1.094161000	-1.755071000
6	-5.465927000	-1.832965000	-1.319991000
7	-4.316953000	-1.122379000	-1.638219000
1	-6.483203000	-3.484877000	-0.464239000
1	-0.449003000	-5.148330000	0.870816000
1	1.650724000	-0.053279000	-2.389587000
1	-4.372263000	1.967344000	-3.101764000
8	-2.481166000	2.419912000	4.245247000
6	-2.942104000	1.651551000	3.333604000
8	-2.476705000	1.631829000	2.111906000
6	-4.069458000	0.718809000	3.703802000
6	-5.090860000	0.447777000	2.856878000
6	-5.334651000	0.996492000	1.480716000
1	-2.491811000	0.231624000	1.162352000
1	-2.869477000	-1.331013000	1.375941000
1	-5.849091000	-0.252691000	3.207584000
1	-4.701120000	1.856599000	1.258847000
1	-6.391038000	1.274917000	1.368381000
8	-2.503783000	-0.683635000	0.748988000
1	-0.693933000	0.555061000	-6.146480000
1	0.580181000	7.897609000	-5.168604000
1	-6.701744000	4.240694000	-2.000858000
1	-3.641005000	6.897246000	-0.212220000
1	5.296345000	4.800826000	-1.433190000
1	3.355823000	2.041498000	1.513738000
1	-7.649396000	-1.431831000	-1.637697000
1	-5.361901000	-5.509847000	0.810184000
1	-2.038021000	2.932289000	-3.751006000
1	-6.762485000	0.875300000	-2.744956000
1	-2.813810000	-6.196822000	1.399625000
1	1.930174000	-4.234566000	0.210227000
1	0.525581000	2.063282000	-3.495705000
1	2.821762000	-2.096681000	-1.196440000
6	-4.036027000	0.130169000	5.074321000
6	-5.221656000	-0.078676000	5.805891000
6	-2.818662000	-0.269066000	5.662050000
6	-5.196340000	-0.685544000	7.065300000

1	-6.166684000	0.260410000	5.393985000
6	-2.791864000	-0.875323000	6.919957000
1	-1.889672000	-0.095427000	5.130541000
6	-3.981087000	-1.090996000	7.627254000
1	-6.123866000	-0.829496000	7.611448000
1	-1.842027000	-1.180019000	7.349248000
1	-3.959054000	-1.558753000	8.606693000
1	-5.121543000	0.238429000	0.717272000

$^{4}TS1_{beta,HA,S2}$

26	-3.935082000	-2.792815000	-2.087393000
16	-5.638236000	-4.178411000	-2.993230000
1	-6.191860000	-3.272876000	-3.877992000
6	5.540773000	4.866992000	0.105681000
6	5.475702000	3.323854000	-0.051173000
6	4.052526000	2.715536000	-0.003883000
6	3.216416000	3.083543000	-1.244972000
6	4.132329000	1.183271000	0.145463000
6	5.105305000	5.367272000	1.474019000
8	4.068332000	6.070560000	1.631897000
1	4.899813000	5.356188000	-0.630813000
1	5.944720000	3.058515000	-1.009499000
1	6.089860000	2.854225000	0.731589000
1	3.539563000	3.112872000	0.885001000
1	2.183080000	2.740042000	-1.126073000
1	3.188331000	4.165029000	-1.421899000
1	3.635814000	2.611378000	-2.143817000
1	3.129175000	0.744870000	0.185311000
1	4.661866000	0.896325000	1.061168000
1	4.659100000	0.738222000	-0.709907000
7	5.904646000	5.015625000	2.520025000
6	5.612629000	5.342671000	3.918443000
6	5.892014000	4.163546000	4.869869000
6	5.145136000	2.876266000	4.554348000
6	3.780796000	2.877329000	4.213012000
6	3.108193000	1.681441000	3.940439000
6	3.786600000	0.458326000	4.014352000
6	5.142263000	0.442721000	4.359303000
6	5.814415000	1.642857000	4.621380000
1	6.714068000	4.440405000	2.332377000
1	4.563717000	5.642887000	3.958262000
1	6.971306000	3.956741000	4.883065000
1	5.645409000	4.505418000	5.886503000
1	3.242090000	3.817295000	4.137834000
1	2.061642000	1.698705000	3.652947000
1	3.261014000	-0.463783000	3.790489000
1	5.679178000	-0.499142000	4.416278000
1	6.869368000	1.622390000	4.883509000
6	0.801568000	1.997806000	-4.446573000

6	-0.123903000	3.091021000	-3.871538000
6	-1.464392000	3.243498000	-4.610579000
6	0.241969000	0.571188000	-4.316026000
1	1.003391000	2.217877000	-5.507670000
1	-0.309496000	2.880403000	-2.808039000
1	0.406930000	4.054072000	-3.904920000
1	-1.300655000	3.453827000	-5.677400000
1	-2.070432000	2.331716000	-4.542342000
1	-2.042130000	4.071028000	-4.185558000
1	0.964457000	-0.173738000	-4.674113000
1	-0.681115000	0.442893000	-4.891291000
1	0.012030000	0.334784000	-3.269508000
6	-2.479548000	7.489027000	-0.916116000
6	-1.224053000	7.193978000	-0.055467000
6	-0.068611000	6.534018000	-0.829647000
6	1.097701000	6.125849000	0.088891000
7	0.679944000	5.053275000	1.009925000
6	0.757665000	5.101095000	2.341527000
7	1.595481000	5.980402000	2.961012000
7	-0.009178000	4.274337000	3.080316000
6	-3.032912000	6.240928000	-1.590838000
8	-2.711315000	5.962930000	-2.777887000
1	-2.217818000	8.180068000	-1.723120000
1	-1.501367000	6.545352000	0.785467000
1	-0.875718000	8.140541000	0.383115000
1	-0.429461000	5.651993000	-1.371796000
1	0.315096000	7.226456000	-1.591261000
1	1.947745000	5.779721000	-0.505520000
1	1.442800000	6.982176000	0.674604000
1	0.250353000	4.189110000	0.585197000
1	2.474979000	6.243316000	2.511638000
1	1.453562000	6.207094000	3.931679000
1	-0.715480000	3.641519000	2.623516000
1	0.219545000	4.116135000	4.049423000
7	-3.835962000	5.413318000	-0.863389000
6	-4.321430000	4.137323000	-1.446830000
6	-5.323427000	3.616662000	-0.399620000
6	-4.800907000	4.205507000	0.930638000
6	-4.308733000	5.608960000	0.527027000
1	-3.474004000	3.454990000	-1.586530000
1	-6.329667000	4.000164000	-0.613455000
1	-5.377644000	2.524376000	-0.385888000
1	-5.566613000	4.244598000	1.711452000
1	-3.957082000	3.613470000	1.304049000
1	-3.505471000	5.964733000	1.176376000
1	-5.126642000	6.344276000	0.557145000
6	1.074096000	-1.762215000	-7.997266000
6	-0.202216000	-1.130814000	-8.511220000
6	-1.158891000	-1.889256000	-9.207673000
6	-2.343216000	-1.303732000	-9.673314000

6	-2.589242000	0.055560000	-9.450747000
6	-1.643585000	0.823835000	-8.757352000
6	-0.462926000	0.234976000	-8.293315000
1	1.081059000	-1.811511000	-6.900327000
1	1.197591000	-2.781029000	-8.378975000
1	-0.972074000	-2.944076000	-9.390394000
1	-3.068208000	-1.907806000	-10.210164000
1	-3.503413000	0.512968000	-9.816014000
1	-1.823956000	1.879587000	-8.579326000
1	0.263096000	0.838225000	-7.755392000
6	-5.586210000	-3.456313000	0.863756000
6	-4.562988000	-4.309843000	0.486106000
6	-4.108948000	-5.437417000	1.270988000
6	-3.064985000	-6.003952000	0.597159000
6	-2.870554000	-5.233727000	-0.612094000
7	-3.796849000	-4.209043000	-0.668286000
6	-1.898792000	-5.493601000	-1.567775000
6	-1.729310000	-4.771677000	-2.735863000
6	-0.761399000	-5.088872000	-3.765246000
6	-0.946088000	-4.195875000	-4.780298000
6	-2.023767000	-3.313744000	-4.383674000
7	-2.483229000	-3.673899000	-3.124486000
6	-2.511864000	-2.277338000	-5.159572000
6	-3.529157000	-1.414031000	-4.777111000
6	-3.980601000	-0.282561000	-5.558816000
6	-4.942741000	0.351631000	-4.824645000
6	-5.098270000	-0.388749000	-3.590800000
7	-4.230071000	-1.474169000	-3.586381000
6	-5.992105000	-0.058416000	-2.584319000
6	-6.173278000	-0.776723000	-1.409894000
6	-7.178716000	-0.488307000	-0.407672000
6	-7.077636000	-1.457398000	0.551403000
6	-6.003833000	-2.342096000	0.150588000
7	-5.460772000	-1.904666000	-1.043944000
1	-6.097777000	-3.674239000	1.795189000
1	-1.238259000	-6.336488000	-1.396205000
1	-2.055658000	-2.113529000	-6.129867000
1	-6.618773000	0.813224000	-2.740152000
8	-1.588280000	2.452687000	1.869755000
6	-1.051412000	2.020571000	0.774088000
8	-0.272798000	2.713190000	0.023622000
6	-1.336350000	0.556875000	0.335748000
6	-2.841744000	0.292067000	0.294718000
6	-3.583589000	0.013522000	1.576831000
1	-0.953705000	0.493853000	-0.687361000
1	-3.359644000	0.968179000	-0.395014000
1	-2.931229000	-0.863348000	-0.504625000
1	-3.442938000	0.866265000	2.258640000
1	-3.195086000	-0.876414000	2.083815000
8	-2.774855000	-1.731217000	-1.291055000

1	1.956004000	-1.183340000	-8.296874000
1	1.772335000	2.048778000	-3.932405000
1	-4.763382000	4.318919000	-2.430258000
1	-3.241408000	7.973638000	-0.292988000
1	6.569920000	5.204776000	-0.081680000
1	6.206663000	6.210611000	4.235983000
1	-7.668807000	-1.578243000	1.447590000
1	-4.539856000	-5.737350000	2.215146000
1	-5.501279000	1.240829000	-5.078726000
1	-7.873203000	0.337997000	-0.455388000
1	-2.471138000	-6.861347000	0.878702000
1	-0.050623000	-5.900407000	-3.706064000
1	-3.590742000	-0.019224000	-6.531144000
1	-0.415882000	-4.125558000	-5.718868000
6	-0.558729000	-0.451529000	1.187876000
6	-0.044868000	-1.606642000	0.570126000
6	-0.361714000	-0.284290000	2.570488000
6	0.649502000	-2.567268000	1.313188000
1	-0.221635000	-1.762517000	-0.488791000
6	0.331241000	-1.248360000	3.314270000
1	-0.761329000	0.600313000	3.053227000
6	0.841773000	-2.392553000	2.689107000
1	1.033128000	-3.453797000	0.817239000
1	0.461966000	-1.109276000	4.384144000
1	1.372860000	-3.143000000	3.267593000
1	-4.653402000	-0.123784000	1.398606000

^{4}IM1$_{beta,S2}$

26	-3.954823000	-2.985146000	-2.257265000
16	-5.580339000	-4.369699000	-3.258910000
1	-5.944040000	-3.530866000	-4.294731000
6	5.532684000	4.740476000	-0.042568000
6	5.445732000	3.193609000	-0.118064000
6	4.020477000	2.608478000	0.038124000
6	3.126830000	2.912415000	-1.179812000
6	4.091383000	1.087412000	0.278277000
6	5.170730000	5.318984000	1.317010000
8	4.184453000	6.091670000	1.474612000
1	4.860319000	5.199449000	-0.770336000
1	5.858005000	2.874527000	-1.085988000
1	6.097635000	2.753326000	0.651353000
1	3.558266000	3.066552000	0.925784000
1	2.095499000	2.596286000	-0.989459000
1	3.104441000	3.981269000	-1.422592000
1	3.494266000	2.378640000	-2.066765000
1	3.086778000	0.664975000	0.389085000
1	4.658270000	0.851164000	1.186214000
1	4.574746000	0.583508000	-0.570106000
7	5.982386000	4.965045000	2.352556000

6	5.764018000	5.380906000	3.740570000
6	6.009369000	4.238770000	4.745461000
6	5.183129000	2.980721000	4.523867000
6	3.806015000	3.039250000	4.244131000
6	3.062191000	1.870088000	4.051530000
6	3.680264000	0.617037000	4.147740000
6	5.047624000	0.544507000	4.434339000
6	5.791089000	1.717435000	4.614549000
1	6.746987000	4.330979000	2.167120000
1	4.737826000	5.748514000	3.800072000
1	7.075371000	3.971681000	4.735810000
1	5.817210000	4.647319000	5.749133000
1	3.311687000	4.002105000	4.154371000
1	2.006147000	1.931227000	3.808014000
1	3.099581000	-0.284574000	3.984703000
1	5.538308000	-0.421098000	4.508370000
1	6.854661000	1.652002000	4.830494000
6	0.772140000	2.016954000	-4.406067000
6	-0.177209000	3.075281000	-3.805408000
6	-1.557547000	3.146007000	-4.480633000
6	0.306204000	0.566366000	-4.196268000
1	0.892482000	2.213365000	-5.484089000
1	-0.302018000	2.878553000	-2.730365000
1	0.301749000	4.062568000	-3.883708000
1	-1.454949000	3.333264000	-5.559343000
1	-2.116788000	2.210144000	-4.359408000
1	-2.150083000	3.959992000	-4.050002000
1	1.039003000	-0.148628000	-4.592584000
1	-0.650765000	0.371088000	-4.692221000
1	0.173730000	0.349415000	-3.128987000
6	-2.406575000	7.550263000	-0.970140000
6	-1.129857000	7.270668000	-0.135892000
6	-0.013000000	6.548877000	-0.911681000
6	1.170092000	6.154705000	-0.008018000
7	0.751153000	5.142953000	0.978387000
6	0.861702000	5.259806000	2.303277000
7	1.737096000	6.147703000	2.855896000
7	0.090018000	4.495610000	3.102043000
6	-3.010217000	6.282494000	-1.559486000
8	-2.739566000	5.937806000	-2.741953000
1	-2.157175000	8.192097000	-1.820523000
1	-1.392395000	6.669196000	0.743865000
1	-0.748943000	8.229721000	0.244696000
1	-0.409154000	5.651209000	-1.400949000
1	0.363595000	7.196433000	-1.715158000
1	1.991184000	5.753471000	-0.608877000
1	1.556854000	7.029366000	0.522001000
1	0.282895000	4.270568000	0.610013000
1	2.612658000	6.361157000	2.374175000
1	1.624993000	6.428322000	3.816301000

1	-0.644295000	3.858107000	2.694446000
1	0.336469000	4.383883000	4.073330000
7	-3.801722000	5.510718000	-0.761768000
6	-4.327758000	4.214216000	-1.258678000
6	-5.298050000	3.765576000	-0.150173000
6	-4.720193000	4.416452000	1.127138000
6	-4.222215000	5.787959000	0.631672000
1	-3.496126000	3.511392000	-1.390461000
1	-6.307144000	4.149546000	-0.349035000
1	-5.361704000	2.676363000	-0.074991000
1	-5.457149000	4.510360000	1.930755000
1	-3.872131000	3.831239000	1.501323000
1	-3.391440000	6.164302000	1.232935000
1	-5.027482000	6.537611000	0.649934000
6	1.126102000	-1.675804000	-7.985342000
6	-0.152797000	-1.054519000	-8.505220000
6	-1.079120000	-1.808987000	-9.245690000
6	-2.264886000	-1.232113000	-9.718678000
6	-2.542603000	0.114520000	-9.459633000
6	-1.627509000	0.878681000	-8.721929000
6	-0.445804000	0.298235000	-8.250074000
1	1.121566000	-1.741154000	-6.889341000
1	1.268712000	-2.686799000	-8.381034000
1	-0.867124000	-2.853531000	-9.457721000
1	-2.965932000	-1.832845000	-10.289985000
1	-3.457482000	0.565610000	-9.830934000
1	-1.832426000	1.924781000	-8.515623000
1	0.255758000	0.897700000	-7.676667000
6	-5.525087000	-3.752282000	0.711479000
6	-4.532512000	-4.609333000	0.264451000
6	-4.079084000	-5.781135000	0.980138000
6	-3.066551000	-6.334081000	0.248802000
6	-2.888839000	-5.510353000	-0.926259000
7	-3.800601000	-4.468250000	-0.905119000
6	-1.935951000	-5.734427000	-1.907023000
6	-1.761806000	-4.947855000	-3.032557000
6	-0.794026000	-5.212189000	-4.075747000
6	-0.961826000	-4.252986000	-5.032763000
6	-2.031688000	-3.386879000	-4.585958000
7	-2.498751000	-3.821070000	-3.356743000
6	-2.515281000	-2.305048000	-5.302117000
6	-3.542369000	-1.477181000	-4.877786000
6	-4.002957000	-0.312605000	-5.601268000
6	-4.978307000	0.271525000	-4.843404000
6	-5.130973000	-0.529224000	-3.648846000
7	-4.255771000	-1.606448000	-3.695738000
6	-6.010728000	-0.241032000	-2.617950000
6	-6.150997000	-0.994067000	-1.462349000
6	-7.126585000	-0.738589000	-0.426100000
6	-6.995517000	-1.731033000	0.505343000

6	-5.935191000	-2.603865000	0.054203000
7	-5.417412000	-2.126312000	-1.141140000
1	-6.016536000	-3.998756000	1.646064000
1	-1.286760000	-6.594856000	-1.788816000
1	-2.053953000	-2.081723000	-6.257619000
1	-6.646304000	0.630318000	-2.730639000
8	-1.571631000	2.668722000	2.027392000
6	-1.090826000	2.173548000	0.931709000
8	-0.308310000	2.804603000	0.131199000
6	-1.462347000	0.710259000	0.560148000
6	-2.961669000	0.508106000	0.580386000
6	-3.733425000	0.286830000	1.841546000
1	-1.115229000	0.583925000	-0.469864000
1	-3.498726000	0.876373000	-0.294563000
1	-3.039034000	-1.375839000	-0.693652000
1	-3.687482000	1.184779000	2.480541000
1	-3.313611000	-0.534508000	2.436267000
8	-2.723011000	-1.962889000	-1.423572000
1	2.003092000	-1.080344000	-8.266469000
1	1.769536000	2.139377000	-3.959098000
1	-4.802942000	4.351469000	-2.233832000
1	-3.136257000	8.084491000	-0.349008000
1	6.553940000	5.058219000	-0.297230000
1	6.420962000	6.225704000	3.989700000
1	-7.562974000	-1.877772000	1.412788000
1	-4.487382000	-6.116834000	1.922202000
1	-5.543166000	1.168000000	-5.053623000
1	-7.824615000	0.085907000	-0.433397000
1	-2.480945000	-7.214021000	0.471756000
1	-0.092546000	-6.033662000	-4.063985000
1	-3.606727000	0.005844000	-6.554228000
1	-0.425424000	-4.128695000	-5.962025000
6	-0.703530000	-0.298656000	1.433210000
6	-0.236211000	-1.487105000	0.842328000
6	-0.489165000	-0.099832000	2.808844000
6	0.436213000	-2.448272000	1.605370000
1	-0.429539000	-1.669243000	-0.210591000
6	0.180326000	-1.065316000	3.572325000
1	-0.855787000	0.811235000	3.268412000
6	0.648862000	-2.241541000	2.973853000
1	0.786488000	-3.359921000	1.130585000
1	0.325510000	-0.902097000	4.637005000
1	1.162773000	-2.991987000	3.567702000
1	-4.784831000	0.062384000	1.634829000

$^{4}TS2_{beta,DS,S2}$

26	-4.758231000	-0.661458000	-2.481316000
16	-6.660274000	-1.314461000	-3.943051000
1	-5.976749000	-1.363378000	-5.143370000

6	4.228998000	7.697211000	1.774233000
6	4.860723000	7.459760000	0.382676000
6	3.985577000	6.669580000	-0.619821000
6	2.674122000	7.408149000	-0.952106000
6	4.786938000	6.377345000	-1.903676000
6	3.873100000	6.441408000	2.558543000
8	2.725845000	6.301873000	3.070613000
1	3.306017000	8.274526000	1.686599000
1	5.099112000	8.441685000	-0.051222000
1	5.826586000	6.943085000	0.491674000
1	3.730277000	5.702165000	-0.160816000
1	2.085743000	6.836941000	-1.679437000
1	2.048811000	7.557418000	-0.064938000
1	2.882855000	8.392984000	-1.391583000
1	4.186661000	5.798929000	-2.614693000
1	5.691520000	5.798361000	-1.683459000
1	5.089162000	7.310624000	-2.397727000
7	4.855019000	5.515825000	2.708696000
6	4.698670000	4.260693000	3.454462000
6	5.524171000	3.115684000	2.835345000
6	5.093888000	2.713067000	1.435417000
6	3.817881000	2.165996000	1.208372000
6	3.413551000	1.779920000	-0.074222000
6	4.295447000	1.931850000	-1.153850000
6	5.568670000	2.473271000	-0.944162000
6	5.963196000	2.863242000	0.342842000
1	5.742524000	5.677744000	2.253754000
1	3.635969000	4.009689000	3.457247000
1	6.587362000	3.395681000	2.831266000
1	5.441378000	2.256622000	3.515529000
1	3.128953000	2.027753000	2.036983000
1	2.420534000	1.363608000	-0.216086000
1	3.990157000	1.618364000	-2.146831000
1	6.257636000	2.585763000	-1.776143000
1	6.960081000	3.268654000	0.501674000
6	1.899497000	3.615538000	-4.114095000
6	0.815611000	3.397381000	-3.036987000
6	-0.625825000	3.436747000	-3.569475000
6	1.979359000	2.493204000	-5.163477000
1	1.719393000	4.577195000	-4.619619000
1	0.986256000	2.436918000	-2.531402000
1	0.933133000	4.171676000	-2.264579000
1	-0.838247000	4.396908000	-4.060957000
1	-0.805237000	2.639292000	-4.299199000
1	-1.349291000	3.297182000	-2.758620000
1	2.791940000	2.672393000	-5.878746000
1	1.048329000	2.408287000	-5.735775000
1	2.160784000	1.525962000	-4.680321000
6	-3.319587000	4.810143000	0.333588000
6	-1.993004000	5.240986000	1.017739000

6	-0.736940000	4.811881000	0.231712000
6	0.520445000	4.702241000	1.116398000
7	0.433550000	3.491816000	1.949222000
6	0.391779000	3.428619000	3.281555000
7	0.817858000	4.459521000	4.060217000
7	-0.081787000	2.303214000	3.858028000
6	-3.514972000	3.302561000	0.411383000
8	-3.079719000	2.558793000	-0.518795000
1	-3.295944000	5.075061000	-0.727505000
1	-1.957169000	4.805142000	2.025754000
1	-1.994004000	6.331773000	1.149204000
1	-0.908509000	3.839364000	-0.244282000
1	-0.545129000	5.530116000	-0.576776000
1	1.414617000	4.621651000	0.488204000
1	0.643537000	5.586258000	1.747361000
1	0.323600000	2.581082000	1.418707000
1	1.464751000	5.170008000	3.707790000
1	0.567282000	4.483685000	5.034999000
1	-0.525034000	1.552334000	3.266121000
1	0.114754000	2.113470000	4.827999000
7	-4.100075000	2.792160000	1.523360000
6	-4.233039000	1.320456000	1.732493000
6	-5.187566000	1.216871000	2.932573000
6	-4.898236000	2.500763000	3.741125000
6	-4.664751000	3.568182000	2.653804000
1	-3.246929000	0.897860000	1.954811000
1	-6.230512000	1.205628000	2.594086000
1	-5.015557000	0.305779000	3.512441000
1	-5.709059000	2.777567000	4.422085000
1	-3.984663000	2.371666000	4.334265000
1	-3.978507000	4.352821000	2.984832000
1	-5.610196000	4.045257000	2.358060000
6	1.076011000	-3.894629000	-6.334910000
6	0.544891000	-5.105309000	-5.596874000
6	0.677894000	-5.208001000	-4.199873000
6	0.217037000	-6.340348000	-3.518634000
6	-0.390499000	-7.388866000	-4.221771000
6	-0.535583000	-7.294309000	-5.610982000
6	-0.071888000	-6.161671000	-6.289927000
1	0.954070000	-2.982412000	-5.740664000
1	2.148196000	-3.999440000	-6.551162000
1	1.138264000	-4.394645000	-3.646019000
1	0.329640000	-6.401206000	-2.440363000
1	-0.747304000	-8.267741000	-3.693599000
1	-1.011050000	-8.098256000	-6.164716000
1	-0.189614000	-6.094417000	-7.368217000
6	-7.007786000	1.465865000	-0.972435000
6	-6.962247000	0.154251000	-0.521657000
6	-7.782143000	-0.381107000	0.543458000
6	-7.411937000	-1.685519000	0.720853000

6	-6.368472000	-1.968264000	-0.239814000
7	-6.109534000	-0.832173000	-0.990023000
6	-5.746530000	-3.199002000	-0.386288000
6	-4.789591000	-3.498172000	-1.344111000
6	-4.212928000	-4.812474000	-1.548185000
6	-3.370608000	-4.720976000	-2.620466000
6	-3.421594000	-3.349638000	-3.083136000
7	-4.279540000	-2.617552000	-2.280475000
6	-2.741011000	-2.869997000	-4.191466000
6	-2.820437000	-1.569920000	-4.669246000
6	-2.086227000	-1.067004000	-5.809534000
6	-2.393716000	0.260070000	-5.935618000
6	-3.325234000	0.584334000	-4.877397000
7	-3.583313000	-0.548998000	-4.123602000
6	-3.865662000	1.844065000	-4.660145000
6	-4.769714000	2.160461000	-3.659084000
6	-5.397441000	3.454756000	-3.500067000
6	-6.289107000	3.346027000	-2.471141000
6	-6.214334000	1.985170000	-1.983211000
7	-5.266239000	1.283627000	-2.708511000
1	-7.725719000	2.131728000	-0.505876000
1	-6.058840000	-3.998697000	0.276735000
1	-2.098176000	-3.563526000	-4.721975000
1	-3.569865000	2.638071000	-5.336763000
8	-1.111127000	0.384218000	2.238678000
6	-0.569390000	0.310799000	1.069058000
8	0.219198000	1.201408000	0.582440000
6	-0.911103000	-0.919694000	0.188301000
6	-1.246141000	-2.090559000	1.011398000
6	-1.341681000	-3.485248000	0.508172000
1	-1.894515000	-0.619776000	-0.355760000
1	-1.591264000	-1.871754000	2.018716000
1	-3.278796000	0.795925000	-1.117313000
1	-0.721450000	-3.656338000	-0.376092000
1	-2.380747000	-3.721732000	0.217305000
8	-3.311980000	-0.177271000	-1.322212000
1	0.564845000	-3.752934000	-7.293551000
1	2.876881000	3.705430000	-3.616635000
1	-4.613177000	0.845318000	0.827812000
1	-4.162914000	5.340598000	0.789765000
1	4.920765000	8.295414000	2.385313000
1	5.003085000	4.410389000	4.500130000
1	-6.955065000	4.100983000	-2.078396000
1	-8.540700000	0.177855000	1.072418000
1	-2.033828000	0.964891000	-6.671204000
1	-5.184490000	4.316811000	-4.115491000
1	-7.806267000	-2.404036000	1.424856000
1	-4.446411000	-5.682681000	-0.951561000
1	-1.423902000	-1.663005000	-6.420380000
1	-2.766276000	-5.494512000	-3.071626000

6	0.130896000	-1.153395000	-0.900043000
6	-0.119909000	-0.791202000	-2.231273000
6	1.381415000	-1.712413000	-0.576170000
6	0.860020000	-0.975391000	-3.215386000
1	-1.086039000	-0.369631000	-2.485271000
6	2.360854000	-1.899009000	-1.556230000
1	1.587399000	-1.989598000	0.454148000
6	2.103947000	-1.527440000	-2.883536000
1	0.645707000	-0.686512000	-4.239637000
1	3.323200000	-2.323764000	-1.285483000
1	2.865035000	-1.665329000	-3.646495000
1	-1.067065000	-4.211815000	1.285030000

$^{4}Pr_{beta,DS,S2}$

26	-5.094606000	-0.774972000	-3.081212000
16	-6.975605000	-1.664105000	-4.495999000
1	-6.367660000	-1.535852000	-5.731624000
6	4.769044000	7.862440000	0.950851000
6	4.903016000	7.343288000	-0.503939000
6	3.636152000	6.680230000	-1.094073000
6	2.427753000	7.636615000	-1.111640000
6	3.934714000	6.144201000	-2.507989000
6	4.386604000	6.787565000	1.956255000
8	3.242752000	6.770861000	2.492649000
1	4.003088000	8.638205000	1.011835000
1	5.189991000	8.194199000	-1.138841000
1	5.732765000	6.623570000	-0.560015000
1	3.380161000	5.818108000	-0.459746000
1	1.562854000	7.152201000	-1.580079000
1	2.132366000	7.938120000	-0.101162000
1	2.654104000	8.542578000	-1.690289000
1	3.058101000	5.636312000	-2.926054000
1	4.761126000	5.424036000	-2.492472000
1	4.205769000	6.962393000	-3.188711000
7	5.343205000	5.861979000	2.232831000
6	5.176054000	4.733992000	3.157343000
6	5.844054000	3.444790000	2.631577000
6	5.196112000	2.867936000	1.386813000
6	3.961628000	2.199775000	1.473467000
6	3.344160000	1.671923000	0.333876000
6	3.964736000	1.804108000	-0.916011000
6	5.196956000	2.460577000	-1.017020000
6	5.807285000	2.988898000	0.128023000
1	6.233045000	5.942353000	1.759951000
1	4.104187000	4.578965000	3.293789000
1	6.909645000	3.641644000	2.444716000
1	5.808622000	2.709733000	3.446485000
1	3.471490000	2.078675000	2.435298000
1	2.389535000	1.166666000	0.441850000

1	3.493584000	1.390005000	-1.801691000
1	5.687406000	2.553920000	-1.981556000
1	6.773103000	3.482385000	0.044803000
6	1.574205000	2.587366000	-4.554172000
6	0.684998000	2.814731000	-3.312532000
6	-0.823619000	2.831359000	-3.609813000
6	1.441486000	1.182926000	-5.168845000
1	1.329047000	3.344278000	-5.314746000
1	0.903211000	2.035040000	-2.567225000
1	0.970117000	3.769044000	-2.844249000
1	-1.073219000	3.638899000	-4.311759000
1	-1.160630000	1.889877000	-4.057586000
1	-1.408164000	2.976836000	-2.695266000
1	2.109023000	1.065283000	-6.031583000
1	0.418777000	0.983289000	-5.507735000
1	1.704037000	0.410590000	-4.433513000
6	-3.004351000	4.469396000	0.530941000
6	-1.694068000	4.880636000	1.253007000
6	-0.408638000	4.455207000	0.521717000
6	0.858112000	4.702060000	1.365271000
7	0.863729000	3.810850000	2.535128000
6	0.954233000	4.172492000	3.817849000
7	1.447854000	5.386125000	4.180530000
7	0.545011000	3.300246000	4.762119000
6	-3.201331000	2.964452000	0.440842000
8	-2.804771000	2.341033000	-0.597291000
1	-2.987691000	4.839699000	-0.498419000
1	-1.683468000	4.453954000	2.264186000
1	-1.699662000	5.973320000	1.374116000
1	-0.457931000	3.392544000	0.257489000
1	-0.315568000	5.008623000	-0.422772000
1	1.750922000	4.501855000	0.762687000
1	0.910598000	5.744013000	1.693025000
1	0.723024000	2.779602000	2.335441000
1	2.054485000	5.926594000	3.557769000
1	1.293722000	5.728813000	5.114612000
1	0.043980000	2.415088000	4.483546000
1	0.829793000	3.424784000	5.720683000
7	-3.770056000	2.315875000	1.479749000
6	-3.971082000	0.837839000	1.461179000
6	-4.722983000	0.554397000	2.773144000
6	-4.265794000	1.694714000	3.709040000
6	-4.201547000	2.912963000	2.770041000
1	-2.995299000	0.342143000	1.444983000
1	-5.806334000	0.595664000	2.607334000
1	-4.481390000	-0.435407000	3.169461000
1	-4.942997000	1.859002000	4.552546000
1	-3.262184000	1.479774000	4.094718000
1	-3.489961000	3.665988000	3.113298000
1	-5.186729000	3.387682000	2.657921000

6	1.923952000	-2.671694000	-7.015040000
6	1.263289000	-3.893274000	-6.413795000
6	1.708001000	-4.418017000	-5.186033000
6	1.091563000	-5.536849000	-4.616420000
6	0.015581000	-6.155951000	-5.266671000
6	-0.435581000	-5.646575000	-6.489714000
6	0.185722000	-4.525958000	-7.056819000
1	1.624749000	-1.755654000	-6.488221000
1	3.016560000	-2.736257000	-6.953594000
1	2.547042000	-3.948904000	-4.678732000
1	1.453152000	-5.929267000	-3.670805000
1	-0.457422000	-7.030339000	-4.830592000
1	-1.263680000	-6.122515000	-7.005827000
1	-0.163295000	-4.144155000	-8.012544000
6	-7.311543000	1.327698000	-1.501624000
6	-7.165477000	0.046062000	-0.986981000
6	-7.890792000	-0.473201000	0.151580000
6	-7.444959000	-1.749414000	0.365792000
6	-6.442598000	-2.025807000	-0.639104000
7	-6.290128000	-0.919294000	-1.460649000
6	-5.734002000	-3.215913000	-0.741049000
6	-4.762198000	-3.488397000	-1.692157000
6	-4.053615000	-4.745907000	-1.811863000
6	-3.219042000	-4.637617000	-2.888007000
6	-3.407611000	-3.313284000	-3.441798000
7	-4.342283000	-2.619138000	-2.687439000
6	-2.762648000	-2.831656000	-4.570973000
6	-2.962974000	-1.574219000	-5.126064000
6	-2.260336000	-1.068226000	-6.284055000
6	-2.700948000	0.210240000	-6.496737000
6	-3.677544000	0.500884000	-5.470047000
7	-3.835513000	-0.606240000	-4.650598000
6	-4.331423000	1.717468000	-5.322906000
6	-5.245197000	2.016852000	-4.324021000
6	-5.946516000	3.277908000	-4.200017000
6	-6.785221000	3.167912000	-3.127531000
6	-6.606866000	1.838375000	-2.581192000
7	-5.651876000	1.155347000	-3.317368000
1	-8.040084000	1.976631000	-1.027115000
1	-5.966822000	-3.997689000	-0.025768000
1	-2.048739000	-3.486441000	-5.058777000
1	-4.108918000	2.494060000	-6.046887000
8	-0.656723000	1.018127000	3.933693000
6	-0.200732000	0.541033000	2.821811000
8	0.555949000	1.213078000	2.016813000
6	-0.568611000	-0.874690000	2.431175000
6	-1.251304000	-1.612936000	3.337392000
6	-1.791960000	-3.005271000	3.185522000
1	-3.209792000	0.859657000	-1.376183000
1	-1.453673000	-1.117728000	4.285123000

1	-2.730252000	-0.691940000	-1.488593000
1	-1.759315000	-3.361465000	2.152824000
1	-2.832353000	-3.047619000	3.535439000
8	-3.406114000	-0.041847000	-1.746612000
1	1.653199000	-2.549374000	-8.068773000
1	2.625162000	2.758175000	-4.277338000
1	-4.520206000	0.538727000	0.566593000
1	-3.858073000	4.934418000	1.037836000
1	5.721174000	8.318476000	1.255599000
1	5.601921000	4.994459000	4.136742000
1	-7.473742000	3.903712000	-2.737363000
1	-8.643695000	0.073626000	0.701091000
1	-2.399991000	0.902378000	-7.270051000
1	-5.810752000	4.122042000	-4.860709000
1	-7.759734000	-2.449195000	1.126622000
1	-4.193816000	-5.592710000	-1.155503000
1	-1.527812000	-1.629430000	-6.845877000
1	-2.535230000	-5.372395000	-3.287238000
6	-0.162351000	-1.371067000	1.085565000
6	-0.435638000	-0.625748000	-0.081825000
6	0.485732000	-2.612410000	0.940303000
6	-0.103180000	-1.125186000	-1.346111000
1	-0.894823000	0.351660000	0.005897000
6	0.823144000	-3.109902000	-0.324424000
1	0.740807000	-3.183560000	1.827169000
6	0.522093000	-2.373882000	-1.473935000
1	-0.330810000	-0.538546000	-2.231517000
1	1.321595000	-4.071206000	-0.407765000
1	0.769294000	-2.763736000	-2.456518000
1	-1.231351000	-3.720437000	3.805763000

^{4}TS2$_{beta,DC,S2}$

26	-4.614826000	-2.801853000	-2.526036000
16	-6.316550000	-4.032097000	-3.623374000
1	-6.608813000	-3.103210000	-4.603050000
6	6.308032000	7.154142000	1.474304000
6	6.482002000	6.754232000	-0.014777000
6	5.172863000	6.584502000	-0.821282000
6	4.316933000	7.866085000	-0.827331000
6	5.502783000	6.141480000	-2.260528000
6	5.504000000	6.150941000	2.284282000
8	4.287958000	6.354547000	2.557345000
1	5.793184000	8.114059000	1.551988000
1	7.099455000	7.526285000	-0.496598000
1	7.054370000	5.817083000	-0.072427000
1	4.586377000	5.781857000	-0.350042000
1	3.429060000	7.730266000	-1.456040000
1	3.973870000	8.131148000	0.178032000
1	4.884496000	8.714004000	-1.234788000

1	4.586464000	5.975262000	-2.838032000
1	6.077190000	5.207618000	-2.268555000
1	6.092619000	6.908567000	-2.780137000
7	6.174229000	5.034911000	2.679958000
6	5.591459000	3.924597000	3.441891000
6	5.824720000	2.549847000	2.772844000
6	5.081066000	2.360290000	1.465080000
6	3.707736000	2.058346000	1.468281000
6	2.998031000	1.902955000	0.272066000
6	3.664659000	2.045363000	-0.952944000
6	5.034172000	2.335163000	-0.972122000
6	5.736308000	2.492666000	0.229731000
1	7.146898000	4.951769000	2.417070000
1	4.522768000	4.126362000	3.536025000
1	6.903448000	2.404874000	2.618765000
1	5.512889000	1.783566000	3.494956000
1	3.186764000	1.933256000	2.414154000
1	1.935970000	1.677967000	0.301305000
1	3.118594000	1.921565000	-1.882451000
1	5.556967000	2.430225000	-1.919179000
1	6.802814000	2.703784000	0.208199000
6	0.526273000	0.469293000	-3.408855000
6	-0.102436000	1.815349000	-2.987571000
6	-1.615971000	1.911788000	-3.243986000
6	0.118187000	-0.718653000	-2.520800000
1	0.249999000	0.258394000	-4.454195000
1	0.089741000	1.985819000	-1.918018000
1	0.399367000	2.629661000	-3.531650000
1	-1.844893000	1.747291000	-4.305626000
1	-2.169247000	1.158079000	-2.670887000
1	-1.988395000	2.903168000	-2.962924000
1	0.605870000	-1.645186000	-2.850754000
1	-0.962742000	-0.895256000	-2.533389000
1	0.415832000	-0.538917000	-1.479325000
6	-1.857234000	7.054505000	-0.446516000
6	-0.664605000	6.713452000	0.483475000
6	0.424049000	5.852670000	-0.181904000
6	1.512562000	5.402972000	0.811034000
7	0.941743000	4.499270000	1.825633000
6	0.990146000	4.659453000	3.151020000
7	1.881466000	5.501459000	3.732758000
7	0.128629000	3.969748000	3.929293000
6	-2.561043000	5.812034000	-0.978317000
8	-2.247646000	5.333569000	-2.102604000
1	-1.494618000	7.598841000	-1.323867000
1	-1.035841000	6.188510000	1.373030000
1	-0.220658000	7.654966000	0.838514000
1	-0.025783000	4.969763000	-0.651627000
1	0.906588000	6.418185000	-0.990848000
1	2.310734000	4.872693000	0.280201000

1	1.963880000	6.265843000	1.307256000
1	0.436313000	3.660516000	1.458951000
1	2.730759000	5.820394000	3.257521000
1	1.735750000	5.796448000	4.684606000
1	-0.656227000	3.422714000	3.515133000
1	0.312007000	3.867534000	4.914788000
7	-3.498250000	5.214505000	-0.189439000
6	-4.171680000	3.967904000	-0.634684000
6	-5.296116000	3.777023000	0.398548000
6	-4.719072000	4.419894000	1.679801000
6	-3.962985000	5.653670000	1.147535000
1	-3.454463000	3.138603000	-0.620297000
1	-6.200851000	4.311019000	0.079749000
1	-5.556697000	2.724155000	0.534654000
1	-5.488537000	4.691012000	2.409236000
1	-4.011016000	3.733851000	2.159095000
1	-3.124400000	5.929784000	1.791828000
1	-4.629449000	6.524525000	1.058465000
6	1.788359000	-2.876909000	-6.055886000
6	0.881686000	-2.610921000	-7.237686000
6	0.467148000	-3.658050000	-8.080512000
6	-0.401887000	-3.420543000	-9.152438000
6	-0.867471000	-2.124911000	-9.405905000
6	-0.455918000	-1.071136000	-8.579667000
6	0.409404000	-1.313833000	-7.506426000
1	1.203783000	-3.083677000	-5.149194000
1	2.435396000	-3.742671000	-6.234173000
1	0.834947000	-4.664791000	-7.898974000
1	-0.708954000	-4.243520000	-9.790662000
1	-1.536452000	-1.937771000	-10.239912000
1	-0.801020000	-0.059953000	-8.774472000
1	0.722139000	-0.491422000	-6.869212000
6	-6.732912000	-2.979452000	0.183527000
6	-5.824012000	-4.010009000	0.005965000
6	-5.668965000	-5.131109000	0.905687000
6	-4.633816000	-5.887743000	0.433950000
6	-4.149624000	-5.247910000	-0.768682000
7	-4.890687000	-4.104266000	-1.015948000
6	-3.115540000	-5.729518000	-1.556112000
6	-2.695521000	-5.154947000	-2.743740000
6	-1.682890000	-5.715140000	-3.613759000
6	-1.606618000	-4.909479000	-4.713546000
6	-2.561712000	-3.837975000	-4.523896000
7	-3.211055000	-4.002680000	-3.312877000
6	-2.767573000	-2.804818000	-5.422670000
6	-3.642968000	-1.750286000	-5.217944000
6	-3.776912000	-0.616642000	-6.105305000
6	-4.667862000	0.244200000	-5.529102000
6	-5.101564000	-0.356761000	-4.287224000
7	-4.474400000	-1.584458000	-4.121288000

6	-6.016619000	0.211758000	-3.415969000
6	-6.469694000	-0.380638000	-2.246942000
6	-7.515950000	0.154019000	-1.403932000
6	-7.740748000	-0.759019000	-0.411054000
6	-6.824249000	-1.857022000	-0.624109000
7	-6.047340000	-1.601223000	-1.743871000
1	-7.404171000	-3.043595000	1.032654000
1	-2.622557000	-6.640329000	-1.235145000
1	-2.179626000	-2.802158000	-6.333319000
1	-6.431769000	1.176836000	-3.684337000
8	-1.780212000	2.382013000	2.725060000
6	-1.283039000	1.855397000	1.681990000
8	-0.318539000	2.219576000	0.941876000
6	-2.283369000	-0.075705000	0.981825000
6	-3.645596000	0.161365000	1.021738000
6	-4.571483000	-0.110841000	2.173774000
1	-1.787961000	0.085283000	0.027770000
1	-4.067451000	0.732843000	0.194854000
1	-3.600794000	-1.441664000	-0.781921000
1	-4.603425000	0.743506000	2.869611000
1	-4.258788000	-0.985183000	2.755194000
8	-3.345776000	-1.896352000	-1.620483000
1	2.426668000	-2.014182000	-5.838447000
1	1.623783000	0.564237000	-3.394634000
1	-4.526266000	4.082836000	-1.662254000
1	-2.558191000	7.707690000	0.087633000
1	7.301220000	7.277635000	1.926893000
1	6.022695000	3.916428000	4.452368000
1	-8.458381000	-0.710013000	0.395056000
1	-6.272883000	-5.295084000	1.786164000
1	-5.005161000	1.203694000	-5.893064000
1	-8.016579000	1.096159000	-1.573982000
1	-4.224673000	-6.797214000	0.849049000
1	-1.124245000	-6.614838000	-3.400059000
1	-3.232981000	-0.505655000	-7.031616000
1	-0.974356000	-5.010258000	-5.583408000
6	-1.553741000	-0.999889000	1.898462000
6	-0.776230000	-2.034598000	1.339591000
6	-1.612748000	-0.891883000	3.302407000
6	-0.093414000	-2.941161000	2.156491000
1	-0.728902000	-2.133140000	0.259275000
6	-0.929744000	-1.800429000	4.117825000
1	-2.158519000	-0.066316000	3.743884000
6	-0.170037000	-2.830702000	3.550187000
1	0.495772000	-3.733817000	1.704442000
1	-0.984987000	-1.698913000	5.198273000
1	0.359244000	-3.535040000	4.185361000
1	-5.594727000	-0.281306000	1.819933000

$^{4}Pr_{beta,DC,S2}$

26	-3.183624000	-3.828212000	-1.971744000
16	-4.556884000	-5.719388000	-2.272405000
1	-5.239238000	-5.287027000	-3.393056000
6	5.987812000	3.835782000	1.537586000
6	5.994137000	5.002203000	0.512887000
6	5.039993000	6.180072000	0.820591000
6	5.308155000	6.805605000	2.203221000
6	5.149745000	7.240604000	-0.293026000
6	4.636398000	3.155696000	1.679087000
8	3.835042000	3.476594000	2.601857000
1	6.269436000	4.201478000	2.527744000
1	7.023622000	5.385593000	0.456008000
1	5.751947000	4.606439000	-0.483723000
1	4.010243000	5.794866000	0.811325000
1	4.668502000	7.682761000	2.357072000
1	5.103056000	6.098848000	3.013902000
1	6.351999000	7.138443000	2.287142000
1	4.440125000	8.058267000	-0.125250000
1	4.930860000	6.807883000	-1.276277000
1	6.160346000	7.670105000	-0.326513000
7	4.354236000	2.201720000	0.750999000
6	3.094862000	1.453626000	0.658147000
6	2.437643000	1.556637000	-0.738697000
6	1.923870000	2.942856000	-1.074826000
6	0.708063000	3.397158000	-0.532385000
6	0.252499000	4.696618000	-0.779359000
6	1.004044000	5.566870000	-1.582357000
6	2.200998000	5.114261000	-2.150833000
6	2.656772000	3.813452000	-1.898705000
1	5.054070000	2.008553000	0.047392000
1	2.427634000	1.854134000	1.423721000
1	3.158129000	1.223136000	-1.498890000
1	1.612508000	0.832609000	-0.761648000
1	0.111468000	2.728410000	0.084192000
1	-0.676845000	5.036767000	-0.332558000
1	0.663283000	6.586301000	-1.731783000
1	2.781129000	5.774550000	-2.788804000
1	3.585055000	3.469625000	-2.349477000
6	0.780890000	-0.774441000	-4.760001000
6	0.040080000	0.563661000	-4.545346000
6	-1.444799000	0.399291000	-4.177364000
6	0.996114000	-1.582776000	-3.468359000
1	0.215731000	-1.384093000	-5.481519000
1	0.554722000	1.136510000	-3.759070000
1	0.116944000	1.168193000	-5.460986000
1	-1.999000000	-0.080176000	-4.994335000
1	-1.578337000	-0.227939000	-3.288841000
1	-1.910987000	1.373433000	-3.984097000

1	1.530975000	-2.518377000	-3.673499000
1	0.049044000	-1.844345000	-2.984462000
1	1.596048000	-1.006717000	-2.749764000
6	-0.347106000	9.223315000	1.781117000
6	-0.053239000	8.002770000	2.696289000
6	1.065003000	7.100838000	2.139548000
6	1.320005000	5.815380000	2.939365000
7	0.153513000	4.909369000	2.884994000
6	0.143291000	3.670872000	3.391100000
7	1.294687000	3.078817000	3.801236000
7	-1.017214000	2.998462000	3.511380000
6	-0.850800000	8.813940000	0.402208000
8	-0.032566000	8.556461000	-0.528373000
1	0.577102000	9.788563000	1.618529000
1	-0.974172000	7.423009000	2.820985000
1	0.234926000	8.374612000	3.690231000
1	0.850323000	6.840299000	1.098951000
1	2.006428000	7.667144000	2.120804000
1	2.197818000	5.311009000	2.522700000
1	1.541544000	6.049278000	3.991002000
1	-0.713442000	5.247758000	2.425016000
1	2.213057000	3.349539000	3.442843000
1	1.243483000	2.230848000	4.342407000
1	-1.932185000	3.412393000	3.250732000
1	-1.017643000	2.002301000	3.672684000
7	-2.191635000	8.709620000	0.209053000
6	-2.725339000	8.229351000	-1.086297000
6	-4.242931000	8.437659000	-0.948467000
6	-4.497100000	8.241559000	0.563133000
6	-3.253176000	8.860852000	1.233458000
1	-2.470048000	7.170172000	-1.215622000
1	-4.516547000	9.455353000	-1.255723000
1	-4.812959000	7.736608000	-1.565366000
1	-5.424761000	8.711609000	0.904355000
1	-4.541254000	7.173698000	0.804120000
1	-2.989294000	8.328533000	2.149282000
1	-3.408661000	9.923556000	1.468476000
6	2.124757000	-3.151000000	-8.043993000
6	0.729694000	-3.488875000	-8.523314000
6	0.313577000	-4.823499000	-8.669301000
6	-0.975920000	-5.132842000	-9.121865000
6	-1.872849000	-4.107202000	-9.440701000
6	-1.470935000	-2.771463000	-9.299849000
6	-0.184329000	-2.467540000	-8.842717000
1	2.096640000	-2.508000000	-7.155999000
1	2.688158000	-4.053712000	-7.787367000
1	1.008885000	-5.625681000	-8.436414000
1	-1.275537000	-6.170807000	-9.228870000
1	-2.869664000	-4.344089000	-9.798864000
1	-2.156522000	-1.967837000	-9.552186000

1	0.117692000	-1.429340000	-8.735409000
6	-4.395452000	-3.809088000	1.252278000
6	-3.285537000	-4.593459000	0.983455000
6	-2.571082000	-5.367376000	1.973214000
6	-1.508379000	-5.949916000	1.341125000
6	-1.560431000	-5.543769000	-0.045245000
7	-2.658831000	-4.724028000	-0.247432000
6	-0.637791000	-5.916240000	-1.010105000
6	-0.691406000	-5.537875000	-2.340359000
6	0.239418000	-5.979432000	-3.356767000
6	-0.179599000	-5.450925000	-4.544099000
6	-1.369376000	-4.674762000	-4.268796000
7	-1.658581000	-4.728811000	-2.915101000
6	-2.102049000	-4.000235000	-5.230434000
6	-3.241061000	-3.255889000	-4.968672000
6	-3.967898000	-2.500153000	-5.963452000
6	-4.984250000	-1.854175000	-5.317147000
6	-4.896652000	-2.209576000	-3.918699000
7	-3.834332000	-3.084033000	-3.726905000
6	-5.745473000	-1.733229000	-2.932444000
6	-5.664075000	-2.065965000	-1.589643000
6	-6.600084000	-1.634384000	-0.575890000
6	-6.225309000	-2.225145000	0.598241000
6	-5.053492000	-3.024331000	0.320406000
7	-4.711206000	-2.897584000	-1.018933000
1	-4.768534000	-3.799664000	2.269809000
1	0.175329000	-6.564755000	-0.703492000
1	-1.755342000	-4.047532000	-6.256349000
1	-6.541207000	-1.062307000	-3.236334000
8	-3.445143000	3.947859000	2.560956000
6	-3.274438000	5.006208000	1.885108000
8	-2.237951000	5.712441000	1.662729000
6	-1.916649000	-0.021564000	0.839599000
6	-2.624510000	0.715472000	-0.048067000
6	-3.965664000	1.372503000	0.137468000
1	-0.986109000	-0.460182000	0.477835000
1	-2.174281000	0.846713000	-1.031662000
1	-2.514594000	-1.722767000	-1.031136000
1	-3.873524000	2.364623000	0.599865000
1	-4.627053000	0.779160000	0.778273000
8	-2.128585000	-2.386646000	-1.646483000
1	2.691760000	-2.613492000	-8.814878000
1	1.757828000	-0.568052000	-5.223888000
1	-2.271537000	8.788667000	-1.908371000
1	-1.060622000	9.887581000	2.281266000
1	6.741812000	3.096719000	1.235105000
1	3.286636000	0.398121000	0.894837000
1	-6.691321000	-2.142496000	1.569093000
1	-2.855966000	-5.438628000	3.012632000
1	-5.727455000	-1.188856000	-5.731904000

1	-7.435163000	-0.973588000	-0.756843000
1	-0.751018000	-6.596633000	1.759759000
1	1.089304000	-6.619400000	-3.169080000
1	-3.706832000	-2.471292000	-7.010960000
1	0.254822000	-5.564661000	-5.526309000
6	-2.229703000	-0.359137000	2.241070000
6	-1.627687000	-1.508922000	2.802210000
6	-3.067406000	0.421282000	3.072556000
6	-1.870415000	-1.883913000	4.125378000
1	-0.977865000	-2.118389000	2.180490000
6	-3.304033000	0.044071000	4.400652000
1	-3.508948000	1.338662000	2.701733000
6	-2.714191000	-1.109305000	4.932376000
1	-1.401410000	-2.777671000	4.526035000
1	-3.947565000	0.661356000	5.020004000
1	-2.902896000	-1.395940000	5.962300000
1	-4.460221000	1.503691000	-0.830552000

$^4TS_{gamma,HA,S2}$

26	-5.060478000	-2.040480000	-1.897327000
16	-6.090882000	-4.262334000	-2.370308000
1	-7.243066000	-3.826630000	-2.997930000
6	5.598159000	5.965800000	1.434993000
6	5.750115000	4.562547000	0.787368000
6	4.442282000	3.983411000	0.195056000
6	4.038316000	4.704010000	-1.106777000
6	4.569137000	2.468413000	-0.054515000
6	4.777031000	5.955679000	2.715868000
8	3.648458000	6.515579000	2.797062000
1	5.108247000	6.657366000	0.745895000
1	6.506917000	4.627091000	-0.007434000
1	6.146631000	3.860081000	1.534804000
1	3.638164000	4.125158000	0.932306000
1	3.076163000	4.327171000	-1.469672000
1	3.946367000	5.788996000	-0.974626000
1	4.787951000	4.526865000	-1.890189000
1	3.612945000	2.067486000	-0.406545000
1	4.840416000	1.934452000	0.863664000
1	5.335526000	2.257516000	-0.813347000
7	5.339954000	5.299859000	3.769397000
6	4.682246000	5.106129000	5.064375000
6	5.036545000	3.743268000	5.690750000
6	4.650484000	2.531287000	4.856714000
6	3.344757000	2.377003000	4.358049000
6	2.984109000	1.253500000	3.607854000
6	3.931032000	0.252546000	3.350498000
6	5.234214000	0.391450000	3.840332000
6	5.590549000	1.524402000	4.583741000
1	6.238518000	4.858206000	3.633115000

1	3.606535000	5.190918000	4.895421000
1	6.114964000	3.710911000	5.900770000
1	4.541596000	3.700480000	6.672109000
1	2.602087000	3.146364000	4.547172000
1	1.976020000	1.161781000	3.215326000
1	3.649239000	-0.620856000	2.771730000
1	5.974805000	-0.377724000	3.643352000
1	6.604991000	1.622827000	4.962836000
6	2.414515000	2.171328000	-4.424927000
6	1.306569000	3.068931000	-3.832949000
6	-0.034632000	3.002347000	-4.583667000
6	2.124116000	0.663614000	-4.331213000
1	2.573958000	2.445197000	-5.479479000
1	1.149557000	2.810775000	-2.775143000
1	1.660306000	4.110163000	-3.843285000
1	0.101829000	3.244740000	-5.647751000
1	-0.484100000	2.002489000	-4.523529000
1	-0.747693000	3.719251000	-4.162716000
1	2.952484000	0.072694000	-4.741980000
1	1.214394000	0.392287000	-4.880023000
1	1.980527000	0.358562000	-3.286587000
6	-2.472179000	6.802793000	-1.004943000
6	-1.435961000	6.559511000	0.122404000
6	-0.004840000	6.316918000	-0.390357000
6	1.016319000	6.137129000	0.747081000
7	0.772503000	4.882384000	1.481150000
6	0.619479000	4.778699000	2.801858000
7	1.030005000	5.784825000	3.630350000
7	0.051988000	3.671215000	3.320732000
6	-2.640191000	5.601367000	-1.926193000
8	-1.906500000	5.471927000	-2.945016000
1	-2.130510000	7.629338000	-1.636704000
1	-1.751007000	5.705836000	0.737645000
1	-1.437815000	7.439157000	0.783253000
1	0.017432000	5.443550000	-1.052615000
1	0.314718000	7.173128000	-1.000613000
1	2.033545000	6.129092000	0.346317000
1	0.954005000	6.974470000	1.448322000
1	0.708653000	3.995392000	0.906375000
1	1.871477000	6.309815000	3.388321000
1	0.686845000	5.826109000	4.576038000
1	-0.211192000	2.859827000	2.696441000
1	0.139059000	3.490389000	4.309085000
7	-3.575082000	4.666494000	-1.598579000
6	-3.776628000	3.462733000	-2.446093000
6	-5.058645000	2.822035000	-1.886068000
6	-5.068892000	3.262081000	-0.404910000
6	-4.489989000	4.690368000	-0.432875000
1	-2.910941000	2.795491000	-2.352166000
1	-5.940180000	3.220297000	-2.405431000

1	-5.050352000	1.732789000	-1.984684000
1	-6.065724000	3.229200000	0.045260000
1	-4.415983000	2.603496000	0.179764000
1	-3.953437000	4.940274000	0.487390000
1	-5.283785000	5.437826000	-0.579359000
6	0.016388000	-3.633568000	-8.163350000
6	-0.725299000	-2.315265000	-8.228721000
6	-1.906048000	-2.194695000	-8.984911000
6	-2.586302000	-0.975020000	-9.064907000
6	-2.101210000	0.149582000	-8.382129000
6	-0.931270000	0.042910000	-7.621852000
6	-0.250574000	-1.179764000	-7.549526000
1	0.861582000	-3.581495000	-7.469663000
1	-0.640053000	-4.448213000	-7.833650000
1	-2.290103000	-3.061429000	-9.516369000
1	-3.490071000	-0.900577000	-9.662647000
1	-2.625770000	1.097681000	-8.445816000
1	-0.547957000	0.906057000	-7.086808000
1	0.663479000	-1.250635000	-6.966061000
6	-6.679431000	-2.310018000	1.105112000
6	-5.406878000	-2.862118000	1.027781000
6	-4.732468000	-3.538738000	2.117216000
6	-3.539579000	-3.990263000	1.630342000
6	-3.468319000	-3.584333000	0.242311000
7	-4.619453000	-2.890708000	-0.109262000
6	-2.394803000	-3.822576000	-0.601277000
6	-2.301463000	-3.391931000	-1.921030000
6	-1.149293000	-3.592523000	-2.773826000
6	-1.433370000	-2.995765000	-3.970478000
6	-2.763562000	-2.435599000	-3.856892000
7	-3.276529000	-2.683808000	-2.597462000
6	-3.425421000	-1.770252000	-4.883598000
6	-4.715938000	-1.266400000	-4.820423000
6	-5.400286000	-0.620705000	-5.922852000
6	-6.652406000	-0.301293000	-5.481865000
6	-6.739378000	-0.743871000	-4.104486000
7	-5.550833000	-1.330226000	-3.716672000
6	-7.855019000	-0.594653000	-3.287048000
6	-7.933927000	-0.988908000	-1.961316000
6	-9.076695000	-0.780390000	-1.098769000
6	-8.735910000	-1.243113000	0.139764000
6	-7.383934000	-1.752141000	0.050512000
7	-6.908012000	-1.601635000	-1.247775000
1	-7.181128000	-2.354343000	2.066443000
1	-1.543837000	-4.355470000	-0.190613000
1	-2.890552000	-1.648780000	-5.819901000
1	-8.728084000	-0.114649000	-3.717296000
8	-0.299821000	1.517363000	1.763571000
6	0.236459000	1.546652000	0.586856000
8	0.751943000	2.600260000	0.052134000

6	0.207707000	0.282715000	-0.279156000
6	-1.225542000	0.127397000	-0.932858000
6	-2.354023000	-0.127917000	0.033529000
1	0.883081000	0.490715000	-1.115601000
1	-1.415541000	1.044155000	-1.505152000
1	-1.152509000	-0.696215000	-1.653400000
1	-2.673876000	0.722518000	0.636760000
1	-2.298836000	-1.055119000	0.603466000
8	-4.411191000	-0.433551000	-1.449596000
1	0.412840000	-3.917457000	-9.146823000
1	3.360357000	2.382830000	-3.905125000
1	-3.850099000	3.757264000	-3.496503000
1	-3.430049000	7.097465000	-0.560794000
1	6.594009000	6.374748000	1.656837000
1	4.965464000	5.910132000	5.758144000
1	-9.333745000	-1.249235000	1.039570000
1	-5.137016000	-3.654328000	3.112183000
1	-7.450190000	0.187232000	-6.022288000
1	-10.007213000	-0.329859000	-1.412364000
1	-2.767679000	-4.540856000	2.147498000
1	-0.248346000	-4.106597000	-2.473831000
1	-4.961391000	-0.449303000	-6.894572000
1	-0.817588000	-2.928660000	-4.855150000
6	0.676570000	-1.002930000	0.388349000
6	1.479033000	-1.889519000	-0.355354000
6	0.312586000	-1.376088000	1.697126000
6	1.903525000	-3.110585000	0.180371000
1	1.774703000	-1.615558000	-1.364760000
6	0.740355000	-2.596474000	2.235835000
1	-0.284067000	-0.688411000	2.283002000
6	1.533339000	-3.471046000	1.482043000
1	2.529185000	-3.772415000	-0.411967000
1	0.454035000	-2.862429000	3.249624000
1	1.864925000	-4.414893000	1.904940000
1	-3.497443000	-0.354357000	-0.768727000

^{4}IM1$_{gamma,HA,S2}$

26	-5.334257000	-2.162898000	-2.137349000
16	-6.052157000	-4.454404000	-2.704202000
1	-7.094648000	-4.140409000	-3.555812000
6	5.770066000	6.164019000	1.303814000
6	5.911838000	4.765743000	0.643053000
6	4.585524000	4.170916000	0.110002000
6	4.109145000	4.897259000	-1.163846000
6	4.721977000	2.659957000	-0.158785000
6	4.993288000	6.135741000	2.611612000
8	3.857340000	6.673775000	2.731492000
1	5.249837000	6.855003000	0.636757000
1	6.629911000	4.844429000	-0.185651000

1	6.352189000	4.066137000	1.368138000
1	3.817421000	4.293275000	0.888305000
1	3.142390000	4.501746000	-1.493219000
1	3.995950000	5.977809000	-1.013746000
1	4.829425000	4.748083000	-1.979884000
1	3.753252000	2.244347000	-0.455856000
1	5.055675000	2.125091000	0.737943000
1	5.445583000	2.466610000	-0.962897000
7	5.601890000	5.485994000	3.642785000
6	4.988644000	5.273218000	4.956518000
6	5.431046000	3.936297000	5.583941000
6	5.078412000	2.702926000	4.767480000
6	3.750838000	2.446303000	4.381890000
6	3.418329000	1.310211000	3.638145000
6	4.420023000	0.399671000	3.273691000
6	5.745550000	0.638928000	3.652220000
6	6.071315000	1.784415000	4.390656000
1	6.503361000	5.061345000	3.475391000
1	3.905291000	5.296695000	4.816613000
1	6.515635000	3.959840000	5.760035000
1	4.968246000	3.877031000	6.579637000
1	2.967309000	3.145904000	4.656696000
1	2.389183000	1.141088000	3.335962000
1	4.163828000	-0.483488000	2.697806000
1	6.526499000	-0.061956000	3.373368000
1	7.103193000	1.960228000	4.685309000
6	2.456444000	1.910414000	-4.213474000
6	1.386150000	2.866003000	-3.643662000
6	0.035847000	2.818494000	-4.378928000
6	2.110428000	0.418187000	-4.071184000
1	2.619464000	2.145362000	-5.276850000
1	1.232785000	2.651274000	-2.575602000
1	1.775086000	3.893419000	-3.694214000
1	0.168681000	3.029659000	-5.450233000
1	-0.440027000	1.833326000	-4.289259000
1	-0.653620000	3.564780000	-3.970121000
1	2.917415000	-0.216162000	-4.459047000
1	1.192425000	0.162162000	-4.613615000
1	1.953296000	0.154545000	-3.017193000
6	-2.410213000	6.657876000	-0.858262000
6	-1.361486000	6.415967000	0.257998000
6	0.071932000	6.217374000	-0.268438000
6	1.120075000	6.134826000	0.854973000
7	0.954156000	4.901751000	1.645521000
6	0.884088000	4.845725000	2.976003000
7	1.308347000	5.898073000	3.738012000
7	0.387382000	3.742502000	3.570916000
6	-2.580543000	5.465517000	-1.790657000
8	-1.811577000	5.317656000	-2.780764000
1	-2.078922000	7.493010000	-1.484952000

1	-1.654615000	5.546743000	0.862422000
1	-1.379146000	7.285572000	0.931945000
1	0.123947000	5.320729000	-0.896675000
1	0.341062000	7.062364000	-0.917257000
1	2.129509000	6.164377000	0.435499000
1	1.025526000	6.995589000	1.524294000
1	0.882674000	3.992309000	1.107693000
1	2.118184000	6.436384000	3.426799000
1	1.021945000	5.966640000	4.700931000
1	0.117027000	2.897574000	2.996070000
1	0.530784000	3.609233000	4.560145000
7	-3.563407000	4.565088000	-1.510407000
6	-3.798276000	3.391006000	-2.391006000
6	-5.157233000	2.842597000	-1.922755000
6	-5.208839000	3.236974000	-0.429556000
6	-4.521007000	4.616065000	-0.379353000
1	-2.993805000	2.657044000	-2.255768000
1	-5.971826000	3.334007000	-2.470991000
1	-5.240275000	1.760005000	-2.060981000
1	-6.226702000	3.268647000	-0.029456000
1	-4.640160000	2.510205000	0.162526000
1	-4.003761000	4.790693000	0.569563000
1	-5.249141000	5.427304000	-0.525380000
6	-0.133353000	-3.556300000	-8.079607000
6	-0.804844000	-2.205812000	-8.208292000
6	-1.963239000	-2.049221000	-8.989591000
6	-2.599607000	-0.806432000	-9.091908000
6	-2.089237000	0.305140000	-8.408572000
6	-0.935493000	0.164462000	-7.627451000
6	-0.298906000	-1.079329000	-7.533775000
1	0.941457000	-3.453119000	-7.895850000
1	-0.554522000	-4.133014000	-7.244523000
1	-2.364568000	-2.905692000	-9.524459000
1	-3.485654000	-0.704806000	-9.711810000
1	-2.578035000	1.270754000	-8.489847000
1	-0.530280000	1.019699000	-7.095969000
1	0.606399000	-1.174539000	-6.939617000
6	-7.116896000	-2.646067000	0.738155000
6	-5.780833000	-3.027447000	0.752185000
6	-5.103247000	-3.612556000	1.890937000
6	-3.827774000	-3.898912000	1.495924000
6	-3.709421000	-3.482439000	0.114984000
7	-4.914495000	-2.945630000	-0.325147000
6	-2.556200000	-3.577472000	-0.648513000
6	-2.426125000	-3.130164000	-1.960685000
6	-1.205233000	-3.185152000	-2.735948000
6	-1.481733000	-2.619767000	-3.949937000
6	-2.874546000	-2.225232000	-3.924613000
7	-3.433495000	-2.539167000	-2.699097000
6	-3.544569000	-1.642783000	-4.995498000

6	-4.890153000	-1.302565000	-5.024687000
6	-5.564031000	-0.713972000	-6.165077000
6	-6.872343000	-0.547774000	-5.810080000
6	-7.002799000	-1.030059000	-4.449477000
7	-5.787425000	-1.489992000	-3.988341000
6	-8.183118000	-1.023182000	-3.710879000
6	-8.309797000	-1.446818000	-2.398446000
6	-9.528874000	-1.391291000	-1.618997000
6	-9.222475000	-1.830469000	-0.363531000
6	-7.813937000	-2.169469000	-0.361183000
7	-7.271056000	-1.944338000	-1.618576000
1	-7.673304000	-2.764671000	1.662453000
1	-1.676467000	-4.003341000	-0.177358000
1	-2.965301000	-1.447233000	-5.891976000
1	-9.072832000	-0.636992000	-4.197905000
8	-0.002006000	1.524865000	2.115654000
6	0.421062000	1.533981000	0.895203000
8	0.892010000	2.574225000	0.295870000
6	0.282091000	0.268537000	0.043023000
6	-1.170731000	0.274060000	-0.609837000
6	-2.299857000	0.164906000	0.357567000
1	0.973063000	0.400153000	-0.795938000
1	-1.241988000	1.205621000	-1.184773000
1	-1.190106000	-0.560319000	-1.321863000
1	-2.703870000	1.053255000	0.832889000
1	-2.546844000	-0.791478000	0.808132000
8	-4.932261000	-0.453717000	-1.638612000
1	-0.265425000	-4.156603000	-8.986071000
1	3.412668000	2.102520000	-3.705277000
1	-3.781295000	3.706730000	-3.437385000
1	-3.364743000	6.944261000	-0.402905000
1	6.769373000	6.580584000	1.493198000
1	5.247018000	6.098561000	5.634792000
1	-9.876271000	-1.922769000	0.491527000
1	-5.560042000	-3.784129000	2.854714000
1	-7.680766000	-0.138342000	-6.398271000
1	-10.482557000	-1.050295000	-1.994952000
1	-3.028233000	-4.341340000	2.071558000
1	-0.270698000	-3.589018000	-2.376491000
1	-5.080805000	-0.471542000	-7.099888000
1	-0.821742000	-2.473225000	-4.792010000
6	0.601280000	-1.056213000	0.714786000
6	1.371633000	-1.999974000	0.007970000
6	0.118531000	-1.414972000	1.988894000
6	1.644963000	-3.264625000	0.542652000
1	1.762255000	-1.736311000	-0.971762000
6	0.392293000	-2.679256000	2.525117000
1	-0.440485000	-0.681740000	2.555098000
6	1.150582000	-3.612002000	1.805965000
1	2.248813000	-3.970925000	-0.020226000

1	0.016698000	-2.933272000	3.512490000
1	1.363481000	-4.589794000	2.227978000
1	-4.133838000	-0.416144000	-1.061658000

$^{4}Pr_{gamma,OH,S2}$

26	-5.241464000	-2.228926000	-2.359241000
16	-6.177554000	-4.398254000	-3.033655000
1	-5.162120000	-4.756434000	-3.900587000
6	5.736297000	6.174807000	1.215671000
6	5.871054000	4.730733000	0.659313000
6	4.540661000	4.101334000	0.178215000
6	4.057970000	4.734756000	-1.142133000
6	4.673976000	2.574624000	0.019202000
6	4.969868000	6.239747000	2.527837000
8	3.816279000	6.745763000	2.610500000
1	5.211202000	6.814837000	0.503181000
1	6.584782000	4.746630000	-0.176803000
1	6.313862000	4.086338000	1.432356000
1	3.777361000	4.282035000	0.949667000
1	3.092856000	4.311850000	-1.440750000
1	3.938646000	5.822446000	-1.067165000
1	4.777048000	4.532603000	-1.947737000
1	3.703777000	2.139312000	-0.243093000
1	5.010528000	2.105811000	0.951214000
1	5.394522000	2.323251000	-0.771471000
7	5.607607000	5.702782000	3.605866000
6	5.008039000	5.571881000	4.936371000
6	5.461712000	4.279908000	5.645101000
6	5.107453000	2.993081000	4.916462000
6	3.779865000	2.711440000	4.548819000
6	3.446561000	1.524721000	3.888910000
6	4.447314000	0.589323000	3.591547000
6	5.772987000	0.853698000	3.952260000
6	6.099518000	2.048814000	4.606618000
1	6.525470000	5.303520000	3.467632000
1	3.923797000	5.583503000	4.803020000
1	6.547858000	4.318972000	5.808463000
1	5.009301000	4.284523000	6.647422000
1	2.997287000	3.430622000	4.770230000
1	2.417292000	1.334168000	3.600155000
1	4.190894000	-0.332959000	3.080875000
1	6.553265000	0.133914000	3.724470000
1	7.131554000	2.244417000	4.887784000
6	2.498186000	1.357782000	-3.909493000
6	1.435198000	2.373567000	-3.438615000
6	0.099531000	2.293466000	-4.197173000
6	2.135499000	-0.110411000	-3.626931000
1	2.668598000	1.489855000	-4.989481000
1	1.255618000	2.245193000	-2.360907000

1	1.844034000	3.387320000	-3.559253000
1	0.256595000	2.441614000	-5.275893000
1	-0.387592000	1.319205000	-4.061450000
1	-0.590426000	3.068008000	-3.845749000
1	2.930275000	-0.788606000	-3.962273000
1	1.208588000	-0.404793000	-4.132860000
1	1.989556000	-0.271157000	-2.550711000
6	-2.477883000	6.316725000	-0.960264000
6	-1.436347000	6.135852000	0.174278000
6	0.011757000	5.978147000	-0.326334000
6	1.049702000	6.023090000	0.808840000
7	0.921638000	4.848996000	1.691033000
6	0.868328000	4.893382000	3.022786000
7	1.289345000	6.005218000	3.696197000
7	0.391660000	3.833685000	3.706060000
6	-2.605022000	5.094235000	-1.859599000
8	-1.751624000	4.885305000	-2.767119000
1	-2.159603000	7.143289000	-1.605960000
1	-1.709494000	5.270979000	0.793944000
1	-1.495576000	7.019755000	0.827235000
1	0.114664000	5.048460000	-0.896744000
1	0.245859000	6.793062000	-1.025253000
1	2.062108000	6.061923000	0.396835000
1	0.911958000	6.927251000	1.410538000
1	0.859038000	3.901878000	1.224007000
1	2.085350000	6.530600000	3.332036000
1	1.009341000	6.148526000	4.652704000
1	0.102088000	2.954865000	3.196719000
1	0.549462000	3.773959000	4.700123000
7	-3.645023000	4.241453000	-1.646579000
6	-3.847303000	3.050095000	-2.509760000
6	-5.310501000	2.663003000	-2.243746000
6	-5.514870000	3.059360000	-0.764244000
6	-4.705401000	4.365625000	-0.614758000
1	-3.167434000	2.246116000	-2.205361000
1	-5.981423000	3.235772000	-2.897677000
1	-5.490195000	1.598732000	-2.414906000
1	-6.567424000	3.200799000	-0.498729000
1	-5.093501000	2.273108000	-0.129821000
1	-4.273107000	4.470848000	0.386059000
1	-5.329613000	5.248843000	-0.812043000
6	-0.757930000	-3.423264000	-9.303742000
6	-1.198165000	-2.007994000	-8.996887000
6	-2.358510000	-1.470632000	-9.581156000
6	-2.778132000	-0.168654000	-9.281886000
6	-2.044665000	0.620304000	-8.386380000
6	-0.885869000	0.098715000	-7.797833000
6	-0.467017000	-1.201575000	-8.105356000
1	0.330221000	-3.527760000	-9.234911000
1	-1.199916000	-4.140716000	-8.598778000

1	-2.931912000	-2.074356000	-10.279330000
1	-3.668978000	0.232287000	-9.756779000
1	-2.365741000	1.630744000	-8.155037000
1	-0.309693000	0.703137000	-7.104394000
1	0.443866000	-1.592052000	-7.658547000
6	-6.695765000	-2.376853000	0.767906000
6	-5.388426000	-2.814192000	0.654564000
6	-4.634360000	-3.432900000	1.716594000
6	-3.406866000	-3.757153000	1.208935000
6	-3.394783000	-3.343019000	-0.172462000
7	-4.610023000	-2.737696000	-0.502088000
6	-2.331049000	-3.527475000	-1.037223000
6	-2.318839000	-3.127659000	-2.364141000
6	-1.182546000	-3.257806000	-3.242365000
6	-1.532679000	-2.702512000	-4.442432000
6	-2.888359000	-2.229354000	-4.313320000
7	-3.369609000	-2.502259000	-3.033784000
6	-3.587893000	-1.581539000	-5.317846000
6	-4.881227000	-1.107194000	-5.187459000
6	-5.615909000	-0.445611000	-6.239897000
6	-6.856141000	-0.158035000	-5.744620000
6	-6.892563000	-0.636937000	-4.382466000
7	-5.670514000	-1.211296000	-4.045964000
6	-7.987552000	-0.531277000	-3.542716000
6	-8.015697000	-0.963627000	-2.227875000
6	-9.144668000	-0.810674000	-1.343185000
6	-8.779236000	-1.323698000	-0.129952000
6	-7.423286000	-1.798438000	-0.258977000
7	-6.962038000	-1.576180000	-1.553719000
1	-7.185170000	-2.493727000	1.728289000
1	-1.434869000	-3.989332000	-0.639693000
1	-3.085263000	-1.428221000	-6.265772000
1	-8.883091000	-0.065343000	-3.938539000
8	-0.071620000	1.538559000	2.398296000
6	0.338918000	1.450566000	1.174412000
8	0.860753000	2.424649000	0.513527000
6	0.116594000	0.143891000	0.399690000
6	-1.310635000	0.183715000	-0.246719000
6	-2.477944000	0.283483000	0.738355000
1	0.819055000	0.187545000	-0.439150000
1	-1.350495000	1.045280000	-0.923822000
1	-1.430414000	-0.720503000	-0.857143000
1	-2.430206000	1.212547000	1.307076000
1	-2.466742000	-0.548902000	1.452240000
8	-3.760924000	0.320885000	0.030121000
1	-1.063523000	-3.728332000	-10.310170000
1	3.454234000	1.587000000	-3.416582000
1	-3.629418000	3.310478000	-3.547975000
1	-3.442959000	6.593172000	-0.523272000
1	6.737626000	6.602277000	1.365524000

1	5.268911000	6.439634000	5.558394000
1	-9.362532000	-1.378770000	0.777500000
1	-5.007916000	-3.592967000	2.717370000
1	-7.680030000	0.332404000	-6.242193000
1	-10.086330000	-0.362803000	-1.625045000
1	-2.571137000	-4.220070000	1.712353000
1	-0.241193000	-3.705333000	-2.959619000
1	-5.214795000	-0.239900000	-7.221221000
1	-0.938882000	-2.603369000	-5.338917000
6	0.384280000	-1.156130000	1.150509000
6	1.031735000	-2.200204000	0.460779000
6	-0.018112000	-1.392009000	2.479391000
6	1.261936000	-3.441333000	1.065268000
1	1.362276000	-2.032470000	-0.561478000
6	0.212592000	-2.633326000	3.085995000
1	-0.477873000	-0.582793000	3.031973000
6	0.847635000	-3.665159000	2.384181000
1	1.773708000	-4.225205000	0.513759000
1	-0.100314000	-2.790894000	4.114386000
1	1.029280000	-4.623947000	2.860797000
1	-4.015049000	-0.581733000	-0.255558000

$^{4}TS2_{gamma,DS,S2}$

26	-4.995139000	-2.247331000	-2.233589000
16	-6.067573000	-3.961412000	-3.357369000
1	-6.989790000	-3.199303000	-4.049186000
6	5.877090000	6.933676000	4.069188000
6	6.130446000	7.252450000	2.571924000
6	4.870566000	7.502173000	1.709711000
6	3.987556000	8.632642000	2.273218000
6	5.288344000	7.803265000	0.256464000
6	5.023068000	5.697320000	4.297286000
8	3.790775000	5.790922000	4.554658000
1	5.365137000	7.768687000	4.552479000
1	6.772711000	8.144424000	2.528615000
1	6.706939000	6.432038000	2.120612000
1	4.276517000	6.576335000	1.698280000
1	3.148509000	8.836006000	1.597263000
1	3.570584000	8.370028000	3.250811000
1	4.561704000	9.563605000	2.377934000
1	4.408574000	7.924464000	-0.385443000
1	5.895952000	6.991042000	-0.160207000
1	5.875662000	8.729807000	0.201403000
7	5.669081000	4.503339000	4.196424000
6	5.040848000	3.186882000	4.357207000
6	5.327627000	2.234231000	3.172022000
6	4.646839000	2.631014000	1.876877000
6	3.300423000	2.291545000	1.652558000
6	2.642429000	2.689174000	0.483987000

6	3.325707000	3.437165000	-0.485851000
6	4.672657000	3.762665000	-0.283451000
6	5.327680000	3.361124000	0.888365000
1	6.657715000	4.518472000	3.985663000
1	3.967230000	3.355721000	4.462109000
1	6.415115000	2.165214000	3.024931000
1	4.995319000	1.233107000	3.476719000
1	2.760894000	1.707256000	2.393879000
1	1.597281000	2.433857000	0.339932000
1	2.797069000	3.774942000	-1.371217000
1	5.213373000	4.328070000	-1.036698000
1	6.377027000	3.608931000	1.031818000
6	2.854814000	-0.001035000	-4.589911000
6	2.302698000	1.144521000	-3.715054000
6	1.825532000	2.374780000	-4.506052000
6	1.800118000	-0.669898000	-5.488185000
1	3.674482000	0.386078000	-5.214428000
1	1.475222000	0.758363000	-3.100732000
1	3.081889000	1.457181000	-3.006264000
1	2.642440000	2.789242000	-5.112412000
1	1.004746000	2.122279000	-5.189280000
1	1.469023000	3.162287000	-3.832385000
1	2.234405000	-1.500111000	-6.058858000
1	1.373447000	0.037961000	-6.208066000
1	0.974005000	-1.071349000	-4.886402000
6	-0.857557000	6.445917000	-0.850429000
6	-0.638941000	6.210940000	0.669864000
6	0.821416000	5.849664000	0.988843000
6	1.124665000	5.586846000	2.467873000
7	0.420972000	4.389262000	2.965959000
6	0.439739000	4.016020000	4.249932000
7	1.255033000	4.646407000	5.137658000
7	-0.358968000	3.018313000	4.675164000
6	-0.657427000	5.171909000	-1.662039000
8	0.491957000	4.840170000	-2.070856000
1	-0.121037000	7.170323000	-1.214057000
1	-1.302247000	5.409212000	1.017832000
1	-0.923527000	7.125025000	1.210610000
1	1.121317000	4.981297000	0.397371000
1	1.472539000	6.674034000	0.665713000
1	2.206568000	5.456241000	2.585578000
1	0.832489000	6.450018000	3.082693000
1	-0.077342000	3.771647000	2.282669000
1	2.086017000	5.165518000	4.850460000
1	1.137011000	4.479847000	6.123550000
1	-0.982933000	2.488649000	4.013186000
1	-0.217192000	2.628726000	5.593753000
7	-1.744237000	4.387479000	-1.893720000
6	-1.594198000	3.071196000	-2.560224000
6	-3.039154000	2.556477000	-2.698566000

6	-3.792503000	3.245009000	-1.536566000
6	-3.128691000	4.631027000	-1.428787000
1	-0.975476000	2.423530000	-1.927713000
1	-3.465015000	2.864189000	-3.661135000
1	-3.097164000	1.465719000	-2.643414000
1	-4.871199000	3.315964000	-1.708075000
1	-3.630084000	2.695796000	-0.602006000
1	-3.141343000	5.011349000	-0.404487000
1	-3.630128000	5.364649000	-2.076585000
6	-4.217707000	-4.995262000	-7.158353000
6	-3.240776000	-4.057279000	-7.831057000
6	-3.693426000	-2.921060000	-8.526206000
6	-2.790915000	-2.029408000	-9.117646000
6	-1.411845000	-2.257833000	-9.026414000
6	-0.946686000	-3.388755000	-8.344230000
6	-1.853908000	-4.278701000	-7.755916000
1	-3.791538000	-5.996789000	-7.037169000
1	-4.493547000	-4.633854000	-6.157819000
1	-4.761510000	-2.735784000	-8.600611000
1	-3.163706000	-1.162465000	-9.655864000
1	-0.710431000	-1.569780000	-9.488147000
1	0.120080000	-3.581986000	-8.276872000
1	-1.485660000	-5.155711000	-7.230677000
6	-7.081907000	-2.795999000	0.416261000
6	-5.888290000	-3.502015000	0.387249000
6	-5.481797000	-4.463688000	1.392594000
6	-4.255435000	-4.937555000	1.022421000
6	-3.900424000	-4.272813000	-0.214301000
7	-4.905146000	-3.395149000	-0.583931000
6	-2.734859000	-4.499535000	-0.931762000
6	-2.415988000	-3.883988000	-2.131392000
6	-1.222808000	-4.151967000	-2.906732000
6	-1.288894000	-3.372710000	-4.026111000
6	-2.522779000	-2.615463000	-3.944791000
7	-3.198111000	-2.939873000	-2.779226000
6	-2.943441000	-1.705594000	-4.903708000
6	-4.117067000	-0.966894000	-4.853258000
6	-4.529786000	-0.015155000	-5.864032000
6	-5.730496000	0.501173000	-5.465365000
6	-6.070499000	-0.133454000	-4.209339000
7	-5.067068000	-1.025766000	-3.844619000
6	-7.234724000	0.097067000	-3.490673000
6	-7.569930000	-0.536862000	-2.300730000
6	-8.796451000	-0.326471000	-1.563529000
6	-8.752448000	-1.146199000	-0.470522000
6	-7.497552000	-1.865223000	-0.525411000
7	-6.782776000	-1.477218000	-1.648998000
1	-7.751524000	-2.990964000	1.247415000
1	-2.027803000	-5.217258000	-0.530545000
1	-2.306564000	-1.567643000	-5.770675000

1	-7.941545000	0.814198000	-3.894905000
8	-1.725274000	1.480242000	2.949169000
6	-1.426760000	1.643535000	1.709635000
8	-0.818199000	2.664626000	1.216107000
6	-1.791859000	0.502447000	0.714109000
6	-3.215458000	-0.023017000	1.001384000
6	-3.429531000	-1.007530000	2.078249000
1	-1.800729000	0.975498000	-0.272076000
1	-3.911581000	0.831379000	1.066152000
1	-3.608180000	-0.537777000	-0.145509000
1	-3.214266000	-0.708852000	3.101505000
1	-3.747107000	-2.026025000	1.885444000
8	-4.133143000	-0.693083000	-1.291913000
1	-5.144182000	-5.089015000	-7.735984000
1	3.300976000	-0.763735000	-3.935209000
1	-1.082136000	3.188134000	-3.518627000
1	-1.852251000	6.872001000	-1.020296000
1	6.845567000	6.808422000	4.572110000
1	5.402718000	2.729145000	5.288422000
1	-9.493448000	-1.265026000	0.306766000
1	-6.069805000	-4.730097000	2.258922000
1	-6.344559000	1.235415000	-5.967042000
1	-9.580264000	0.356591000	-1.857761000
1	-3.639177000	-5.669135000	1.524381000
1	-0.451016000	-4.850632000	-2.618611000
1	-3.965139000	0.205461000	-6.758097000
1	-0.588999000	-3.307791000	-4.846116000
6	-0.708875000	-0.572164000	0.710530000
6	-0.223472000	-1.067913000	-0.510605000
6	-0.178635000	-1.087859000	1.907467000
6	0.766868000	-2.056820000	-0.539809000
1	-0.631344000	-0.686442000	-1.441796000
6	0.810702000	-2.076436000	1.878219000
1	-0.536653000	-0.695976000	2.852592000
6	1.287898000	-2.564321000	0.655493000
1	1.125323000	-2.427232000	-1.494705000
1	1.210969000	-2.464038000	2.810855000
1	2.059552000	-3.328232000	0.634881000
1	-4.745662000	0.068576000	-1.386136000

$^4Pr_{gamma,DS,S2}$

26	-5.340897000	-2.244409000	-2.910815000
16	-6.033887000	-3.322099000	-5.059864000
1	-6.118623000	-4.626910000	-4.610925000
6	6.053912000	4.745381000	0.466892000
6	6.110644000	3.247996000	0.873947000
6	4.739521000	2.573660000	1.126103000
6	3.913736000	2.436540000	-0.168333000
6	4.947956000	1.191984000	1.777488000

6	5.554538000	5.658130000	1.573365000
8	4.384464000	6.126748000	1.585633000
1	5.388439000	4.881450000	-0.388252000
1	6.630249000	2.699689000	0.075269000
1	6.735392000	3.146656000	1.774441000
1	4.173507000	3.199367000	1.833010000
1	2.924277000	2.013420000	0.034098000
1	3.757347000	3.402194000	-0.661916000
1	4.423591000	1.773865000	-0.880418000
1	3.988754000	0.695755000	1.958312000
1	5.481374000	1.274384000	2.734094000
1	5.536467000	0.537612000	1.120718000
7	6.452471000	5.942531000	2.563743000
6	6.180745000	6.853245000	3.679121000
6	6.274255000	6.185083000	5.073897000
6	5.123822000	5.262371000	5.427911000
6	3.998624000	5.761071000	6.110953000
6	2.939773000	4.916508000	6.468287000
6	2.988200000	3.553440000	6.140979000
6	4.097313000	3.047427000	5.450844000
6	5.155031000	3.895387000	5.101796000
1	7.385284000	5.561403000	2.484437000
1	5.182135000	7.262661000	3.510714000
1	7.225204000	5.639189000	5.140736000
1	6.330680000	6.992589000	5.815126000
1	3.962846000	6.812073000	6.385276000
1	2.093822000	5.314914000	7.020143000
1	2.184435000	2.889484000	6.445601000
1	4.145200000	1.993785000	5.195912000
1	6.018645000	3.489271000	4.584249000
6	2.057553000	1.108218000	-3.794398000
6	0.715134000	1.808172000	-3.495049000
6	0.614986000	3.244658000	-4.037229000
6	2.347071000	0.902890000	-5.291804000
1	2.873332000	1.694472000	-3.343496000
1	-0.101857000	1.203497000	-3.919961000
1	0.556399000	1.820392000	-2.408120000
1	1.431883000	3.865964000	-3.642847000
1	0.679562000	3.273833000	-5.132273000
1	-0.331477000	3.713022000	-3.746235000
1	3.281806000	0.348914000	-5.444991000
1	2.439017000	1.860087000	-5.818130000
1	1.538735000	0.338393000	-5.776137000
6	-2.301454000	6.580373000	-1.009019000
6	-1.069622000	6.427888000	-0.079701000
6	0.171392000	5.847420000	-0.781008000
6	1.320083000	5.556501000	0.197444000
7	0.960239000	4.453692000	1.104531000
6	1.230665000	4.419665000	2.415134000
7	2.084730000	5.311739000	2.979688000

7	0.629898000	3.496103000	3.191059000
6	-2.677685000	5.265944000	-1.680357000
8	-2.264635000	5.000769000	-2.842818000
1	-2.069976000	7.281645000	-1.816523000
1	-1.327277000	5.783719000	0.770691000
1	-0.825562000	7.414847000	0.339890000
1	-0.091008000	4.926624000	-1.314022000
1	0.532901000	6.550098000	-1.543698000
1	2.233943000	5.305234000	-0.353237000
1	1.542049000	6.446565000	0.795405000
1	0.511414000	3.599062000	0.689043000
1	2.823608000	5.754365000	2.434025000
1	2.176247000	5.340377000	3.983932000
1	-0.086487000	2.835965000	2.800814000
1	0.998822000	3.317867000	4.112164000
7	-3.407065000	4.364278000	-0.967038000
6	-3.689746000	3.017120000	-1.524937000
6	-4.716007000	2.427584000	-0.543027000
6	-4.364798000	3.106329000	0.800403000
6	-3.983904000	4.542218000	0.386135000
1	-2.759455000	2.435847000	-1.550463000
1	-5.733103000	2.704208000	-0.850717000
1	-4.663739000	1.336851000	-0.509933000
1	-5.191011000	3.092947000	1.518552000
1	-3.500950000	2.616036000	1.265701000
1	-3.263341000	4.991212000	1.074210000
1	-4.870184000	5.192892000	0.344608000
6	-2.935578000	-3.480182000	-7.796487000
6	-2.320686000	-2.118590000	-8.031557000
6	-3.138204000	-0.994678000	-8.256740000
6	-2.580478000	0.269497000	-8.473234000
6	-1.189114000	0.439128000	-8.465956000
6	-0.363654000	-0.669319000	-8.244572000
6	-0.927356000	-1.934300000	-8.030361000
1	-2.170976000	-4.224092000	-7.549288000
1	-3.665208000	-3.455477000	-6.977155000
1	-4.217715000	-1.115866000	-8.251540000
1	-3.230570000	1.122113000	-8.647273000
1	-0.755515000	1.420123000	-8.633943000
1	0.716267000	-0.552039000	-8.246751000
1	-0.280292000	-2.791099000	-7.861984000
6	-7.702486000	-3.956434000	-1.065120000
6	-6.441869000	-4.524747000	-1.188599000
6	-6.057881000	-5.795398000	-0.619369000
6	-4.740340000	-5.996404000	-0.929268000
6	-4.302286000	-4.852253000	-1.693560000
7	-5.357563000	-3.961091000	-1.852578000
6	-3.013621000	-4.673745000	-2.176686000
6	-2.579957000	-3.570609000	-2.897120000
6	-1.246068000	-3.409279000	-3.436118000

6	-1.225531000	-2.221127000	-4.111896000
6	-2.545686000	-1.640230000	-3.991259000
7	-3.358120000	-2.472763000	-3.234867000
6	-2.934588000	-0.444091000	-4.573176000
6	-4.207155000	0.101414000	-4.489314000
6	-4.594063000	1.364812000	-5.076413000
6	-5.908869000	1.568105000	-4.760129000
6	-6.340096000	0.431252000	-3.976825000
7	-5.289160000	-0.461445000	-3.828811000
6	-7.612664000	0.282128000	-3.444082000
6	-8.032305000	-0.788422000	-2.670184000
6	-9.373749000	-0.961170000	-2.157471000
6	-9.400814000	-2.153723000	-1.489611000
6	-8.075629000	-2.725994000	-1.584861000
7	-7.244205000	-1.868230000	-2.294078000
1	-8.454345000	-4.523114000	-0.526206000
1	-2.293682000	-5.461529000	-1.982108000
1	-2.194419000	0.099260000	-5.148945000
1	-8.337326000	1.060248000	-3.658945000
8	-0.990136000	1.605207000	2.131722000
6	-0.617526000	1.296478000	0.935008000
8	-0.009977000	2.089213000	0.125978000
6	-0.854077000	-0.155130000	0.456367000
6	-1.985819000	-0.827088000	1.222371000
6	-2.451330000	-2.063966000	0.984405000
1	-1.102659000	-0.099966000	-0.609615000
1	-2.385543000	-0.231834000	2.040724000
1	-3.989835000	-1.279356000	-0.377774000
1	-3.227504000	-2.504963000	1.605621000
1	-2.035557000	-2.705595000	0.210718000
8	-4.803115000	-1.105943000	-0.903990000
1	-3.468813000	-3.833672000	-8.688962000
1	2.069685000	0.132646000	-3.286551000
1	-4.058342000	3.101178000	-2.549582000
1	-3.141965000	6.991953000	-0.436571000
1	7.056700000	5.069605000	0.156819000
1	6.896842000	7.684290000	3.636970000
1	-10.235344000	-2.618871000	-0.984956000
1	-6.717739000	-6.438952000	-0.055431000
1	-6.537847000	2.406344000	-5.022781000
1	-10.182287000	-0.260142000	-2.306424000
1	-4.112475000	-6.836050000	-0.667915000
1	-0.445811000	-4.124985000	-3.315234000
1	-3.933090000	2.002045000	-5.644784000
1	-0.412370000	-1.774173000	-4.664251000
6	0.454526000	-0.944346000	0.588532000
6	1.039578000	-1.558535000	-0.528583000
6	1.073134000	-1.086305000	1.844135000
6	2.217269000	-2.306263000	-0.395757000
1	0.569424000	-1.457259000	-1.501924000

6	2.246852000	-1.833757000	1.976824000
1	0.625491000	-0.605608000	2.708549000
6	2.823688000	-2.448279000	0.856263000
1	2.657241000	-2.776083000	-1.270454000
1	2.709304000	-1.943343000	2.953748000
1	3.733681000	-3.031354000	0.960636000
1	-5.597765000	-1.131262000	-0.340393000

Full Substrate (S2)

8	-0.476034000	1.184052000	1.978995000
6	-0.659509000	1.118825000	0.703472000
8	-0.986334000	2.061519000	-0.091517000
6	-0.584712000	-0.305297000	0.033564000
6	-1.958751000	-1.025274000	0.242653000
6	-2.298743000	-1.369272000	1.701686000
1	-0.508365000	-0.109692000	-1.043693000
1	-2.726018000	-0.351826000	-0.164596000
1	-1.976396000	-1.943707000	-0.367825000
1	-2.193779000	-0.475438000	2.323768000
1	-1.615376000	-2.130781000	2.097674000
6	0.586285000	-1.192315000	0.424224000
6	1.154534000	-2.050878000	-0.539041000
6	1.131367000	-1.213163000	1.725472000
6	2.217432000	-2.905583000	-0.223985000
1	0.755704000	-2.040525000	-1.551534000
6	2.195444000	-2.065625000	2.040519000
1	0.714494000	-0.507939000	2.438102000
6	2.742972000	-2.919910000	1.073619000
1	2.636777000	-3.554226000	-0.990403000
1	2.604553000	-2.059392000	3.048706000
1	3.570046000	-3.580177000	1.324913000
1	-3.323793000	-1.758167000	1.783801000

Substrate_S2_C2_alpha (Phenyl_Butanoic_acid_non_fix remove_alpha)

8	-1.946550000	1.368174000	1.689646000
6	-0.725201000	0.991846000	1.515082000
8	0.328266000	1.633643000	1.885968000
6	-0.573404000	-0.344848000	0.794463000
6	-1.829267000	-0.841436000	0.112909000
6	-2.734571000	-1.680165000	1.049615000
1	-2.402780000	0.042101000	-0.181943000
1	-1.592609000	-1.421265000	-0.791642000
1	-3.020094000	-1.047696000	1.894977000
1	-2.212683000	-2.568168000	1.428970000
6	0.618316000	-1.155322000	0.808962000

6	0.636493000	-2.477848000	0.259585000
6	1.848704000	-0.688624000	1.377453000
6	1.786037000	-3.265876000	0.277531000
1	-0.268761000	-2.888627000	-0.173453000
6	2.987365000	-1.487853000	1.389893000
1	1.831704000	0.319010000	1.780980000
6	2.976388000	-2.782757000	0.841587000
1	1.754748000	-4.267089000	-0.148136000
1	3.903737000	-1.098426000	1.829136000
1	3.872340000	-3.398755000	0.853162000
1	-3.644878000	-2.011803000	0.529175000

Substrate_S2_C3_beta (Phenyl_Butanoic_acid_non_fix remove_beta)

8	-0.542710000	1.219052000	1.976886000
6	-0.564580000	1.174603000	0.693870000
8	-0.598865000	2.125583000	-0.147451000
6	-0.623025000	-0.306955000	0.037819000
6	-1.958355000	-0.919727000	0.348977000
6	-2.295016000	-1.413393000	1.722989000
1	-0.567326000	-0.120825000	-1.040197000
1	-2.783848000	-0.599715000	-0.288410000
1	-2.196160000	-0.588981000	2.450075000
1	-1.603029000	-2.200912000	2.057924000
6	0.557915000	-1.189670000	0.426704000
6	1.124852000	-2.050146000	-0.534343000
6	1.111137000	-1.195217000	1.723226000
6	2.201341000	-2.887853000	-0.221228000
1	0.710549000	-2.058019000	-1.539873000
6	2.186490000	-2.034258000	2.038035000
1	0.694368000	-0.496158000	2.441329000
6	2.737620000	-2.886870000	1.072421000
1	2.621334000	-3.538361000	-0.985625000
1	2.601372000	-2.016775000	3.043611000

1	3.574240000	-3.535659000	1.321586000
1	-3.316408000	-1.813624000	1.773601000

Substrate_S2_C4_beta (Phenyl_Butanoic_acid_non_fix remove_gamma)

8	-0.374974000	1.220437000	1.926280000
6	-0.636874000	1.113499000	0.668929000
8	-1.044523000	2.020215000	-0.130768000
6	-0.559106000	-0.327027000	0.030206000
6	-1.918507000	-1.058825000	0.314302000
6	-2.226434000	-1.271976000	1.760708000
1	-0.526152000	-0.158886000	-1.053226000
1	-2.695097000	-0.423330000	-0.154405000
1	-1.937024000	-2.018515000	-0.228794000
1	-1.894887000	-0.531749000	2.480875000
1	-2.828313000	-2.117540000	2.086096000
6	0.630934000	-1.190083000	0.407490000
6	1.221547000	-2.018751000	-0.567389000
6	1.166096000	-1.225179000	1.712010000
6	2.297343000	-2.860727000	-0.260563000
1	0.828873000	-1.997500000	-1.582176000
6	2.242742000	-2.064303000	2.019136000
1	0.740500000	-0.537597000	2.434736000
6	2.812574000	-2.890710000	1.040646000
1	2.733271000	-3.487481000	-1.035863000
1	2.644650000	-2.069293000	3.030187000
1	3.648951000	-3.541302000	1.286356000
